# A Stretchable, Transparent, Photothermally Stimulated Laser-Induced Graphene Patch for Noninvasive Skin Tumor Treatment

**DOI:** 10.1021/acsnano.5c21102

**Published:** 2026-03-05

**Authors:** Xiaoyu Xu, Le Cheng, Baoping Li, Xinyu Wang, Siyu Chen, Zihao Li, Li Zhou, Tengyue Liu, Yidan Zhou, Zhiqiang Li, Xin Li, Shi Chen, Meijia Gu, Ruquan Ye

**Affiliations:** 1 Department of Neurosurgery, Zhongnan Hospital of Wuhan University, Ministry of Education Key Laboratory of Combinatorial Biosynthesis and Drug Discovery, School of Pharmaceutical Sciences, 12390 Wuhan University, Wuhan, Hubei 430071, China; 2 Department of Chemistry, State Key Laboratory of Marine Environmental Health, 53025 City University of Hong Kong, Hong Kong 999077, China; 3 Department of Gynecology, Renmin Hospital of Wuhan University& Ministry of Education Key Laboratory of Combinatorial Biosynthesis and Drug Discovery, School of Pharmaceutical Sciences, 12390 Wuhan University, Wuhan 430071, China; 4 Center for Animal Experiment, Wuhan University School of Medicine, 12390 Wuhan University, Wuhan, Hubei 430071, China; 5 Department of Epidemiology and Biostatistics, School of Public Health, 12390 Wuhan University, Wuhan 430071, China; 6 Department of Critical Care Medicine, Intensive Care Unit, Shenzhen Key Laboratory of Microbiology in Genomic Modification & Editing and Application, Shenzhen Institute of Translational Medicine, Shenzhen University Medical School, Shenzhen Second People’s Hospital, The First Affiliated Hospital of Shenzhen University, Shenzhen 518035, China

**Keywords:** laser-induced graphene (LIG), cuproptosis, ferroptosis, low-temperature phototherapy, melanoma

## Abstract

Melanoma causes over 80% of skin cancer-related deaths, with conventional therapies hampered by its aggressiveness, metastasis, and drug resistance. Noninvasive, biocompatible strategies are promising for next-generation cancer treatments. Herein, we developed a soft, stretchable laser-induced graphene (LIG)-Cu/PDMS patch, consisting of CuO-embedded LIG (active component) and biocompatible PDMS (flexible matrix). Chemically inert and breathable, the patch minimizes toxic side effects. Upon photothermal activation, it releases Cu^2+^ that accumulates in melanoma tissue. In a mouse model, two 1-h phototherapy sessions achieved effective tumor suppression within 10 days. Mechanistically, the patch enhances reactive oxygen species production, inducing apoptosis, cuproptosis, and ferroptosis. It also inhibits tumor invasion/metastasis and boosts antitumor immunity, with stable performance enabling multiple uses (energy-efficient and environmentally sustainable). This work demonstrates graphene-based materials’ potential in cancer therapy via synergistic activation of multiple cell death pathways and efficacy in low-temperature phototherapy, expanding graphene’s application in malignant tumor treatment and highlighting its clinical translation prospects.

## Graphical abstract

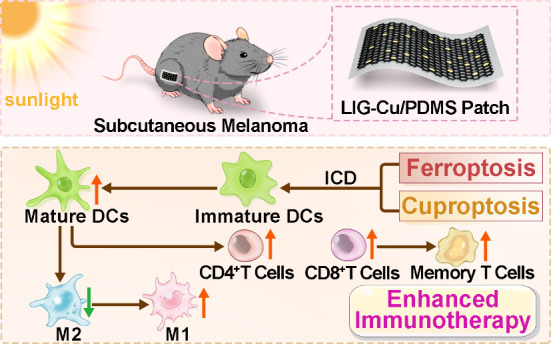

## Introduction

Melanoma is one of the most devastating human cancers, accounting for over 80% of skin cancer deaths.^1^ Conventional treatments, such as surgical resection and chemotherapy, often fail due to incomplete inspection of resection margins, high recurrence rates, systemic toxicity, and the development of resistance.^2^ Despite extensive efforts devoted to medical technology, therapeutic resistance and metastasis are major challenges in present cancer treatment.^3^ Melanoma, which predominantly affects the epidermis and dermis, highlights the limitations of current therapies. Surgical excision of superficial lesions and surrounding skin tissues remains the standard treatment for melanoma.^4^ However, physical excision can prolong postsurgery recovery, increase infection risk, and lead to inevitable resistance to chemotherapy. Although emerging immunotherapy shows promise in addressing melanoma, it involves time-consuming cell modification processes and complex treatment protocols. Additionally, melanoma’s resistance to radiotherapy, attributed to its minimal DNA damage and limited repair mechanisms, further complicates the treatment landscape.^5^ It is challenging to develop streamlined and effective treatment modalities that can inhibit melanoma while minimizing toxic side effects and adverse reactions.

Recent developments in nanotechnology have made significant advances in skin cancer therapy.^6^ For example, microneedles can efficiently traverse the cutaneous barrier, facilitating the direct delivery of therapeutics into the dermal layer, thus enhancing drug concentration within the skin.^7^ However, their production involves significant costs as well as the potential to induce localized inflammatory or hypersensitivity reactions.^8^ Additionally, minimally invasive therapeutic strategy may need repeated or long-time treatments, and even elicit cancer cells easily disseminate, promoting potential metastasis.^9^ Hydrogels, characterized by excellent biocompatibility, improve therapeutic outcomes through controlled drug release mechanisms.^10^ Smart patches have recently provided a promising approach for skin tumor treatment by external stimulation to trigger irreversible tumor cell damage.^11^ A variety of different electrical patches or hyperthermia have been developed for skin tumor treatment.^12^ These patches with complicated structures and fabrication procedures usually require extensive equipment and preparation difficulties, making their preparation expensive and time-consuming. Graphene and its derivatives have also garnered significant attention for various potential medical applications.^13^ The primary therapeutic role of graphene includes drug delivery, photothermal therapy (PTT), photodynamic therapy (PDT), and theranostics.^14^ PTT involves the use of photothermal agents (PTAs) to absorb photon energy and convert it into heat, effectively causing the photoablation of cancer cells and leading to their destruction. This method is less invasive than traditional treatments, thus minimizing toxicity.^15^ Nevertheless, high-temperature PTT carries an inherent risk of damaging nearby healthy tissues and may lead to inflammatory conditions, as controlling heat diffusion can be challenging.^16^ Mild PTT at 42 °C has garnered increasing interest due to its ability to provide considerable protection for normal tissues.^17^

Conventional methods for graphene preparation are often complex and offer limited control over patterning, which restricts their utility in medical applications. In recent years, laser-induced graphene (LIG) has become an increasingly attractive material,^18^ which is a 3D porous material generated by directing a laser onto various carbon precursors. It boasts impressive characteristics such as a high surface area of approximately 340 m^2^ g^–1^, exceptional thermal stability exceeding 900 °C, and outstanding conductivity ranging from 5 to 25 S cm^–1^.^19^ In addition, the formation of LIG-based metal hybrids can leverage the synergistic effect of graphene scaffolds and metal components, which has further expanded its application into diverse fields, including electrocatalysis,^20^ water treatment,^21^ antibacterial surfaces,^22,23^ wearable health monitoring,^24,25^ and biomedical soft robots.^26,27^ However, the limited stretchability of conventional LIG substrates like polyimide (PI) restricts their practical use in wearables. Due to these shortcomings, a simple, efficient, and safe patch is urgently needed for skin tumor treatment.

Herein, we have developed a facile method for preparing transparent and stretchable LIG-based hybrid patches for low-temperature. We use copper metal as the additive to the LIG hybrid, which can induce cuproptosis for improved cancer therapy (Scheme 1).^28^ The LIG hybrid is further transferred to the polydimethylsiloxane (PDMS) matrix via our cold-transfer method to form the LIG-Cu/PDMS patch. Due to the photothermal conversion performance, the LIG-Cu/PDMS patch with sunlight effectively suppresses tumor growth under mild photothermal conditions at 42 °C, offering considerable protection to adjacent healthy skin tissue. The PDMS matrix is stretchable and transparent, in addition to the potential for prolonging therapeutics release.^29^ As expected, the developed LIG-Cu/PDMS patch reduces melanoma invasion and metastasis. It instigates cuproptosis under the sunlight, thereby enhancing cancer treatment efficacy. Additionally, with sunlight irradiation, the LIG-Cu/PDMS patch simultaneously triggers tumor cell apoptosis and ferroptosis effectively. The generated large amounts of reactive oxygen species (ROS) promote cell death via mitochondrial oxidative stress. When cuproptosis and ferroptosis occur together, robust oxidative stress increases cell death, and the subsequent release of damage-associated molecular patterns (DAMPs) mediates enhanced immunotherapy by promoting the cross-activation of multiple cell death pathways. This cascade, characterized by the synergistic interaction between these pathways, significantly amplifies the overall therapeutic outcome.^30^ Multiple biomechanical, *in vitro*, and *in vivo* safety parameters demonstrate the feasibility of the LIG-Cu/PDMS composite patch for human use, illuminating its great potential in clinical applications for skin tumor treatment.

**Scheme 1:**
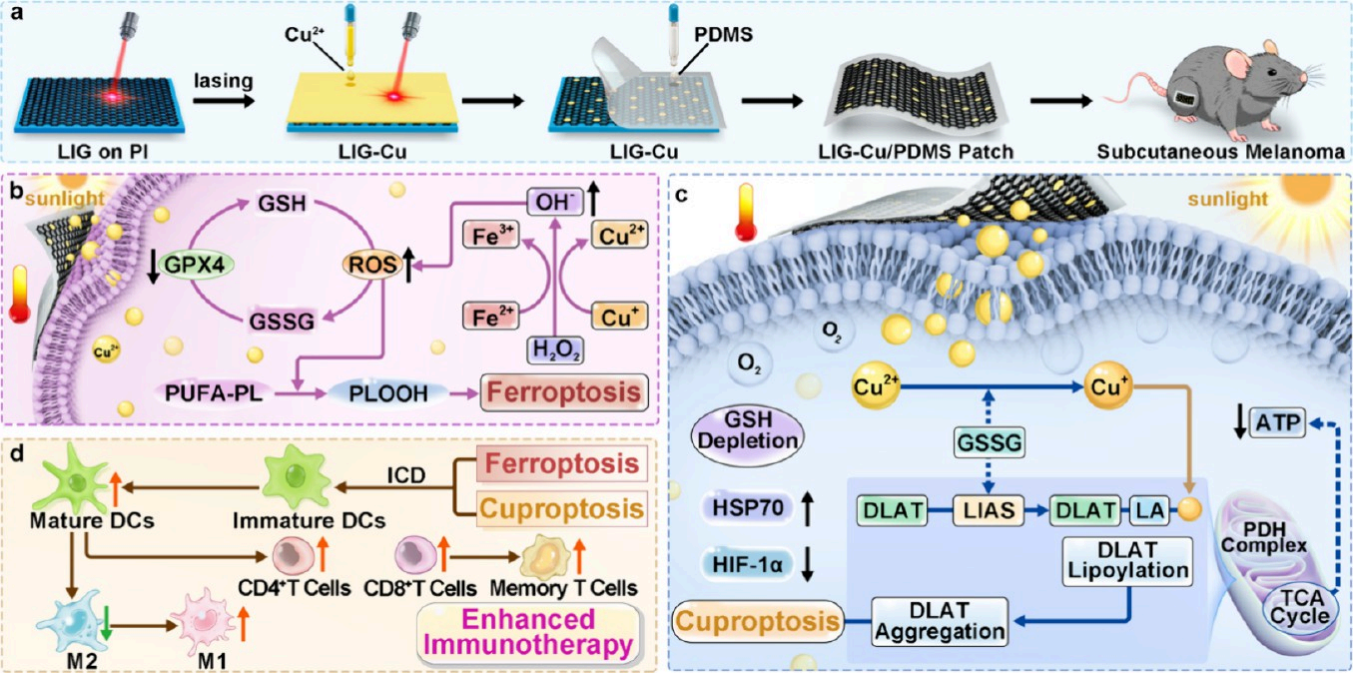
Schematic Diagram of the Preparation of LIG-Cu/PDMS (a) and the Antitumor Treatment Process Combining Mild Photothermal Therapy and Cu^2+^-Induced Ferroptosis (b), Cuproptosis (c), and Enhanced Immunotherapy (d) in Mouse Melanoma

## Results and Discussion

### Synthesis and Characterization of LIG-Cu/PDMS

As shown in Figure 1a, the LIG layer was formed by lasing a commercial PI film in ambient conditions. The laser irradiation generates high temperatures up to 2600 °C,^31^ leading to a swift release of gas that results in the formation of a porous structure (Figure S1). After curing at 80 °C for 2 h, the PDMS/PI bilayers were immersed in liquid nitrogen for 5 min to ensure complete freezing. While still in the frozen state, the PDMS layer was rapidly peeled from the PI substrate in a single motion with the aid of tweezers, leading to clean separation and the successful fabrication of intact LIG/PDMS or LIG-Cu/PDMS structures. Then copper salt precursor was loaded onto the prepared LIG, and a second laser irradiation transforms Cu salts into CuO,^21^ forming LIG-Cu hybrid (Figure 1b,c). The CuO nanoparticles are evenly dispersed with a diameter of hundreds of nanometers on the LIG sheets.

**Figure 1:**
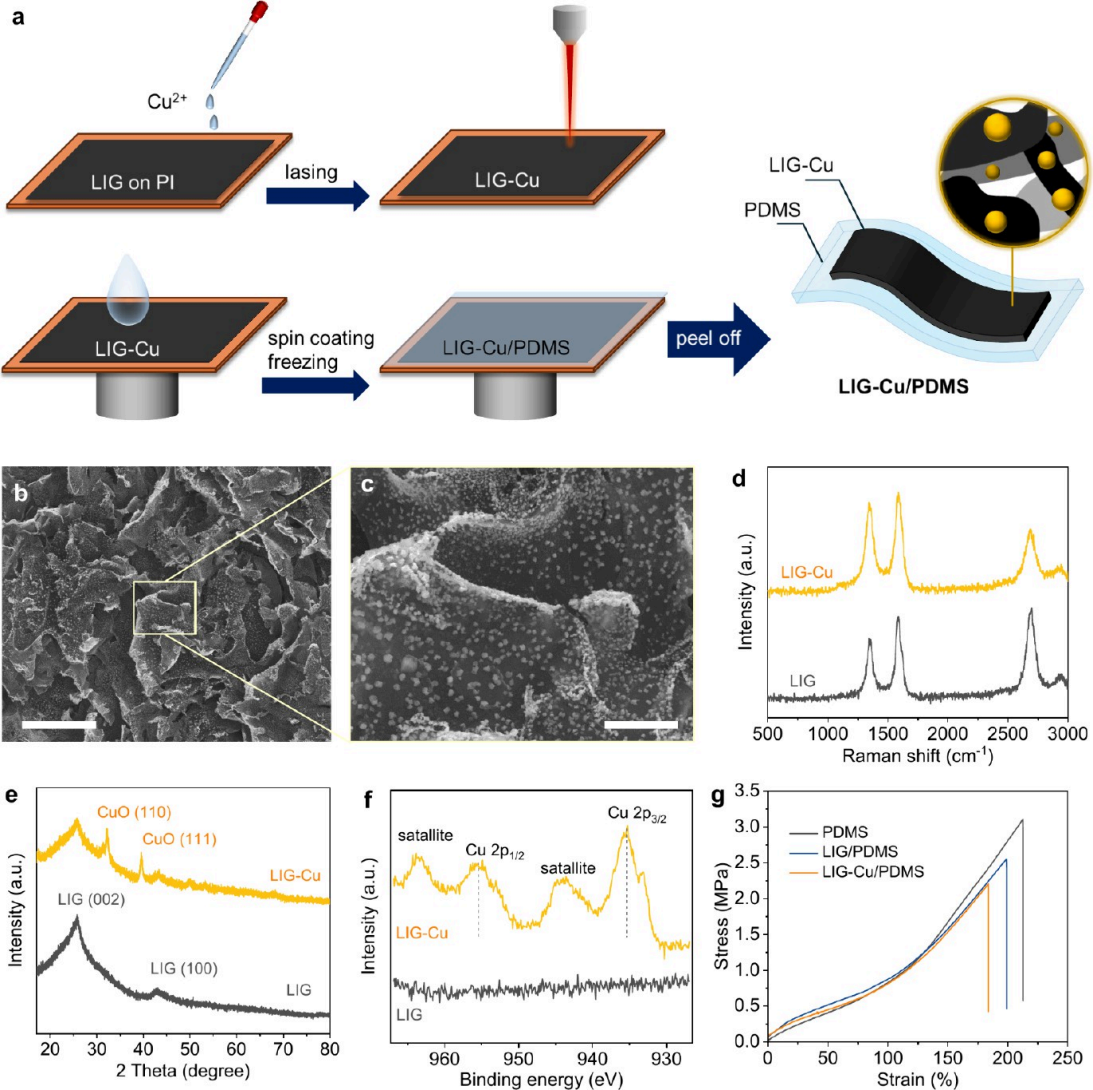
Synthesis and characterization of LIG-Cu/PDMS. a) Schematic of the fabrication process. b,c) SEM images of LIG-Cu. Scale bars: 30 μm for (b) and 5 μm for (c). d–f) Raman spectra­(d), XRD patterns­(e), and high-resolution­(f) Cu 2p XPS spectra of LIG and LIG-Cu. g) Stress–strain curves of pure PDMS, LIG/PDMS, and LIG-Cu/PDMS.

From the Raman spectra (Figure 1d), three characteristic bands of graphene, namely the D band at 1350 cm^–1^, the G band at 1580 cm^–1^, and the 2D band at 2700 cm^–1^, can be observed for both LIG and LIG-Cu, which verify the formation of graphene structure with a high graphitization degree.^32^ The *I*_D_/*I*_G_ and *I*_2D_/*I*_G_ intensity ratios can serve as indicators to estimate the defect density and the number of layers, respectively.^32^ The *I*_D_/*I*_G_ is 0.71 for LIG, which increases to 0.89 for LIG-Cu. This shift suggests higher defect densities after embedding CuO nanoparticles into the graphene sheets, potentially introducing more lattice disorder. *I*_2D_/*I*_G_ significantly decreases from 1.09 for LIG to 0.63 for LIG-Cu, indicating increased layer stacking following the second lasing process. These results are consistent with our previous findings.^33^ X-ray diffraction (XRD) patterns were collected to verify the crystal components. As shown in Figure 1e, broad (002) and (100) characteristic peaks can be observed at 2θ = 26° and 41° for the LIG samples, corresponding to an interlayer spacing of ∼ 3.4 Å.^34^ In the LIG-Cu sample, two new peaks emerge at 2θ = 32.2° and 39.6°, which comes from the (110) and (111) facets of CuO, respectively.^35^ X-ray photoelectron spectroscopy (XPS) was conducted to analyze the elemental compositions (Table S1) and chemical state of Cu in the LIG samples. Carbon and oxygen are the main elements in LIG. For LIG-Cu, the oxygen content increases slightly, and the copper content is 0.91%. In the high-resolution XPS spectrum of the Cu 2p region (Figure 1f), the LIG-Cu sample displays a distinct Cu 2p_3/2_ peak at 935 eV and Cu 2p_1/2_ peak at 955 eV, alongside strong Cu^2+^ satellite peaks at 944 and 963 eV, suggesting the +2 oxidized state of copper in the LIG-Cu sample.^36^

To form a soft and transparent patch, LIG was transferred from PI to PDMS. The thickness of the resulting LIG/PDMS and LIG-Cu/PDMS composite patches is ∼230 μm. Scanning electron microscope (SEM) images (Figure S2) reveal the smooth and dense surface of pure PDMS, while it becomes rough for LIG/PDMS and LIG-Cu/PDMS. Additionally, some unembedded LIG sheets and CuO nanoparticles are visible on the PDMS surface, which facilitates contact with skin for therapeutic applications. The mechanical properties of the patches are evaluated using tensile tests. As shown in Figure 1g, pure PDMS, LIG/PDMS, and LIG-Cu/PDMS demonstrate similar elastic moduli. Pure PDMS fractures at 212.6% strain; this value slightly decreases to 199.0% and 183.7% for LIG/PDMS and LIG-Cu/PDMS, respectively. This decrease can be attributed to the disruption of PDMS matrix integrity following composite formation, as evidenced by the rough surfaces observed in the SEM images. Despite this decrease, the strain range remains adequate for the majority of epidermic applications. In addition, photographs of the LIG-Cu/PDMS patch stretched demonstrate its intrinsic stretchability, highlighting the ability to achieve stable contact and uniform energy delivery, features that are critical for ensuring reproducible therapeutic efficacy (Figure S3).

### Antitumor Effect of LIG-Cu/PDMS Patches *In Vitro*

The *in vitro* antitumor therapeutic efficacy was investigated against B16–F10 cells. As shown in Figure 2a–c, with sunlight, cell death increases with irradiation time, and LIG-Cu/PDMS exhibits stronger cytotoxicity than LIG/PDMS, implying that copper might play a crucial role in induced cell death. After 60 min of light exposure with LIG-Cu/PDMS, almost all cells become dead. The cell Live/Dead staining assay also shows similar results. Calcein AM stains live cells green, while propidium iodide (PI) stains dead cells red. As shown in Figure 2d and Figure S4, confocal laser scanning microscopy (CLSM) images reveal that all groups of B16–F10 cells emit green fluorescence in the absence of sunlight. However, in the LIG-Cu/PDMS + Sunlight group, after phototherapy, nearly all cells are dead. We further evaluate the antitumor efficacy and stability of recycled LIG/PDMS and LIG-Cu/PDMS against melanoma cells. Figure S5 demonstrates the outstanding stability of LIG/PDMS and LIG-Cu/PDMS. After three rounds of reuse, recycled LIG-Cu/PDMS still had potent cytotoxic effects on melanoma cells, suggesting its significant potential to reduce antineoplastic treatment costs.

**Figure 2:**
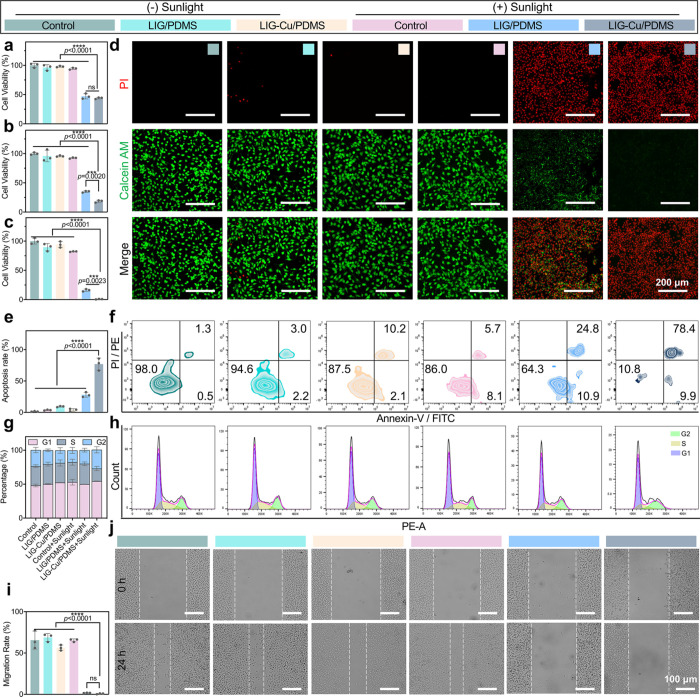
Antitumor effect of LIG-Cu/PDMS patches *in vitro*. a–c) Cell viability of B16–F10 cells against LIG/PDMS and LIG-Cu/PDMS in different stimulation durations with or without sunlight for 15 min (a), 30 min (b) and 60 min (c) (*n* = 3). d) Fluorescence images of B16–F10 cells with various treatments using Calcein AM and propidium iodide costaining, scale bar: 200 μm. e) Proportion of apoptotic B16–F10 cells (*n* = 3). f) Flow cytometry analysis of the apoptosis of B16–F10 cells induced by LIG/PDMS and LIG-Cu/PDMS after 60 min stimulation with or without sunlight (*n* = 3). g) Representative column graph analysis of B16–F10 cell cycle phases (*n* = 3). h) Flow cytometric analysis of cell-cycle phases after 60 min stimulation by LIG/PDMS and LIG-Cu/PDMS with or without sunlight for 60 min (*n* = 3). (i) Quantification of scratch assay results (*n* = 3). j) The respective images of scratch assay of B16–F10 cells subjected to sunlight stimulation at different time intervals using LIG/PDMS and LIG-Cu/PDMS. Scale bar: 100 μm. Data are presented as mean ± SD. Statistical significance between every two groups was calculated via one-way ANOVA. * *p* < 0.05, ** *p* < 0.01, *** *p* < 0.001, **** *p* < 0.0001; ns, not significant.

We further explored the underlying mechanism of cell death, specifically focusing on apoptosis, an important cell-death mechanism in antitumor studies.^37^ As shown in Figure 2e,f, and Figure S6, apoptosis analysis reveals that both LIG-Cu/PDMS and LIG/PDMS combined with sunlight significantly promote melanoma cell apoptosis. In the LIG/PDMS + Sunlight group, about 28% of cells undergo late apoptosis, while the percentage surges to over 70% for the LIG-Cu/PDMS + Sunlight group. Flow cytometry further confirms cell cycle arrest. Cancer cell proliferation critically depends on the DNA synthesis phase (S phase) of the cell cycle,^38^ and the proportion of cells in S phase is commonly utilized as an indicator of tumor proliferative status.^39^ As illustrated in Figure 2g,h, and Table S2, the LIG-Cu/PDMS phototherapy group exhibits the lowest percentage of cells in the S phase. During phototherapy, the LIG-Cu/PDMS patch disrupts normal DNA replication in cancer cells or arrests their progression at the G0/G1 checkpoint, leading to G1 phase arrest. This DNA damage accumulation inhibits cell repair mechanisms and promotes apoptosis, consistent with the results of the apoptosis assay. Cell migration is critical for cancer invasion and metastasis.^40^ Next, we investigate the inhibitory effect of LIG-Cu/PDMS patch on tumor migration using the scratch assay and transwell assay. Under sunlight, the LIG-Cu/PDMS group shows an extremely low migration speed (Figure 2i,j). Consistently, in the transwell assay, the migration activity of treated B16–F10 cells is inhibited by LIG/PDMS + Sunlight treatment (Figure S7). In conclusion, these results demonstrate that LIG-Cu/PDMS not only possesses remarkable direct tumor-killing capabilities but also inhibits tumor migration and metastasis.

### Therapeutic Performance of LIG-Cu/PDMS *In Vivo*

To validate the potent antitumor efficacy of LIG-Cu/PDMS observed *in vitro*, we established a subcutaneous B16–F10 melanoma model in C57BL/6J mice (Figure 3a). When the tumor volume reached approximately 80 mm^3^, we randomly divided tumor-bearing C57BL/6J mice into four groups (*n* = 3 per group): (i) control, (ii) LIG-Cu/PDMS, (iii) LIG/PDMS + Sunlight, and (iv) LIG-Cu/PDMS + Sunlight. We treated the mice on Day 1 and Day 5 after tumor formation and measured the tumor size and the mice’s weight over 10 days. Systemic therapeutic efficacy was confirmed through tumor growth analysis, demonstrating significant inhibition of malignant progression (Figure 3b–f). Representative photos depicting sequential treatment phases are presented in Figure 3g, enabling comparative analysis of morphological alterations across experimental groups. From Day 0 to Day 10, no significant weight loss occurred during tumor proliferation in all groups. Furthermore, mice in the LIG-Cu/PDMS + Sunlight group demonstrated the most rapid increase of body mass, which is correlated with an improved systemic condition (Figure 3b). We isolated the tumor tissue and recorded the tumor weight on Day 10. As shown in Figure 3h, the tumors in the LIG-Cu/PDMS + Sunlight group exhibit increased rigidity and more well-defined boundaries. These findings may be attributed to the selective ablation of tumor cells induced by phototherapy, which reduces tumor invasiveness and promotes the formation of fibrosis, thereby clarifying tumor boundaries. This suggests that the LIG-Cu/PDMS with sunlight treatment may be beneficial for subsequent therapeutic interventions like physical resection. Under simulated solar irradiation, LIG/PDMS and LIG-Cu/PDMS patches achieved steady-state temperatures of 50 °C within 5 min while maintaining localized tumor-bearing skin temperatures at 42 °C (Figure 3i,j and Figure S8), a physiologically safe threshold. This controlled thermal profile is attributed to efficient heat dissipation kinetics and the skin’s endogenous thermoregulatory mechanisms. Collectively, these data validate that LIG-Cu/PDMS-mediated PTT achieves tumor-specific hyperthermia (42 °C) while preserving systemic thermal homeostasis, aligning with the definition of mild hyperthermia in preclinical oncology.^17^ Following 10 days of post-treatment monitoring, melanomas treated with nonphotothermal LIG-Cu/PDMS patches exhibited comparable growth kinetics to untreated controls, whereas photothermal stimulation of LIG/Cu-PDMS patches induced significant tumor suppression, evidenced by progressive volume reduction over time. The LIG-Cu/PDMS with sunlight treatment efficiently and significantly reduces the tumor size, with an inhibition rate over 97% compared to the control group (Figure 3k). While all control mice succumbed to melanoma progression by Day 18, the LIG-Cu/PDMS + PTT group achieved an 80% survival rate at Day 30, demonstrating prolonged survival correlated with PTT-mediated antitumor efficacy (Figure 3l). High expression of Ki67 predicts poor prognosis in malignant oral melanoma patients.^41^ S100B, a calcium-binding protein in glial and melanocytes, is highly expressed in various cancers, especially melanoma.^42^ On Day 10, immunohistochemical analysis revealed marked downregulation of Ki67 and S100B in LIG-Cu/PDMS + PTT-treated tumors compared to controls (Figure 3m–p), correlating with suppressed tumor growth kinetics. In addition, the recurrence rate in the LIG-Cu/PDMS with the PTT group was monitored. Even when the observation period was extended to 60 days, no significant melanoma recurrence was observed in this treatment group (Figure S9). Collectively, these findings demonstrate that LIG-Cu/PDMS-based PTT represents an effective strategy for the treatment of melanoma *in vivo*, leading to a significant extension of mouse survival.

**Figure 3:**
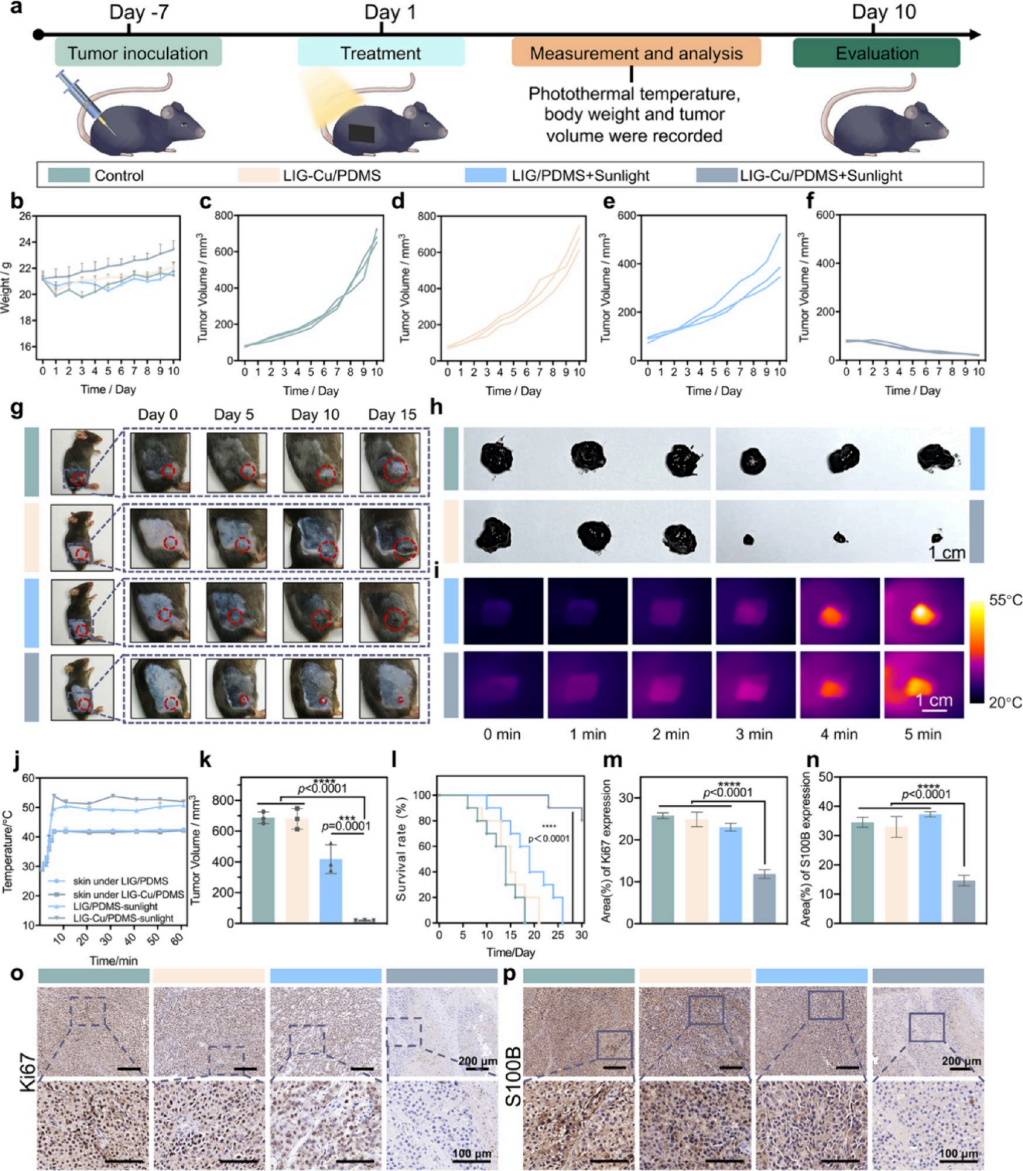
Therapeutic performance of LIG-Cu/PDMS patch *in vivo*. a) Schematic illustration of melanoma treatment *in vivo.* b) Changes in the body weight of C57BL/6J mice during different treatments (*n* = 3). c–f) Tumor volume change curves are depicted for the following conditions: the control group (c), LIG-Cu/PDMS without sunlight treatment (d), LIG/PDMS with sunlight treatment (e), and LIG-Cu/PDMS with sunlight treatment (f) (*n* = 3). g) Growth changes in tumor morphology. h) Anatomical images of tumors in each group on Day 10. Scale bar: 1 cm. (i) Temperature changes of the patch in the first 5 min. Scale bar: 1 cm. j) The temperature changes of the patch, as well as the tumor and skin beneath it, under illumination conditions (*n* = 3). k) Tumor volume of C57BL/6J mice in each group on Day 10 (*n* = 3). l) The survival rate of tumor-bearing mice within 30 days (*n* = 10). m–p) Expression levels of tumor markers Ki67 (m,o) and S100B (n,p) in different treatment groups (*n* = 3). Scale bars: 100 and 200 μm. Data are presented as mean ± SD. Statistical significance between every two groups was calculated via one-way ANOVA. * *p* < 0.05, ** *p* < 0.01, *** *p* < 0.001, **** *p* < 0.0001; ns, not significant.

### LIG-Cu/PDMS Phototherapy Modulates Cancer Pathways and Immune Responses via Gene Regulation

To gain deeper insight into how LIG-Cu/PDMS with light irradiation inhibits melanoma, gene transcription in tumor tissues from the control group and the LIG-Cu/PDMS phototherapy group was assessed (Figure 4a).^43^ Most gene expression levels were in the low-to-moderate range (0.01 to 100 FPKM) (Figures S10 and S11), indicating relatively uniform gene expression without extreme values. As depicted in the heatmap in Figure 4b, significantly differentially expressed genes (DEGs, genes with significant expression changes between experimental groups) are clearly annotated for intuitive visualization of expression variation. The Venn diagram analysis (Figure 4c) demonstrated 10,432 core genes maintained across treatment conditions, suggesting maintained baseline transcriptional homeostasis. High sample correlation implies similar gene-level characteristics (Figure S12). The MA-plot reveals 2570 upregulated and 121 downregulated genes in the LIG-Cu/PDMS + Sunlight group (Figure 4d and Figures S13 and 14). We focused on the following key genes (Figure S15). *Nectin1*, a tumor metastasis suppressor, is integral to cell–cell junctions and cytoskeletal reorganization, significantly influencing cell adhesion and migration.^44^ The low *Nectin1* expression can cause melanoma metastasis.^45^ In the LIG-Cu/PDMS + Sunlight group, *Nectin1* upregulation plays a critical role in enhancing antitumor ability. *Casp1* encodes Caspase-1, involved in inflammasome activation, apoptosis, and pyroptosis.^46^ Here, *Casp1* upregulation increases Caspase-1 activity, activating the inflammasome and producing inflammatory cytokines, which recruit immune cells.^47^ *Perp,* a p53-regulated protein, is related to apoptosis and cell junction.^48^ High expression of *perp* in LIG-Cu/PDMS + Sunlight group may enhance apoptosis sensitivity and reduce melanoma invasiveness.^49^ Additionally, *Gpx3 is* an antioxidant enzyme for hydrogen peroxide detoxification.^50^ In the LIG-Cu/PDMS + Sunlight group, high *Gpx3* expression may confer oxidative stress resistance and reduce tumor invasiveness.^51^ *Irgm1,* a GTPase for autophagy, is downregulated in the treatment group, possibly impairing autophagic flux and tumor cell viability.^52^ *MSX2*, a transcription factor that orchestrates cellular differentiation, proliferation, and apoptosis.^53^ Its upregulation can attenuate the stemness and chemoresistance of cancer cells by suppressing *SOX2*.^54^ Quantitative real-time PCR (qRT-PCR) was then employed to validate the genes identified in the transcriptome analysis. The primer sequences used are shown in Table S3. In the LIG-Cu/PDMS + Sunlight group, *Nectin1*, *Casp-1*, *Perp*, *Gpx3*, and *MSX2* RNA levels elevated to 72.43, 7.24, 1950.91, 14.43, and 655.53 times, respectively, while *Irgm1* decreased to 17.83% compared to the control group (Figure 4e). These results show the patch’s gene-modulating effect, consistent with antitumor experiments and transcriptome analysis, revealing its molecular-level antitumor mechanisms.

**Figure 4:**
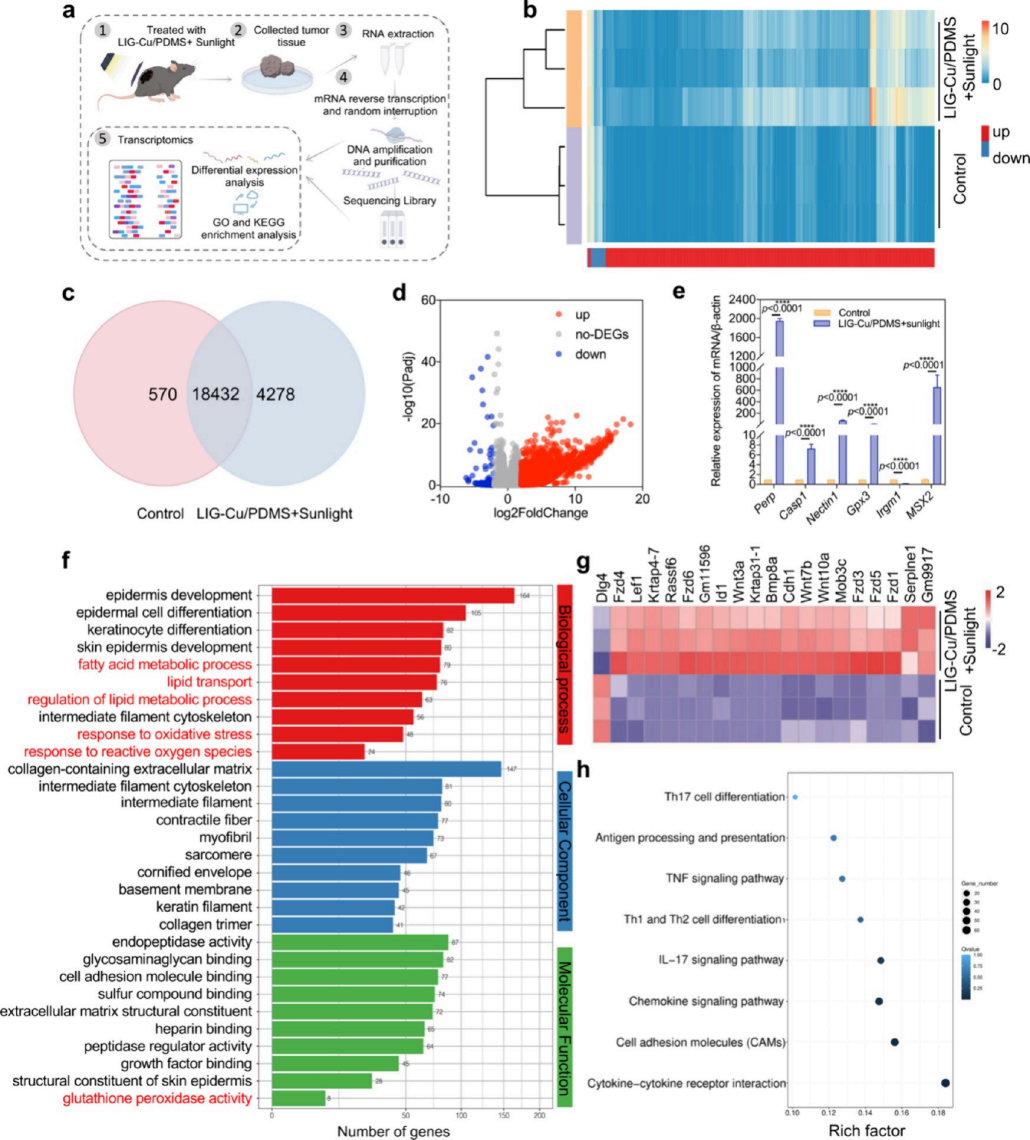
LIG-Cu/PDMS phototherapy modulates cancer pathways and immune responses via gene regulation. a) Transcriptomics schematic diagram. b) Heatmap of DEGs expression levels (*n* = 3). c) Intersample and intergroup Venn diagram analysis (*n* = 3). d) Volcano-plot distribution of DEGs (*n* = 3). e) qRT-PCR assay of relative expression of genes in melanoma tissues treated with various treatment modalities (*n* = 3). f) Pathway classification GO diagram of DEGs (*n* = 3). g) Clustering heatmap of DEGs in hippo signaling pathways (*n* = 3). h) The enrichment scatter plot to show KEGG enrichment analysis of a selection of immune-related KEGG terms (*n* = 3). Data are presented as mean ± SD * *p* < 0.05, ** *p* < 0.01, *** *p* < 0.001, **** *p* < 0.0001; ns, not significant.

To further elucidate the biological processes and signaling pathways associated with these DEGs, we performed Gene Ontology (GO) enrichment analysis. This standardized bioinformatic approach functionally annotates genes and identifies significantly enriched GO terms within three core ontologies: Biological Process, Cellular Component, and Molecular Function. Notably, the fatty acid metabolic process, lipid transport, regulation of lipid metabolic process, response to oxidative stress, response to ROS, and glutathione peroxidase activity attracted our attention. Changes in lipid metabolism and glutathione peroxidase activity are hallmarks of ferroptosis, an iron-dependent form of regulated cell death driven by excessive lipid peroxidation. In contrast, cuproptosis is a copper-dependent cell death pathway primarily characterized by the aggregation of lipoylated proteins in the tricarboxylic acid (TCA) cycle. Both pathways are closely associated with cellular redox homeostasis and ROS (Figure 4f). Furthermore, KEGG pathway enrichment analysis revealed that the cytochrome P450, PI3K-Akt signaling, and Hippo signaling pathways were significantly enriched, all of which have established links to the regulation of ferroptosis (Figure S16). Hippo is a key regulator in signal transduction pathways, which are associated with cell proliferation, survival, and differentiation (Figure 4g).^55^ Therefore, we further analyzed the DEGs of cuproptosis (Figure S17) and ferroptosis (Figure S18). The results indicated that LIG-Cu/PDMS-mediated phototherapy could significantly affect the regulatory genes related to cuproptosis and ferroptosis.

Based on the top 100 DEGs, a protein–protein interaction (PPI) network was constructed to reveal functional associations between their encoded proteins (Figure S19). Highly interconnected nodes such as Casp1, Casp14, perp, and Gpx3 correspond to proteins with multiple interactions, underscoring their key role in the regulatory pathways studied as potential regulatory or signaling centers. This interaction network reveals that the genes highlighted above and their interacting proteins may play a key role in eliminating melanoma under light exposure with the LIG-Cu/PDMS patch.

In addition, some immune-related pathways have also been enriched, which encourages us to further verify the immunotherapeutic effect of LIG-Cu/PDMS-mediated phototherapy in the animal experiments.^56^ Eight inflammation/immunity-related pathways from the scatter plot (Figure 4h) were classified into four major categories: immune signaling pathways, inflammatory responses, innate immune responses, and adaptive immune responses. In the LIG-Cu/PDMS + Sunlight group, cytokine-cytokine receptor interaction, cell adhesion molecules, and chemokine signaling pathways were significantly downregulated. These pathways are crucial for modulating the tumor microenvironment, regulating immune checkpoint pathways, recruiting immune cells, regulating immune responses, and enhancing antigen presentation, thereby amplifying the host’s antitumor immune response.^57^

### Antitumor Biological Mechanism of LIG-Cu/PDMS

The result suggested in the transcriptome analysis that LIG-Cu/PDMS-mediated phototherapy may cause ferroptosis, cuproptosis, and immunomodulation encourages us to continue verifying its antitumor biological mechanism. Divalent metal ions like Fe^2+^ and Cu^2+^ can generate ROS through the Fenton reaction under photothermal conditions. Excessive intracellular ROS triggers detrimental effects, leading to cell death forms such as apoptosis, ferroptosis, and cuproptosis. The downregulation of markers such as ferredoxin 1 (FDX1), lipoic acid synthetase (LIAS), and dihydrolipoamide S-acetyltransferase (DLAT) suggests the occurrence of cuproptosis. FDX1 is a key upstream regulator of protein lipoylation (mediated by LIAS). Excess intracellular copper is reduced to Cu^+^ by FDX1, and this Cu^+^ binds to lipoylated DLAT, triggering its toxic oligomerization/aggregation, which is a hallmark of cuproptosis, while reduced levels of Kelch-like ECH-associated protein 1 (KEAP1) and glutathione peroxidase 4 (GPX4) accompany ferroptosis. In addition, an imbalance in the tumor microenvironment, including changes in the levels of GSH, O_2_, and H_2_O_2_, further increases the susceptibility of cells to these forms of cell death (Figure 5a). These combined mechanisms contribute to the significant therapeutic effects observed in melanoma following efficient phototherapy with LIG-Cu/PDMS.^58^

**Figure 5:**
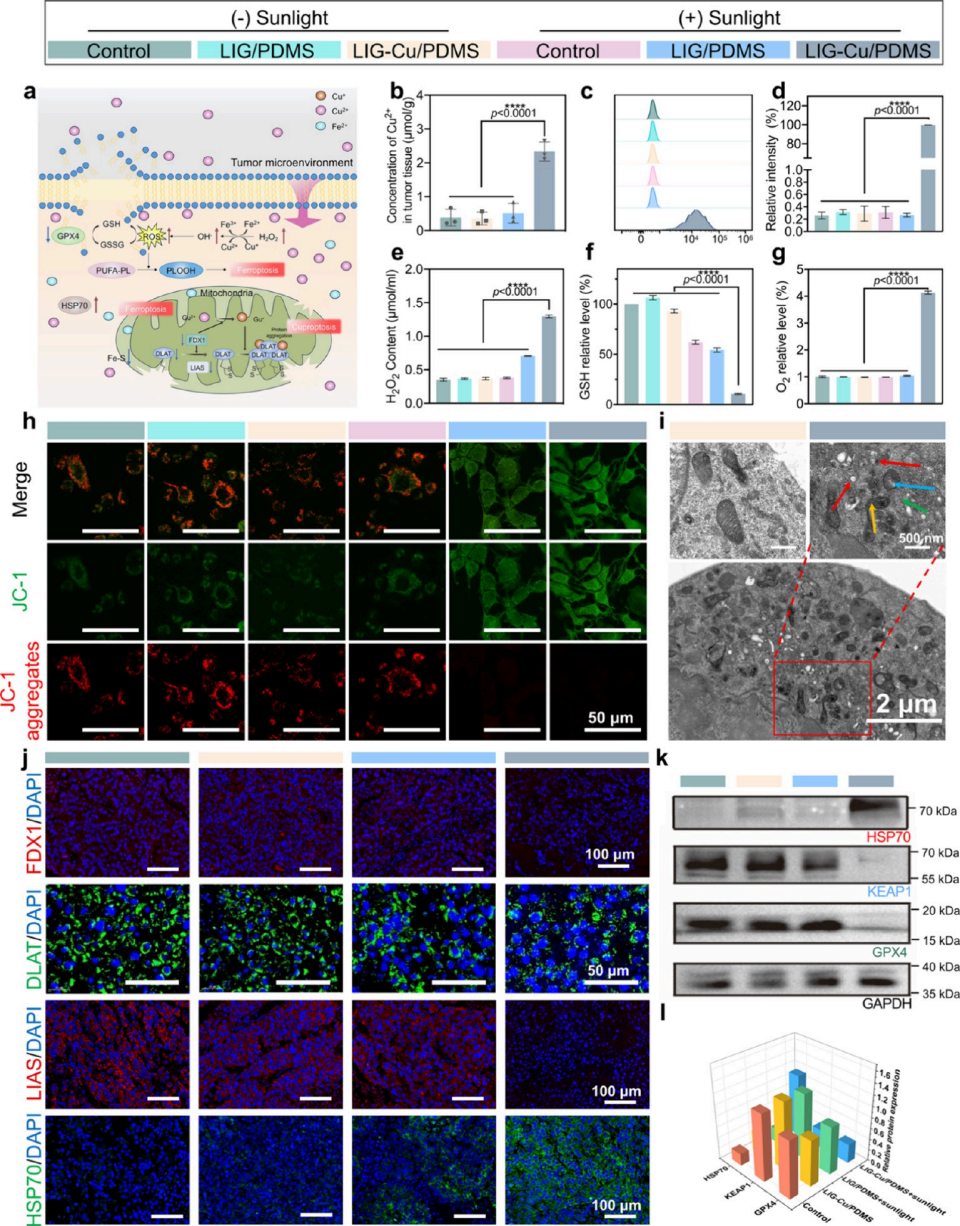
Biological mechanism of LIG-Cu/PDMS antitumor. a) The scheme of the biological mechanism of antitumor effect of LIG-Cu/PDMS patch phototherapy. b) Concentration of Cu^2+^ in melanoma tissues (*n* = 3). c) Flow cytometry assay of the intracellular ROS levels (*n* = 3). d) Quantitative analysis of flow cytometry of ROS in B16–F10 cells using the DCFH-DA fluorescent probe (*n* = 3). e) Content of H_2_O_2_ (μmol mL^–1^) in B16–F10 cells after different treatments (*n* = 3). f) Ratio of GSH in different groups (*n* = 3). g) Relative level of O_2_ in different groups (*n* = 3). h) Respective confocal fluorescence images of mitochondrial membrane potential detected using JC-1. Scale bar: 50 μm. (i) Respective transmission electron microscopy images of B16–F10 cells. Scale bar: 2 μm. Green arrow indicates the characteristic dense mitochondrial structure with remarkably reduced cristae and smaller volume; red arrows indicate autophagy of the endoplasmic reticulum, the yellow arrow shows autophagy of mitochondria, and the blue arrow indicates the pronounced swelling of the mitochondrial matrix. j) Respective immunofluorescence images of proteins related to cuproptosis. Scale bars: 50 and 100 μm. k,l) Western blot analysis of HSP70, GPX4, and KEAP1 expression (k) and their quantitative analysis (l) (*n* = 3). Data are presented as mean ± SD. Statistical significance between every two groups was calculated via one-way ANOVA. * *p* < 0.05, ** *p* < 0.01, *** *p* < 0.001, **** *p* < 0.0001; ns, not significant.

As previously demonstrated, LIG-Cu/PDMS-mediated phototherapy significantly promotes melanoma cell apoptosis (over 70% late apoptosis in the LIG-Cu/PDMS + Sunlight group; Figure 2e,f, Figure S6), induces G1 phase cell cycle arrest (Figure 2g,h), and inhibits tumor cell migration (scratch and transwell assays; Figure 2i,j, Figure S7). To further substantiate the causal link between these phenotypic changes and intrinsic apoptosis pathway activation, we performed molecular marker detection and pharmacological inhibition assays. Western blot analysis revealed marked downregulation of pro-Caspase-3 (the key effector caspase) and antiapoptotic Bcl-2 (a major guardian of mitochondrial membrane integrity) in treated cells (Figure S20).^59^ It is of note that the phototherapy-induced molecular changes were partially abrogated by pretreatment with Z-DEVD-FMK, a specific Caspase-3 inhibitor, thereby functionally implicating the activation of the apoptotic pathway as one of the mechanisms underlying the antitumor effect of LIG-Cu/PDMS-mediated phototherapy. In turn, this mechanistic validation substantially corroborates our initial phenotypic observations regarding apoptosis induction.

Since the LIG-Cu/PDMS patch with sunlight treatment showed superior *in vivo* antitumor efficacy to the LIG/PDMS group, the role of Cu^2+^ in the patch needs to be demonstrated. We measured the copper ion concentrations in tumor tissues of melanoma-bearing mice. The concentration in the LIG-Cu/PDMS + Sunlight group (2.33 μmol g^–1^) was significantly higher than in other groups (Figure 5b). PTT increased intracellular Cu^2+^ levels in tumor cells, indicating efficient Cu^2^^+^ delivery during LIG-Cu/PDMS + Sunlight treatment, which laid a critical foundation for subsequent investigations into the cuproptosis mechanism. Subsequently, we verified the ROS-sensitizing ability of LIG-Cu/PDMS with sunlight using the ROS probe DCFH-DA. As shown in Figures S21 and 22, cells treated with LIG/PDMS without sunlight or only with sunlight had weak fluorescence. In contrast, LIG-Cu/PDMS with sunlight led to bright green DCF emission, proving significant ROS generation. Flow cytometry also showed similar results (Figure 5c,d). An elevated concentration of hydrogen peroxide (H_2_O_2_), hypoxia, and excessive glutathione (GSH) are characteristics of the tumor microenvironment. The significantly elevated H_2_O_2_ levels observed in the LIG-Cu/PDMS group under sunlight irradiation (Figure 5e) provide abundant substrate for copper-catalyzed Fenton reactions, thereby enhancing ROS generation. GSH in tumor cells is the main endogenous antioxidant in the tumor microenvironment, acting as a copper chelator to protect tumor cells from cuproptosis.^60^ Cu^2+^ reacts with GSH to generate glutathione persulfide (GSSH), leading to a decline in ROS scavenging system efficiency and inducing cell death.^61^ We investigated the GSH/GSSG ratio as an indicator of the *in vivo* redox state.^62^ As illustrated in Figure 5f and Figure S23, LIG-Cu/PDMS + Sunlight treatments greatly decrease the GSH/GSSG ratio from 2.526 for the control to 1.496.

Tumor cells tolerate hypoxia mainly due to the overexpression of hypoxia-inducible factor 1 (HIF-1α), which activates downstream target genes such as VEGF, inducing angiogenesis in tumor cells and bringing oxygen and nutrients to promote tumor cell growth and proliferation.^63^ The transcriptomic results (Figure S24) show increased expression of genes that inhibit HIF-1α. Immunofluorescence results (Figures S25 and S26) show that LIG-Cu/PDMS under sunlight reduces HIF-1α expression, restricting the tumor adaptation to hypoxia and proliferation. We employ [Ru­(dpp)_3_]­Cl_2_, a fluorescent probe with oxygen-dependent fluorescence quenching,^64^ to estimate intracellular oxygen concentration. After LIG-Cu/PDMS patch phototherapy, cellular oxygenation levels increase over 4-fold compared to other groups (Figure 5g), significantly alleviating hypoxic tumors. These results indicate that the combination therapy of LIG-Cu/PDMS and sunlight effectively generates O_2_ and ROS while concurrently depleting excessive GSH. This synergy alleviates tumor hypoxia and disrupts the antioxidant defense barrier of tumor cells, thereby facilitating ROS-mediated lethal oxidative damage.

Mitochondrial membrane potential (MMP) alterations were assessed with the JC-1 assay kit. Red J-aggregates form in mitochondria with high MMP, while green J-monomers form in those with low MMP. LIG/PDMS + Sunlight and LIG-Cu/PDMS + Sunlight treatments of B16–F10 cells decrease the percentages of red signals from 80.59% (control) to 6.59% and 1.84%, respectively, indicating the severe mitochondrial damage (Figure 5h and Figure S27). In addition, a significant decrease in ATP levels was also observed (down to 44.8% of the control group), possibly due to copper-induced mitochondrial dysfunction inhibiting oxidative respiration (Figure S28).^65^ To further investigate the impact of the patches on mitochondrial structure, intracellular mitochondrial morphology was observed using transmission electron microscopy (TEM). As depicted in Figure 5i and Figure S29, the inner membrane cristae are orderly arranged, and the matrix appears homogeneous, without signs of vacuolation or damage in the control group. The mitochondria are of regular shape and size, indicating robust structural integrity and functionality. Similar characteristics are observed in the LIG/PDMS, LIG-Cu/PDMS, and control + Sunlight groups. However, in the LIG-Cu/PDMS + Sunlight group, we observed distinct mitochondrial damage indicative of cuproptosis. This included features such as pronounced swelling of the mitochondrial matrix, disorganization or disintegration of cristae, and rupture of the outer mitochondrial membrane (indicated by blue arrows), morphological alterations consistent with cuproptosis. After confirming cellular damage, key proteins in cuproptosis using immunofluorescence were evaluated. As a type of iron–sulfur (Fe–S) cluster protein, the upregulation of LIAS in the LIG/PDMS with the Sunlight group also implies the occurrence of cuproptosis. Additionally, the consumption of FDX1 in the light-treated LIG-Cu/PDMS group further confirmed the occurrence of cuproptosis. FDX1, serving as a substrate, simultaneously converts Cu^2+^ to the more toxic Cu^+^ and promotes the aggregation of DLAT, a hallmark of lipoylated protein aggregation. A significant aggregation of DLAT was also observed in the LIG-Cu/PDMS group under light exposure. Cu^+^ binds to DLAT, driving its oligomerization and subsequent cell death.^66^ LIG-Cu/PDMS under Sunlight exposure upregulates stress-induced heat shock proteins (HSP70) levels, leading to proteotoxic stress and ultimately resulting in cell death (Figure 5j and Figures S30–32).^67^ The Cu^2+^ chelator tetrathiomolybdate (TTM, 20 μM) was used to examine whether LIG-Cu/PDMS phototherapy mediates cuproptosis in tumor cells. Following TTM treatment, fluorescence signals of FDX1 and LIAS increased significantly, while DLAT oligomerization was markedly reduced compared to the LIG-Cu/PDMS-alone group, indicating that TTM alleviates LIG-Cu/PDMS-induced cuproptosis (Figure S33). These results further confirm that PTT mediated by LIG-Cu/PDMS orchestrates the occurrence of cuproptosis, which is strictly dependent on intracellular copper accumulation and lipoylated protein aggregation, the two core hallmarks of cuproptosis.

Ferroptosis has also been identified as a key cell death pathway induced by LIG-Cu/PDMS phototherapy. TEM morphology images (Figure 5i) show the characteristic dense mitochondrial structure with remarkably reduced cristae and smaller volume (green arrow), which are specific features of ferroptosis.^68^ Additionally, autophagy of the endoplasmic reticulum (red arrows) and mitochondria (yellow arrow) was also observed, suggesting a mixed cell-death process. To clarify the origin of these distinct phenotypes, we performed rescue experiments with pathway-specific inhibitors. In the presence of the copper chelator TTM, the swollen, cristae-disintegrated phenotype was suppressed, while the shrunken, dense ferroptotic morphology became unequivocally evident. Conversely, when ferroptosis was inhibited by Ferrostatin-1 (Fer-1), the swollen mitochondrial matrix and disintegrated cristae characteristic of cuproptosis were predominantly observed. These results confirm that both death pathways are independently activated and contribute to the overall mitochondrial damage. The BODIPY lipid ROS assay was employed to validate the occurrence of lipid peroxidation in cells. Upon lipid peroxidation, the BODIPY probe is oxidized by lipid hydroperoxides, resulting in a green shift in its excitation and emission spectra and a decrease in the red/green fluorescence ratio. As shown in Figure S34, the green fluorescence ratio in B16–F10 cells subjected to LIG-Cu/PDMS + Sunlight treatment significantly increased to 35.3%, compared to 0 in the control group, indicating robust lipid peroxidation. Although ferroptosis is primarily associated with dysregulated iron metabolism, Cu^2+^ can indirectly affect it through various mechanisms. ROS generated by the Fenton reaction promotes lipid peroxidation.^69^ Cuproptosis further impairs mitochondrial structure and Fe–S cluster proteins, releasing free Fe^2+^ and increasing oxidative stress. These processes create a self-reinforcing cycle of ferroptosis and cuproptosis, ultimately achieving a potent antitumor effect.^70^ GSH, a cofactor of GPX4, which is a crucial enzyme for inhibiting lipid peroxidation, promotes ferroptosis by reducing GPX4 activity upon its depletion.^71^ The NRF2-KEAP1 pathway suppresses ferroptosis by negatively regulating KEAP1,^72^ a key inhibitor of NRF2 activity.^73^ To confirm if ferroptosis occurred in LIG-Cu/PDMS + Sunlight-induced melanoma cell death, we examined the expression of ferroptosis-related proteins GPX4 and KEAP1. Western blot analysis revealed that both GPX4 and KEAP1 were significantly downregulated in the LIG-Cu/PDMS + Sunlight group compared to other groups (Figure 5k–l), proving that this combinatorial therapy triggers ferroptosis. The ferroptosis inhibitor Fer-1 was used to verify the occurrence of ferroptosis. As shown in Figure S35, compared to the control group, cells treated with LIG-Cu/PDMS phototherapy exhibited a significant decrease in red fluorescence intensity and a marked increase in green fluorescence, indicating substantial lipid peroxidation. In contrast, in the group cotreated with Fer-1 and LIG-Cu/PDMS phototherapy, red fluorescence intensity was notably restored compared to the LIG-Cu/PDMS-alone group. Western blot analysis yielded consistent results; Fer-1 treatment significantly rescued the low expression of GPX4 and KEAP1 observed in the LIG-Cu/PDMS phototherapy-only group (Figure S36). Together, these experiments demonstrate that LIG-Cu/PDMS phototherapy indeed activates ferroptosis-related pathways in tumor cells. Notably, we have systematically decoupled and integrated the individual evidence chains supporting cuproptosis and ferroptosis activation via multilayered experimental validations, including transcriptomic pathway analysis, transmission electron microscopy observation of mitochondrial lesions, immunofluorescence detection of core pathway proteins, copper chelator and ferroptosis inhibitor rescue assays, and lipid peroxidation evaluation. These experiments distinctly delineate the independent molecular hallmarks of each cell death pathway and clarify their synergistic crosstalk, further consolidating the mechanistic rigor of the synergistic cell death induction by our LIG-Cu/PDMS phototherapeutic platform. In summary, under sunlight irradiation, the excellent antitumor effect might be due to photothermally triggered Cu^2+^ release by LIG-Cu/PDMS, enhancing the synergistic cuproptosis/ferroptosis/apoptosis/photothermal therapeutic effect.

### LIG-Cu/PDMS Promotes Tumor Microenvironment Remodeling

Abundant evidence has shown that PTT can cause a cascade immunogenic cell death (ICD) effect. We detected key DAMPs. Immunofluorescence staining of tumor tissues revealed pronounced cytoplasmic translocation of High Mobility Group Box 1 (HMGB1) and surface exposure of Calreticulin (CRT) in the LIG-Cu/PDMS + Sunlight group compared to controls (Figures S37 and S38). Additionally, an *in vitro* assay confirmed that the treatment triggered a significant increase in extracellular ATP release from melanoma cells (Figure S39). The ectopic localization of HMGB1 and CRT, along with ATP secretion, are established hallmark of ICD and provide the mechanistic “find-me” and “eat-me” signals necessary for robust immune activation.^74^ We continued to explore the impact of the LIG-Cu/PDMS patch on the PTT immunotherapy of melanoma. Dendritic cells (DCs), as pivotal antigen-presenting cells (APCs), orchestrate antitumor immunity by capturing tumor-associated antigens, upregulating costimulatory molecules (e.g., CD80/CD86), migrating to lymph nodes, and activating CD8^+^ T cells through cross-presentation, thereby initiating robust antitumor responses. We assessed the effects of phototreated B16–F10 cell lysates derived from different LIG patch variants on bone marrow-derived dendritic cells (BMDCs) in *vitro* (Figure 6a). The results indicate that LIG-Cu/PDMS with sunlight has a strong capability to promote the maturation of DCs. The maturation of BMDCs in the LIG-Cu/PDMS + Sunlight group reaches 37.17 ± 2.65%, which is better than the LIG/PDMS + Sunlight group (25.07 ± 1.57%), and significantly higher than other groups (Figure 6b). The proportion of CD80^+^ in CD11c^+^ DCs and CD86^+^ in CD11c^+^ DCs significantly increases after phototherapy with LIG-Cu/PDMS patch in Figure 6c–f.

**Figure 6:**
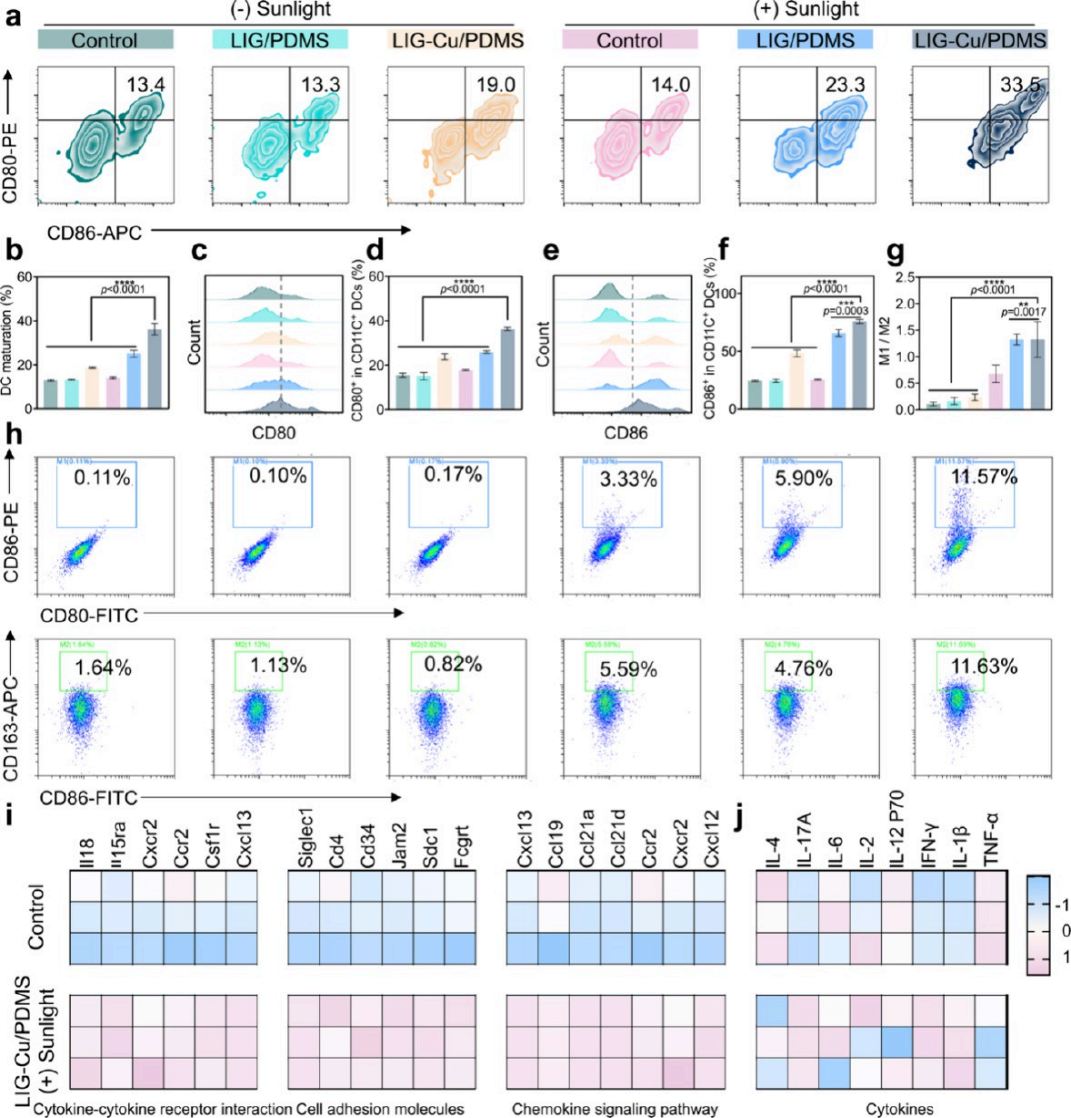
LIG-Cu/PDMS promotes immune reprogramming. a–f) Flow cytometry analysis of CD80^+^ and CD86^+^ expression (a) BMDCs maturation (b), the histogram plots (c) and quantitative analysis (d) of CD80 and the histogram plots (e) and quantitative analysis (f) of CD86 after incubation of BMDCs with supernatants from B16–F10 cells after different treatments (*n* = 3). g) The ratio of M1 to M2 macrophages. (*n* = 3). h) Flow cytometry assay of the M1-like Thp-1 (CD80^+^CD86^+^), and M2-like Thp-1 (CD86^+^CD163^+^) under different treatment (*n* = 3). (i) Heat map of DEGs in cytokine-cytokine receptor interaction, cell adhesion molecules, lysosome, and chemokine signaling pathway (*n* = 3). j) Heat map of eight cytokines in serum (*n* = 3). Data are presented as mean ± SD * *p* < 0.05, ** *p* < 0.01, *** *p* < 0.001, **** *p* < 0.0001; ns, not significant.

Tumor-associated macrophages (TAMs) are essential for tissue repair and shaping the tumor microenvironment,^75^ and the polarization direction of macrophages is closely linked to the progression of tumors. Copper ion carrier drugs have been reported to induce tumor cell death while polarizing macrophages into the M1 phenotype, thereby enhancing the antitumor immune response.^76^ M1-type macrophages activate T cells, utilize antitumor effector functions (including phagocytosis or cytokine-mediated cell death) in the tumor microenvironment, and are critical for effective tumor control.^77^ M2-type macrophages are the anti-inflammatory part of the immune system, and they typically aggregate in the tumor microenvironment, promoting tumor growth and metastasis.^78^ To investigate whether the antitumor activity of the LIG-Cu/PDMS patch is also correlated with polarization of the macrophage phenotype, we utilized flow cytometry assay to detect the expression of the M1 marker (CD80 and CD86) and the M2 markers (CD163) in M0 phenotype of THP-1 after different treatments. The significant upregulation of CD80 and CD86 expression in M1 macrophages indicates that these cells are in a pro-inflammatory state, ready to interact with T cells.^79^ LIG-Cu/PDMS phototherapy significantly enhances the polarization of THP-1 from M0 to M1 under phototherapy and substantially increases the M1/M2 ratio, remodeling the tumor microenvironment and activating the immune response. An increased M1/M2 ratio promotes an immunosupportive tumor microenvironment and enhances tumor eradication (Figure 6g,h and Figure S40). Furthermore, we measured the expression of IL-1β in THP-1 cells, a cytokine typically overexpressed in M1-polarized macrophages (Figure S41). The results showed that IL-1β levels in the LIG-Cu/PDMS phototherapy group were significantly higher than those in all other experimental groups, indicating that the treatment significantly promoted the polarization of THP-1 cells from the M0 to the M1 phenotype. More relevant gene set enrichment analysis (GSEA) evidence was also found. Analysis utilizing the KEGG database has demonstrated that the LIG-Cu/PDMS + Sunlight group induces a robust upregulation of genes associated with multiple tumor immunity-related pathways, including cytokine-cytokine receptor interaction, cell adhesion molecules, and chemokine signaling pathway (Figure 6i). For instance, CXCL13 indirectly strengthens the antitumor immune landscape within the tumor microenvironment by promoting DCs and CD4^+^ T cell activity.^80^ Furthermore, CXCR2 and CCR2 primarily facilitate the chemotactic migration of M1 macrophages and other immune cells, enhancing immune cell infiltration into tumor sites.^81^

The expression profiles of immune factors in murine serum further validated the remodeling of the tumor immune microenvironment. The expression levels of IL-4, IL-17A, IL-6, IL-2, IL-12P70, IFN-γ, IL-1β, and TNF-α in the serum of mice in the LIG-Cu/PDMS + Sunlight group and the control groups were detected (Figure 6j). In the LIG-Cu/PDMS patch phototherapy group, the expression of IL-17A, IFN -γ, and IL-1β is significantly upregulated, while that of IL-4 and TNF-α are downregulated, with no significant differences in IL-6, IL-2, and IL-12p70. The overexpression of IL-17A in the LIG-Cu/PDMS + Sunlight group may promote melanoma immunosuppression by enhancing the recruitment of CD4^+^ Th17 cells, cytotoxic T cells, and DCs, consistent with previous studies reporting elevated CD4^+^ T cell and DC populations in tumor-immune microenvironments. High levels of infiltrating IFN-γ signals reflect extensive tumor inflammation, which can improve melanoma patients’ responses to checkpoint immunotherapy.^82^ IL-1β overexpression in this group can exert antitumor effects and cooperate with IL-2 and IFN-γ to induce cytotoxic CD8^+^ T lymphocytes and Natural killer cells’ killing activity, as it promotes Th1 cell differentiation and enhances the immune cells cytotoxicity.^83^ IL-4 promotes the formation of a tumor-inflammatory microenvironment, stimulates angiogenesis, and enhances tumor cell migration. Thus, the downregulation of IL-4 by LIG-Cu/PDMS + Sunlight inhibits tumor progression.^84^

Tumor-infiltrating immune cells were further analyzed to investigate the mechanisms underlying antitumor immune responses. The LIG-Cu/PDMS + Sunlight group exhibits the most intense signals for tumor-infiltrating CD3^+^, CD3^+^ CD4^+^, and CD3^+^ CD8^+^ T cells, with significant increases in quantities to 3.60% ± 0.04%, 1.50% ± 0.09%, and 1.12% ± 0.06%, respectively (Figure 7a–d).We further analyzed the spleens of all experimental groups for the following T-cell subpopulations: IFN-γ^+^ CD8^+^ CD3^+^cells, PD-1^+^ CD8^+^ CD3^+^cells, CD44^+^ CD62L^–^ CD4^+^ CD3^+^ cells, and CD44^+^ CD62L^–^ CD8^+^ CD3^+^ cells, which represent tumor-specific cytotoxic CD8^+^ T cells, CD8^+^ T-cell checkpoint expression, and effector memory T cells (Figure 7e,f), respectively. Cytotoxic CD8^+^ T lymphocytes play a pivotal role in providing effective antigen-specific immunity against tumors.^85^ The LIG-Cu/PDMS + Sunlight group showed a significant increase in IFN-γ^+^ CD8^+^ CD3^+^ T cells compared to the control (1.50-fold) and LIG-Cu/PDMS-alone (1.30-fold) groups (Figure S42a). The proportion of PD-1^+^ CD3^+^ CD8^+^ cells showed no significant difference among all the treatment groups and the control group, indicating that our LIG-Cu/PDMS phototherapy treatment did not significantly increase CD8^+^ T-cell exhaustion (Figure S42b). Furthermore, the percentage of CD4^+^ effector memory cells in the spleens of the control group remained low, ranging from 26.04% to 27.83%. In contrast, the LIG-Cu/PDMS + Sunlight group exhibited a significant increase in the effector memory T cells proportion, reaching 37.03%, which is approximately 1.38-fold higher than that in the control group. The proportion of CD8^+^ effector memory cells in the LIG-Cu/PDMS + Sunlight group was also significantly elevated compared to other groups, at approximately 29.26% (Figure 7e,f). This indicates a substantial enhancement in the efficacy and longevity of cytotoxic T lymphocytes (CTLs) and T helper cells. In summary, LIG-Cu/PDMS phototherapy effectively activates T-cell-mediated antitumor immunity and promotes the development of antitumor immune memory.

**Figure 7:**
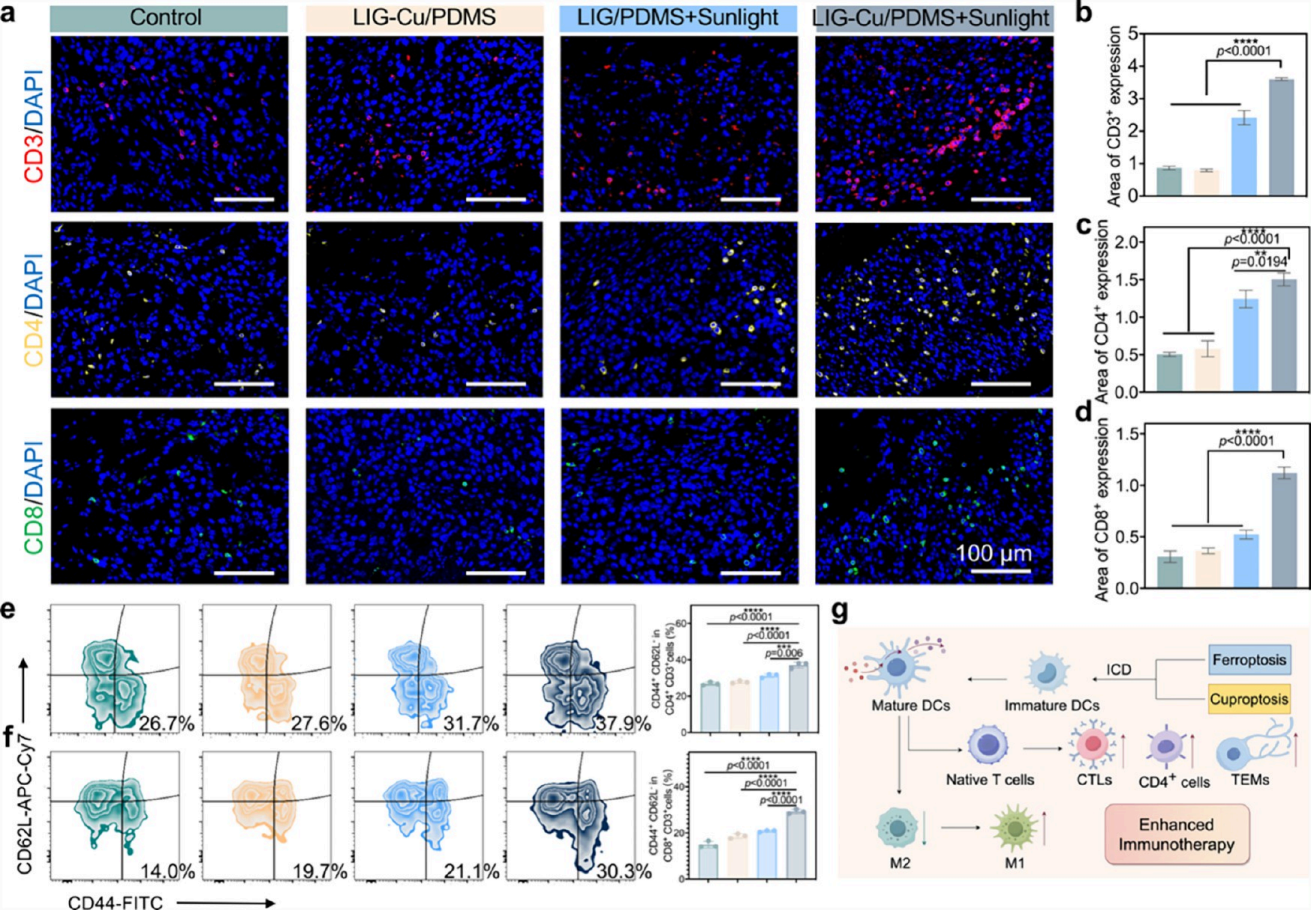
LIG-Cu/PDMS promotes immune reprogramming *in vivo*. a) Immunofluorescence images of CD3^+^, CD4^+^, CD8^+^ cells in tumor region. Scale bar: 100 μm. b–d) Relative expression level of CD3^+^ cells (b), CD4^+^ cells (c) and CD8^+^ cells (d) (*n* = 3). e,f) Representative flow cytometry images and quantification of CD44^+^ CD62L^–^ CD4^+^ cells (e) and CD44^+^ CD62L^–^ CD8^+^ cells (f) under different treatments in the spleen region (*n* = 3). g) The scheme of the enhanced immunotherapy mechanism of LIG-Cu/PDMS patch phototherapy. Data are presented as mean ± SD * *p* < 0.05, ** *p* < 0.01, *** *p* < 0.001, **** *p* < 0.0001; ns, not significant.

Ultimately, the direct evidence of ICD (HMGB1/CRT translocation and ATP release) mechanistically links the mitochondrial damage-induced tumor cell death to the observed immune activation. DAMPs are released after tumor cell death, which promotes the transformation of TAMs from a nonactivated M0 phenotype to an immunostimulatory M1 phenotype, induces DCs maturation and promotes T-cell infiltration. As a result, an antitumor immune response is triggered *in vivo*, reprogramming the immunosuppressive tumor microenvironment (Figure 7g).^86^

### Biosafety Evaluation of LIG-Cu/PDMS

LIG-Cu/PDMS and LIG/PDMS exhibit no significant cytotoxicity toward normal fibroblasts MRC-5 (Figure S43), indicating that LIG-Cu/PDMS patches do not damage normal tissue. Meanwhile, LIG-Cu/PDMS has markedly higher cytotoxicity in melanoma cells than in fibroblasts under the same conditions (Figure S44), making them suitable for potential skin tumor therapy. Having demonstrated the low cytotoxicity of LIG-Cu/PDMS toward normal cells *in vitro*, we then investigated its *in vivo* biocompatibility for potential clinical application (Figure 8a). In the 2-day post-treatment period, there is no significant difference in body weight among all groups (Figure 8b,c). The treated skin areas in the mice showed new hair growth. Cu^2+^ accumulation in other organs (liver, kidney, spleen, and serum) has also been detected in Figure 8d and Figure S45, which showed that there was no significant difference in Cu^2+^ content in various organs between the LIG-Cu/PDMS phototherapy group and the control group, confirming that this phototherapy has no risk of systemic copper accumulation. Hematoxylin and Eosin (H&E) staining of major organs (heart, liver, spleen, lungs, and kidneys) from each group shows no pathological abnormalities (Figure S46). Specifically, the myocardial cells are neatly arranged, with no necrosis or inflammatory cell infiltration; the hepatic lobule structure is clear, with no fatty degeneration or necrosis of hepatocytes; the white and red pulp structures of the spleen are normal; the lung tissue shows no interstitial edema or inflammatory cell infiltration; and the glomeruli and renal tubules are intact with no pathological changes. Fifteen days after the phototherapy, blood samples were collected for biochemical analysis. Hematology and serum biochemistry results for all groups are within normal ranges (alanine aminotransferase, ALT; aspartate aminotransferase, AST; alkaline phosphatase, ALP; total cholesterol, TC; lactate dehydrogenase, LDH; Figure 8e–h and Table S4). Additionally, the skin stratum corneum in the three treatment groups exhibited a similar architecture to that of the control. It displays a consistent band-like distribution on the active epidermis, which is tightly connected to the epidermis without any stratum corneum peeling or separation (Figure 8i). This indicates that the novel patch treatment does not leave permanent skin damage or scarring. We also assessed the long-term biosafety of LIG-Cu/PDMS patches in healthy C57BL/6J mice. The same treatment methods were applied to healthy C57BL/6J mice. No significant weight loss occurred in LIG-Cu/PDMS patch phototherapy treatment group, indicating no systemic toxicity (Figure S47). H&E staining of major organs (heart, liver, spleen, lungs, kidneys and skin) showed no pathological changes, such as necrosis, fibrosis, or inflammatory infiltrates (Figure S48). Complete blood count (CBC) showed all parameters (e.g., white blood cell, WBC; red blood cell, RBC; platelets) within normal ranges, suggesting no hematological toxicity (Figure S49). Serum biochemistry revealed no abnormalities, confirming the absence of hepatorenal damage (Figure S50). These results demonstrate the long-term safety of our LIG-Cu/PDMS phototherapy. In conclusion, the LIG-Cu/PDMS patch exhibits excellent biocompatibility. As evidenced by our findings, this promising biosafety profile could be translated into substantial clinical potential after more in-depth assessments, including good laboratory practice toxicology studies.

**Figure 8:**
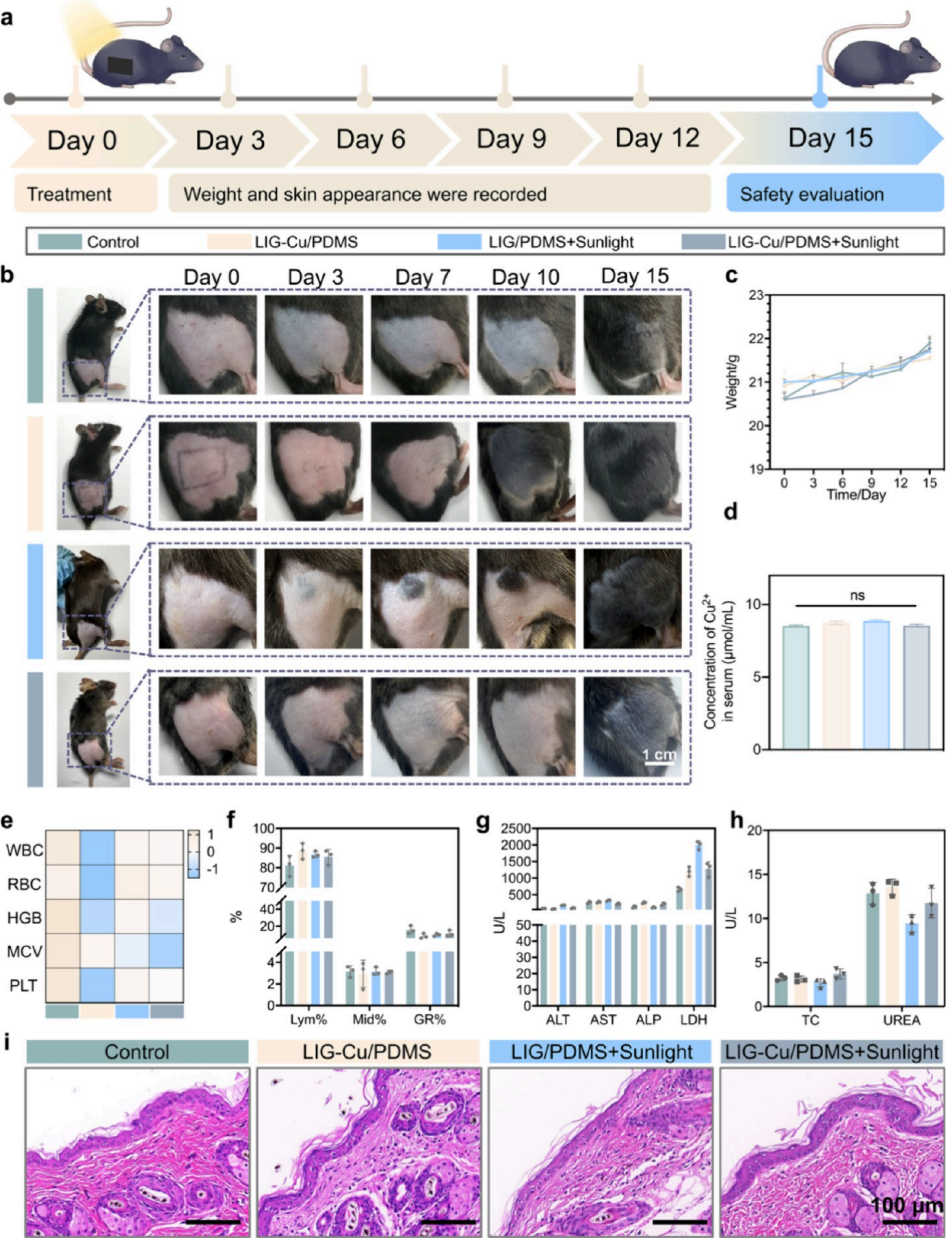
Biosafety evaluation of LIG-Cu/PDMS. a) Schematic illustration of biosafety evaluation *in vivo.* b,c) Local skin manifestations (b) and weight variations (c) in healthy C57BL/6J mice under various treatment regimens (*n* = 3). Scale bar: 1 cm. d) Cu^2+^ accumulation in serum. e) Heatmap of hematologic mean values analysis for each group of mice on Day 15 (*n* = 3). f–h) Hematology and serum biochemistry mean values for each group of mice on Day 15 (*n* = 3). (i) H&E staining of treated skin regions. Scale bar: 100 μm. Data are presented as mean ± SD * *p* < 0.05, ** *p* < 0.01, *** *p* < 0.001, **** *p* < 0.0001; ns, not significant.

## Conclusion

In this study, we engineered a flexible, transparent, and reusable LIG-Cu/PDMS patch for targeted melanoma therapy, where CuO-embedded LIG serves as the core therapeutic component and PDMS provides a biocompatible, conformable matrix.^87^ This noninvasive, easily fabricated platform integrates mild photothermal therapy (≤42 °C) with the synergistic activation of cuproptosis, ferroptosis, and apoptosis, achieving dual effects of direct tumor elimination and tumor immune microenvironmentremodeling. Mechanistically, mild photothermal stimulation triggers site-specific release of Cu^2+^ from the patch, which accumulate preferentially in tumor tissues rather than major organs, thus avoiding systemic copper retention and long-term toxic risks.^88^ Intracellular copper accumulation induces lipoylated protein aggregation and mitochondrial dysfunction by directly binding to lipoylated components of the TCA cycle to initiate canonical cuproptosis,^65^ while mitochondrial damage further promotes ROS burst and lipid peroxidation, amplifying ferroptosis.^89^ The collaborative action of these pathways not only eradicates melanoma cells efficiently, but also releases tumor-associated antigens and DAMPs, activating systemic antitumor immunity, preventing tumor metastasis.^90^ Notably, the patch’s reusability and noninvasive administration further reduce clinical application barriers, overcoming the limitations of surgical risks and chemoradiotherapy resistance in conventional melanoma treatment. Central to the reliability of this therapeutic strategy is the strictly standardized mild photothermal regimen, implemented via a simulated solar irradiation system that confers precise controllability and excellent reproducibility, thus forging a dependable basis for future clinical validation. Collectively, this work establishes a novel paradigm for LIG-based biomedical materials in cancer therapy, highlighting the great translational potential of multifunctional patches in targeted, safe, and efficient tumor treatment and expanding the application landscape of graphene-derived materials in biomedicine.

## Materials and Methods

### Cell Viability Assay

Cell viability was determined using the Cell Counting Kit-8 (CCK-8) (GlpBio, Jinan, China) method. Briefly, B16–F10 and MRC-5 cells were uniformly seeded in equal amounts (1.5 × 10^6^ well^–1^) on circular cell coverslips with a diameter of 9 mm. After different treatment measures (groups with sunlight treatment were exposed to simulated Xenon sunlight at an intensity of 1.5 kW m^–2^ for 60 min), the supernatant was removed, and 110 μL of CCK-8 working solution (incomplete medium mixed with CCK-8 reagent in a 10:1 ratio) was added (1.5 kW m^–2^ = 1.5 sunlight). The samples were then incubated at 37 °C for 1–2 h. Finally, absorbance at 450 nm was measured with a microplate reader (PerkinElmer, MA, USA).

### Scratch Assay

B16–F10 cells in suspension in the logarithmic growth phase were seeded into a 24-well plate, ensuring that the cells reach 100% confluence after overnight incubation. Using a 200 μL pipet tip, evenly spaced scratches perpendicular to the surface of the 24-well plate were created, and the initial scratch width was recorded. The scratch area was washed with PBS buffer to remove any cell debris generated by the scratching. A serum-free medium was added, and after 24 h of treatment under different interventions, cell migration within the scratched area was observed and recorded. The cell migration speed and capability were analyzed based on the collected image data.

### Reactive Oxygen Species (ROS) Detection

B16–F10 Cells from different groups were incubated with DCFH-DA (10 μM) (Beyotime Biotechnology, Shanghai, China, Cat#S0033S) at 37 °C for 30 min. The fluorescence of different treated cells was imaged by CLSM (Nikon, Shanghai, China) and analyzed by flow cytometry (CytoFlex, Beckman Coulter, Indiana, USA).

### Measurement of H_2_O_2_

To determine the intracellular H_2_O_2_ content, we employed the H_2_O_2_ Content Assay Kit (Solarbio, Beijing, China, BC3595). Initially, B16–F10 cells were seeded in 6-well plates at a density of 1 × 10^5^ cells per well and incubated for 24 h. Subsequently, the cells were treated with LIG/PDMS and LIG-Cu/PDMS. For the light treatment, the cells were exposed to simulated Xenon sunlight at an intensity of 1.5 kW m^–2^ for 60 min, whereas the dark control group was kept in the dark. Following these treatments, cell precipitates were promptly collected and analyzed for H_2_O_2_ content using the assay kit.

### Measurement of Relative Oxygen Content

Dilute the [Ru­(dpp)_3_]­Cl_2_ probe (BIOESN, Shanghai, China, Cat#BES20583BO) in serum-containing culture medium at a 1:100 ratio. Carefully remove the culture medium from both the control and experimental groups and wash the cells once with PBS. Add 200 μL of the diluted [Ru­(dpp)_3_]­Cl_2_ probe to each well. Incubate the cells in the dark at 37 °C for 60 min. After incubation, measure the absorbance at 455 nm using a microplate reader (PerkinElmer, MA, USA).

### Western Blotting

The membranes containing the target protein were incubated overnight at 4 °C with primary antibodies for HSP70 (rat, 1:2000, ABclonal, Wuhan, China, Cat#A1507), GAPDH (rat, 1:5000, HuaBio, Hangzhou, China, Cat#ET1601–4), KEAP1 (rat, 1:1000, ABclonal, Wuhan, China, Cat#A17062), and GPX4 (rat, 1:1000, ABclonal, Wuhan, China, Cat#A11243), ensuring precise detection of the proteins of interest. Following this, the membranes were incubated with Anti-Rabbit IgG conjugated to HRP (rat, 1:100000, HuaBio, Hangzhou, China, Cat#HA1001) Anti-Mouse IgG conjugated to HRP (rat, 1:100000, HuaBio, Hangzhou, China, Cat#HA1008) for 1 h at room temperature. The results were visualized using an enhanced chemiluminescence detection system (Bio-Rad, Hercules, CA, USA).

### *In Vitro* Promotion of BMDCs Maturation

B16–F10 cells were seeded in 24-well plates and coincubated with PBS, LIG/PDMS, and LIG-Cu/PDMS, followed by exposure or nonexposure to sunlight. Cell supernatants were collected separately. Mouse BMDCs were seeded in 24-well plates and incubated with the collected culture supernatants for 24 h. Subsequently, BMDCs were costained with anti-Mouse CD80-PE (1:500, Elabscience, Wuhan, China, E-AB-F0992D), anti-Mouse CD86-APC (1:500, Elabscience, Wuhan, China, E-AB-F0994E), and anti-Mouse/Human CD11c-FITC (1:500, Elabscience, Wuhan, China, E-AB-F0994E) antibodies and analyzed by flow cytometry.

### Macrophage Polarization

The differentiation of human monocyte THP-1 cells into M0-type macrophages was induced by treating them with Phorbol 12-myristate 13-acetate (PMA) at a concentration of 100 ng mL^–1^ for 48 h. Cell suspensions from each group were incubated with the relevant antibodies (CD80, Liankebio, Hangzhou, China, Cat#F1108001; CD86, Liankebio, Hangzhou, China, Cat#F1108602; CD163, Liankebio, Hangzhou, China, Cat#F1116303) for 30 min. The results were analyzed by flow cytometry (CytoFlex, Beckman Coulter, Indiana, USA).

### *In Vivo* Antitumor Effect

All animal procedures were approved by the Animal Ethics Committee of Wuhan University (No. WP20220020). To investigate the antitumor effects of LIG/PDMS and LIG-Cu/PDMS *in vivo*, 2 × 10^5^ B16–F10 cells were subcutaneously injected into the right femoral region of C57BL/6 mice. One week later, the tumor-bearing C57BL/6 mice with tumor volumes of 60–80 mm^3^ were randomly divided into four groups (*n* = 3): (a) untreated group; (b) LIG-Cu/PDMS group; (c) LIG/PDMS + Sunlight group; (d) LIG-Cu/PDMS + Sunlight group. In groups (b), (c), and (d), patches measuring 1 cm × 1 cm were applied to the mice’s skin. Then, mice in groups (c) and (d) were exposed to simulated Xenon sunlight at an intensity of 1.5 kW m^–2^ for 60 min, whereas the mice in group (c) were placed in darkness. The thermal infrared camera system (MobIR Air, Guide Sensmart, China) was used to record temperature changes and photothermal images every 10 s. Treatments were conducted on Day 1 and Day 5, and the mice’s body weight and tumor volumes (calculated as V = 0.5 × length × width^2^) were monitored every 2 days. On Day 10, the mice were euthanized, and the tumors and main organs were collected for further analysis.

### Determination of Cu^2+^ Concentration within Tissues

Cu^2+^ concentration in tumors was measured 24 h postphototherapy in both the control group and the experimental groups of mice using a copper ion detection assay kit (Boxbio, Beijing, China, Cat#AKIC005M) and a microplate reader (PerkinElmer, MA, USA).

### Immunofluorescence Staining

The experimental steps and principles of immunofluorescence are similar to those of immunohistochemistry, which is also realized by the highly specific binding between primary antibodies-HSP70 (rabbit, 1:200, Abways, Shanghai, China, Cat#CY5496), LIAS (rabbit, 1:200, Abcam, Cambridge, UK, Cat#ab246917), DLAT (rabbit, 1:100, Abways, Shanghai, China, Cat#CY8125), FDX1 (rabbit 1:200, Abways, Shanghai, China, Cat#DY1671) and secondary antibody, Goat Anti-Rabbit IgG (Cy3) (1:200, Pinuofei, Wuhan, China, Cat#PN0046). Antibodies of different indicators are labeled with fluorescein, and the content and distribution of HSP70, LIAS, DLAT, and FDX1 are observed under a fluorescence microscope.

### Immunohistochemistry Assay

Immunohistochemistry assay was performed by paraffin-embedded tumor. Sections are obtained. According to the principle of antigen–antibody reaction and chemical color development, antigens in the tissue sections are first bound to the primary antibodies-S100B (rat, 1:100, Abways, Shanghai, China, Cat#CY5201) and Ki67 (rat, 1:500, Abcam, Cambridge, UK, Cat#ab16667), and then reacted with Goat Anti-Rabbit IgG (HRP) (rat, 1:500, Abcam, Cambridge, UK, Cat#ab16667). Finally, the antigen–antibody reaction products can be observed under a microscope using the fluorescence reaction to determine the content and distribution of S100B and Ki67.

### Statistical Analysis

Each experiment was carried out with a minimum of three replicates. Statistical analysis of all data was conducted using GraphPad Prism 8 software. ImageJ was employed to process and analyze 2D images as well as fluorescence intensities. Unless otherwise specified, all data are presented as the mean ± standard deviation (SD). Multiple group comparisons were performed using one-way analysis of variance (ANOVA), followed by Tukey’s post hoc test. When comparing different groups, untreated samples served as controls to calculate significant differences, with * *p* < 0.05; ** *p* < 0.01; *** *p* < 0.001; **** *p* < 0.0001; ns, not significance.

## Supplementary Material



## Notes

Additional experimental methods, characterization; SEM images of LIG (S1); SEM images of PDMS, LIG/PDMS, and LIG-Cu/PDMS (S2); photos of the stretched LIG-Cu/PDMS patch (S3); survival rate of B16–F10 cells evaluated by the Live/Dead staining assay (S4); cell viability of B16–F10 cells against recycled LIG/PDMS and LIG-Cu/PDMS at different times under the stimulation of sunlight (S5); quantitative values for all phases (G0/G1, S, G2/M) of the cell cycle (S6); transwell assay results of B16–F10 cells subjected to sunlight stimulation at different time intervals using LIG-Cu/PDMS (S7); temperature changes of the skin beneath the patch in the first 5 min (S8); growth changes in tumor morphology in day 60 (S9); gene expression distribution map (S10); density plot of expression levels (S11); MA-plot distribution of DEGs (S12); quantitative analysis of samples (S13); gene expression levels under different experimental conditions (S14); clustering heatmap of DEGs in pathways related to melanoma initiation and progression (S15); pathway enrichment results of differentially expressed genes (S16); clustering heatmap of DEGs in pathways related to cuproptosis (S17); clustering heatmap of DEGs in pathways related to ferroptosis (S18); map of protein interactions of differentially expressed genes (S19); wb images and quantitative analysis of apoptosis-related proteins (pro-Caspase-3 and Bcl-2) (S20); images of reactive oxygen species (ROS) in cells using the DCFH-DA fluorescent probe (S21); 3D surface plot images of ROS in cells using the DCFH-DA fluorescent probe (S22); ratio of GSH/GSSG (S23); clustering heatmap of DEGs in pathways related to HIF-1α (S24); immunofluorescence images of proteins related to HIF-1α (S25); relative expression level of HIF-1α (S26); proportion of changes in mitochondrial membrane potential (S27); intracellular ATP concentration (S28); transmission electron microscopy images of B16–F10 cells (S29); relative expression of FDX1 in immunofluorescence (S30); relative expression of LIAS in immunofluorescence (S31); relative expression of HSP70 in immunofluorescence (S32); immunofluorescence images and quantitative analysis of proteins related to cuproptosis in different treatments (S33); fluorescence images of B16–F10 cells with various treatments of the BODIPY lipid ROS assay (S34); fluorescence images of B16–F10 cells treated with LIG-Cu/PDMS and Fer-1 of the BODIPY lipid ROS assay (S35); wb images and quantitative analysis of proteins treated related to ferroptosis with LIG-Cu/PDMS + sunlight and Fer-1 (S36); immunofluorescence images and quantitative analysis of CRT in tumor tissues across different treatment groups (S37); immunofluorescence images of HMGB1 in tumor tissues across different treatment groups (S38); ATP concentration in the cell culture supernatant (S39); the percentage distribution of macrophage subtypes (S40); IL-1β secretion by THP-1 treated with different treatments, as measured by ELISA (S41); representative flow cytometry images and quantification of IFN-γ^+^ CD8^+^ CD3^+^ cells (a) PD-1^+^ CD8^+^ CD3^+^ cells (b) under different treatments in the spleen region (S42); cell viability of MRC-5 cells against LIG/PDMS and LIG-Cu/PDMS after 60 min stimulation with or without sunlight (S43); comparison of antitumor ability and selectivity between LIG/PDMS and LIG-Cu/PDMS *in vitro* (S44); Cu^2+^ accumulation in the liver, kidney, and spleen (S45); hematoxylin-eosin staining (H&E staining) analysis of main organs from tumor-bearing mice after different treatments (S46); weight variations in healthy C57BL/6J mice under different treatments (S47); hematoxylin-eosin staining (H&E staining) analysis of main organs from healthy mice after different treatments (S48); hematology of healthy mice in the control and the LIG-Cu/PDMS phototherapy group in Day 45 (S49); serum biochemistry of healthy mice in the control and the LIG-Cu/PDMS phototherapy group in Day 45 (S50) (PDF)
