# Supplementary material for: A Stretchable, Transparent, Photothermally Stimulated Laser-Induced Graphene Patch for Noninvasive Skin Tumor Treatment
Source: ACS Nano. 2026 Mar 5;20(10):8671–90. doi: 10.1021/acsnano.5c21102 (PMC13001105; doi:10.1021/acsnano.5c21102)
Supplement: Supplementary file 1 [file nn5c21102_si_001.pdf]

Supporting Information

A stretchable, transparent, photothermally stimulated laser-induced graphene patch for non-invasive skin tumor treatment

Xiaoyu Xu[1]†, Le Cheng[2]†, Baoping Li[1]†, Xinyu Wang[3]†, Siyu Chen[1], Zihao Li[2], Li Zhou[4], Tengyue Liu[1], Yidan Zhou[5], Zhiqiang Li[1], Xin Li[3,*], Shi Chen[1,6,*], Meijia Gu[1,*], Ruquan Ye[2,*]

[1]Department of Neurosurgery, Zhongnan Hospital of Wuhan University, Ministry of Education Key Laboratory of Combinatorial Biosynthesis and Drug Discovery, School of Pharmaceutical Sciences, Wuhan University, Wuhan, Hubei, 430071, China.

[2]Department of Chemistry, State Key Laboratory of Marine Pollution, City University of Hong Kong, Hong Kong, 999077, China.

[3]Department of Gynecology, Renmin Hospital of Wuhan University& Ministry of Education Key Laboratory of Combinatorial Biosynthesis and Drug Discovery, School of Pharmaceutical Sciences, Wuhan University, Wuhan 430071, China.

[4]Department of Chemistry, State Key Laboratory of Marine Pollution, City University of Hong Kong, Hong Kong, 999077, China.

[5]Department of Epidemiology and Biostatistics, School of Public Health, Wuhan University, Wuhan 430071, China.

[6]Department of Critical Care Medicine, Intensive Care Unit, Shenzhen Key Laboratory of Microbiology in Genomic Modification & Editing and Application, Shenzhen Institute of Translational Medicine, Shenzhen University Medical School, Shenzhen Second People's Hospital, The First Affiliated Hospital of Shenzhen University, Shenzhen 518035, China

†These authors contributed equally to this work.

Materials and Methods

Preparation of LIG and LIG-Cu

PI film with a thickness of 120 μm (Zeman Tape Material Technology, China) was directly exposed to a 10.6 μm CO_2 laser (Minsheng Laser #MSDB-FM60 CO_2 Laser Marker, 60 W) to produce LIG. The laser was operated in vector mode with settings of 3 W power,1000 mm/s speed, 10 kHz frequency, 0.03 mm line spacing, and 5 pulses per dot. Subsequently, $CuCl_2$ aqueous solution with a concentration of 50 mg/mL was applied onto the fabricated LIG area and allowed to air dry. The loading content of $CuCl_2$ was maintained at 1 mg/cm^2. The copper salt-laden LIG was then subjected to a second lasing process to yield LIG-Cu sample.

Preparation of LIG/PDMS and LIG-Cu/PDMS

The PDMS prepolymer was obtained by mixing the PDMS oligomer and curing agent (Sylgard 184, Sigma-Aldrich) at a weight ratio of 10:1, followed by degassing. This transparent mixture was then spin-coated onto the LIG or LIG-Cu patterns with a spin speed of 1000 rpm for 15 s. After curing at 80°C for 2 h, the composites were submerged in liquid nitrogen to facilitate the detachment of the PDMS layer from the PI substrate, resulting in the production of LIG/PDMS or LIG-Cu/PDMS structures.

Characterization and test

The morphologies of the samples were analyzed using a QUATTRO S SEM, operating at 15 kV. Raman spectroscopy was conducted with a WITec RAMAN alpha 300R system, utilizing an excitation wavelength of 532 nm. XRD patterns were collected by a powder Rigakur X-ray diffractometer with Cu Kα radiation (λ = 1.54 Å). XPS spectra were acquired using a Thermo ESCALAB 250Xi spectrometer. Tensility tests were conducted using an Instron 5982 universal testing machine. Thin films of PDMS, LIG/PDMS, and LIG-Cu/PDMS were cut into dumbbell-shaped samples, with the

narrow region measuring 25 mm in length and 4 mm in width. The crosshead speed was set at 20 mm/min during the test.

Photothermal Stimulation Conditions

The infrared thermal images were taken with a thermal camera (CB360, GUIDE INFRARED, China). A 500 W Xenon lamp (GLORIA-X500A, Zolix, China) with an AM 1.5G filter and a parallel light spot diameter of 4.6 cm were used to simulate the standard solar spectrum closely. The emission spectrum covers 250–2500 nm, matching the ASTM G173-23 standard for terrestrial solar spectral irradiance. This power is calibrated with the Pofilai PL-MW 2000 high-intensity optical power meter.

Post-use Recovery, Disinfection, and Reuse Protocol of LIG-Cu/PDMS Patches

After each round of in *vitro* or *in vivo* experiments, the patch was first gently rinsed three times with sterile phosphate-buffered saline (PBS, pH 7.4) at room temperature, followed by shaking incubation in fresh sterile PBS for 15 min on a constant-temperature shaker (120 rpm, room temperature) to thoroughly remove residual biological debris, tumor tissue fragments, or culture medium components. Subsequently, the rinsed patch was immersed in 75% (v/v) ethanol solution for 30 minutes at room temperature to achieve effective disinfection. Following ethanol treatment, the patch was rinsed another three times with sterile PBS to eliminate any ethanol residue that might compromise biocompatibility in subsequent uses. Finally, the treated patch was allowed to air-dry completely under sterile conditions (in a laminar flow biosafety cabinet) and then stored under the same sterile conditions for at least one week before being applied to the next round of experiments.

Cell culture

B16-F10, MRC-5, and THP-1 cell lines were sourced from the China Center for Type Culture Collection (CCTCC). Cells were cultured in RPMI-1640 medium (Gibco,

Suzhou, China) containing 10% fetal bovine serum (FBS, 10099-141C, Gibco, Australia) supplemented with 100 U/mL penicillin and 100 μg/mL streptomycin (Biosharp, Hefei, China) in a humidified atmosphere (BB15 incubator, Thermo Dreieich, Germany) with 5% CO_2 at 37°C.

In Vitro cell Live/Dead staining

The cells were subjected to the same treatment protocol as in the CCK-8 assay and subsequently stained by using the Live & Dead Animal Cell Viability/Cytotoxicity Assay Kit (Calcein AM, propidium iodide). The stained cells were imaged with CLSM using a 488 nm laser and a 500–550 nm emission filter for the green fluorescence and a 561 nm laser with a 570–620 nm emission filter for the red fluorescence.

Cell apoptosis detection

Precooled PBS was used for centrifugal washing. 1–10×10^5 cells, including those from the culture supernatant, were collected. The cells were resuspended in 500 μL of 1× Binding Buffer. To each tube, 5 μL of Annexin V-FITC and 10 μL of propidium iodide (Liankebio, Hangzhou, China) were added. The mixture was gently vortexed and incubated at room temperature for 5 minutes, protected from light. Annexin V was detected through the FITC detection channel (excitation wavelength = 488 nm; emission wavelength = 530 nm), and propidium iodide was detected through the PE detection channel (excitation wavelength = 535 nm; emission wavelength = 615 nm).

Cell cycle analysis

Each group of cells was washed with PBS and then centrifuged at 1500 rpm for 5 minutes. The cell concentration was adjusted to 1×10^6/mL. 1 mL single-cell suspension was taken, centrifuged, and the supernatant was removed. Then 500 μL of 70% pre-cooled was added to the cell pellet, and the cells were fixed at 4°C for 2 hours to overnight. Before staining, the fixative was washed off with PBS. 100 μL of RNase A

solution was added to the cell pellet, the cells were resuspended, and incubation was carried out in a 37°C water bath for 30 minutes. Subsequently, 400 μL of PI staining solution (Solarbio, Beijing, China, Cat#CA1510) was added, mixed thoroughly, and incubated at 4°C for 30 minutes. The cell cycle was detected using flow cytometry, with red fluorescence recorded at an excitation wavelength of 488 nm. Analysis was performed using software FlowJo (10.8.1).

Transwell assay

The treated cells were digested with trypsin, resuspended in the medium containing 1% FBS, and counted. The cells were seeded into the upper chamber of a transwell insert according to the experimental groups, while complete medium with 10% FBS was added to the lower chamber. Incubation was carried out for 24 hours. After incubation, the medium was discarded, and the cells were washed once with PBS. The cells were fixed at room temperature with 4% paraformaldehyde for 20 minutes. The fixative was discarded. The cells were washed once with distilled water and stained with 0.1% (1 mg/mL) crystal violet solution for 2 minutes, followed by a final wash with distilled water. A cotton swab was used to remove non-migrated cells from the upper chamber, and the membrane was allowed to air dry. The membrane was carefully removed from the bottom of the insert using forceps, placed cell-side down on a glass slide, and mounted with neutral resin. The cells were observed and counted under a microscope.

GSH/GSSG detection assay

To determine the levels of reduced glutathione (GSH) and oxidized glutathione (GSSG), we employed the T-GSH/GSSG Assay Kit (Nanjing Jiancheng Bioengineering Institute, Nanjing, China, A061-1) following the manufacturer's instructions. B16-F10 cells were seeded into 6-well plates at a density of 1×10^5 cells per well and incubated for 24 hours. Subsequently, the cells underwent the same treatment procedure as that used for the

H_2O_2 assay. After the treatment, we measured the intracellular GSH levels using the GSH Assay Kit, strictly following the manufacturer's guidelines.

ATP concentration measurement

To detect the ATP concentration in cell, cell culture medium was removed from each group, and cells were lysed using the lysis buffer provided in the kit (Beyotime Biotechnology, Shanghai, China, Cat#S0026). To eliminate errors due to differences in protein content during sample preparation, protein concentration in the samples was determined using the BCA assay (Beyotime Biotechnology, Shanghai, China, Cat#P0012). The ATP concentration was then normalized to nmol/mg protein.

To detect the ATP released by the cells, the cell supernatant was collected. The other testing steps are the same as those mentioned above.

JC-1 mitochondrial staining

B16-F10 cells were seeded on confocal dishes and treated as specified, followed by the addition of 1 ml JC-1 staining solution (Beyotime Biotechnology, Shanghai, China, Cat#C2006) with thorough mixing. Cells were incubated at 37°C for 20 minutes in a cell incubator. After incubation, the supernatant was removed, and cells were washed twice with JC-1 staining buffer. Next, 2 mL of serum-containing cell culture medium was added, and the cells were observed under a laser confocal microscope at 490 nm and 530 nm excitation wavelengths and 525 nm excitation with 590 nm emission in two channels.

TEM imaging of B16-F10 cells

B16-F10 cells were treated the same protocol above with PBS (with or without Sunlight), LIG/PDMS (with or without Sunlight), LIG-Cu/PDMS (with or without Sunlight), and LIG-Cu/PDMS with Fer-1 or TTM (with Sunlight). The cells were fixed with 4% PFA and 2% glutaraldehyde in 0.1 M sodium cacodylate at 4 °C overnight.

The cells were subsequently post-fixed with 1% osmium tetroxide for 1 h and 1% uranyl acetate buffer for 1 h. Dehydration was performed using 25, 50, 70, 95, and 100 ethanol, and 100% propylene oxide sequentially and embedded in a gradually increasing Spurr resin: propylene oxide mixed solution (1:2 to 1:1) and finally 100% Spurr resin. Following this, the cells were cut into 90 nm ultrathin sections with Leica EM UC60, placed on a copper grid, and stained with uranyl acetate and led citrate. Observations and photographs were obtained using FEI Tecnai F30 and Gatan CCD digital micrograph.

qRT-PCR assay

All pipette tips and microcentrifuge tubes utilized in the RNA experiments were ensured to be RNase-free. Total RNA was isolated from tumor tissues using the TRIzol reagent following the manufacturer's protocol. The RNA concentration in each sample was measured with an Ultramicro Spectrophotometer (KaiAo, China). For cDNA synthesis, the microRNA Reverse Transcription Kit was employed. PCR amplification was conducted using a MicroRNAs qPCR Kit (Sangon, China). Target gene expression was quantified on a qRT-PCR system (Bio-Rad Laboratories, Hercules, CA, USA) with a universal reverse primer provided by the MicroRNAs qPCR Kit. The mRNA expression levels of the target genes were normalized against *β-actin*, and the relative fold-change was determined using the ΔΔCt method.

Quantification of cytokines in serum

The concentrations of 8 cytokines (IL-4, IL-17A, IL-6, IL-2, IL-12P70, IFN-γ, IL-1β, TNF-α) in mouse peripheral blood plasma were quantitatively analyzed using a flow cytometry bead-based assay (ABplex Mouse Cytokine 8-Plex Assay Kit, ABclonal Technology, Wuhan, China; RKO4863).

Lipid peroxidation assay

B16-F10 cells were cultured in confocal dishes and treated following the same procedure as described for the JC-1 mitochondrial staining assay, with the addition of a group treated with the ferroptosis inhibitor Ferrostatin-1 (Fer-1) (Beyotime, Shanghai, China, Cat#Y240805). After treatment, lipid peroxidation levels were detected using Lipid Peroxidation Assay Kit with BODIPY 581/591 C11 (Beyotime, Shanghai, China, Cat# S0043S) according to the manufacturer's instructions. The cells were then observed under a laser confocal microscope with excitation wavelengths of 490 nm and 530 nm, and emissions were captured at 525 nm and 590 nm in two separate channels.

Western blotting with specific inhibitor validation

The ferroptosis inhibitor Ferrostatin-1 (Fer-1) (Beyotime, Shanghai, China, Cat#Y240805) was applied at a concentration of 10 μM. All other experimental procedures, including membrane incubation and detection, were consistent with the Western blotting methodology described previously. Specifically, the membranes containing the target protein were incubated with primary antibodies against GAPDH (mouse, 1:5000, HuaBio, Hangzhou, China, Cat#ET1601-4), KEAP1 (mouse, 1:1000, ABclonal, Wuhan, China, Cat#A17062), and GPX4 (mouse, 1:1000, ABclonal, Wuhan, China, Cat#A11243) at 4°C for 16 hours to ensure accurate detection. Subsequently, the membranes were treated with HRP-conjugated Anti-Rabbit IgG (mouse, 1:100000, HuaBio, Hangzhou, China, Cat#HA1001) at room temperature for 1 hour. Protein bands were visualized using enhanced chemiluminescence imaging (Bio-Rad, Hercules, CA, USA).

The Caspase-3 inhibitor Z-DEVD-FMK (Ambeed, America, A301584) was applied at a concentration of 100 μM. All other experimental procedures, including membrane incubation and detection, were consistent with the Western blotting methodology described previously. Specifically, the membranes containing the target protein were incubated with primary antibodies against GAPDH (mouse, 1:5000, HuaBio, Hangzhou, China, Cat#ET1601-4), Caspase-3 (mouse, 1:1000, CST, America, 9662S), and Bcl-2 (mouse, 1:1000, ABclonal, Wuhan, China, A19693) at 4°C for 16 hours to ensure

accurate detection. Subsequently, the membranes were treated with HRP-conjugated Anti-Rabbit IgG (mouse, 1:100000, HuaBio, Hangzhou, China, Cat#HA1001) at room temperature for 1 hour. Protein bands were visualized using enhanced chemiluminescence imaging (Bio-Rad, Hercules, CA, USA).

Assessment of Tumor Recurrence

The animal experiments were approved by the Institutional Animal Care and Use Committee (IACUC) of Wuhan University (Approval No. WP20220020). To evaluate tumor recurrence following treatment, 2×10^5 B16-F10 cells were subcutaneously injected into the right femoral region of C57BL/6 mice. One week later, C57BL/6 mice bearing tumors (60–80 mm^3) were randomized into five groups (n = 3): (a) untreated group; (b) LIG-Cu/PDMS group; (c) LIG/PDMS + Sunlight group; (d) LIG-Cu/PDMS + Sunlight group. In groups (b), (c) and (d), 1 cm $\times$ 1 cm patches were applied to the skin. Mice in groups (c) and (d) were then irradiated with simulated Xenon light at 1.5 kW/m^2 for 60 minutes, while group (b) mice were kept in the dark. Treatments were administered on day 1 and day 5. Body weight and tumor volume ($V = 0.5 \times \text{length} \times \text{width}^2$) were monitored and recorded throughout the 2-month experimental period, with photographs taken to document tumor changes. Animals were euthanized at the end of the experiment, and tumors, together with major organs, were collected for further analysis.

Immunofluorescence Staining

The infiltration of $CD3^+$, $CD4^+$, and $CD8^+$ cells in the tissue and the expression of HMGB1 and CRT were evaluated by immunofluorescence staining. In brief, tumor tissues from the animal models of each group were fixed in formalin and embedded in paraffin. Sections of 4 μm thickness were baked at 65°C for 2 hours, deparaffinized in xylene, and rehydrated through a graded ethanol series (100%, 95%, 85%, and 75%), followed by PBS washing. For antigen retrieval, the sections were microwaved in citrate buffer (pH=6.0) for 15 minutes. Subsequently, the sections were blocked with 3% BSA at 37°C for 30 minutes and then incubated with primary antibodies overnight

at 4°C. Antibodies targeting $CD3^+$(), $CD4^+$(Abcam, ab16669, 1:200, TYR-488), $CD8^+$(Abcam, ab217344, 1:200, TYR-488), HMGB1 (Servicebio, GB11103, 1:200, TYR-651), and CRT (Abways, CY5353, 1:200, TYR-488) were used in this study. After washing three times with PBS, the sections were incubated with secondary antibodies and stained with Hoechst for 15 minutes at room temperature in the dark. Finally, the stained cells were visualized using confocal laser scanning microscopy.

Quantification of copper ions in biological samples

Upon completion of the animal experiments, liver, kidney, spleen, and serum samples were collected from each group. Copper ion levels in these samples were determined following the instructions provided with the Copper Ion Detection Assay Kit (Beyotime, Shanghai, China, Cat# S1075S), using a microplate reader (PerkinElmer, MA, USA).

Immunofluorescence staining with specific inhibitor validation

To further validate the experimental results, the cuproptosis inhibitor ammonium tetrathiomolybdate (TTM) (AbMole BioScience, Houston, TX, USA Cat#15060-55-6) was applied at a concentration of 20 μM. All other procedures were consistent with the previously described immunofluorescence staining method. The experimental steps and principles of immunofluorescence are similar to those of immunohistochemistry, relying on the highly specific binding between primary antibodies, LIAS (rat, 1:500, Abcam, Cambridge, UK, Cat#ab16667), DLAT (rat, 1:100, Abways, Shanghai, China, Cat#CY8125), and FDX1 (rat, 1:500, Abcam, Cambridge, UK, Cat#ab16667), and the secondary antibody, Goat Anti-Rabbit IgG (Cy3) (rat, 1:500, Abcam, Cambridge, UK, Cat#ab16667). Different target antibodies were labeled with fluorescein, and the expression levels and distribution patterns of LIAS, DLAT, and FDX1 were observed under a fluorescence microscope.

Flow cytometry analysis for tissues

The spleen tissues were immediately removed after euthanasia and placed in pre-cooled PBS. The spleen tissue was crushed (1200 rpm, 2 minutes, 4°C) with a tissue disruptor.

The suspension was collected and centrifuged (400 × g, 5 minutes, 4°C). The precipitate was incubated on ice with red blood cell lysis buffer for 1–2 minutes, then immediately terminated with PBS (3–5 times the volume of red blood cell lysis buffer). It was centrifuged, and the supernatant was removed through a 40 μm cell sieve. After counting the cells, add the corresponding amount of antibody. The types of T cells were analyzed by flow cytometry using Elab Fluor® Violet 450 anti-Mouse CD3 antibodies, PE anti-Mouse CD4 antibody, APC anti-Mouse CD8a antibody, FITC anti-Human/Mouse CD44 antibody, and Elab Fluor®700 anti-Mouse CD62L antibody. Elab Fluor® 700 anti-Mouse PD-1 antibody, and PE anti-Mouse IFN-γ antibody. 200 μL of staining buffer was added to the blank tube, single-stain tube, and experimental tube to resuspend the cells. 2 μL of $CD3^+$, $CD4^+$, $CD8a^+$, $CD44^+$, $CD62L^+$, and $PD\text{-}1^+$ antibody was added to each tube, vortexed for 3 seconds, and incubated at room temperature in the dark for 15 minutes. Then, the cells that had undergone surface marker staining were fixed and permeabilized for subsequent intracellular staining with an IFN-γ antibody, following the manufacturer's protocol (E-CK-A109, Elabscience Biotechnology). 1 mL of staining buffer was added to each tube, centrifuge at 500 × g for 5 minutes at 4°C, and the supernatant was discarded. 200 μL of staining buffer was added to each tube, vortexed to resuspend the cells, and cell typing analysis was performed using flow cytometry.

Supplementary Figures

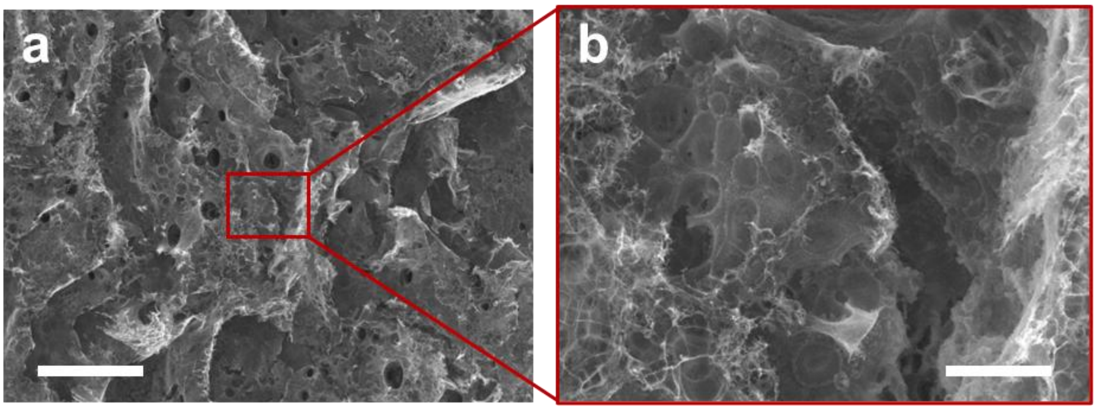

Figure S1. SEM images of (a, b) LIG. Scale bars: 30 μm for a and 5 μm for b.

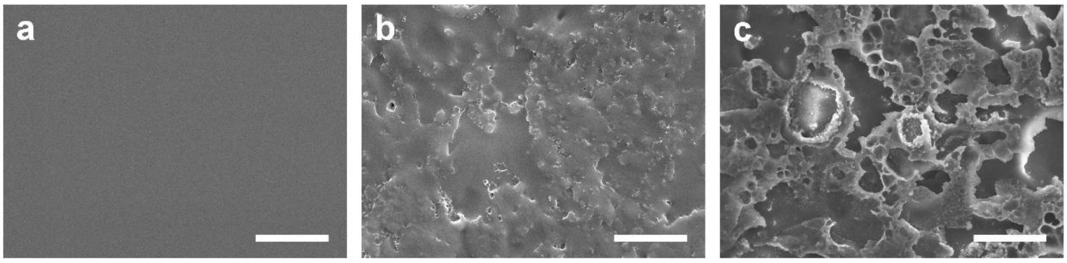

Figure S2. SEM images of (a) PDMS, (b) LIG/PDMS, and (c) LIG-Cu/PDMS. Scale bars: 10 μm.

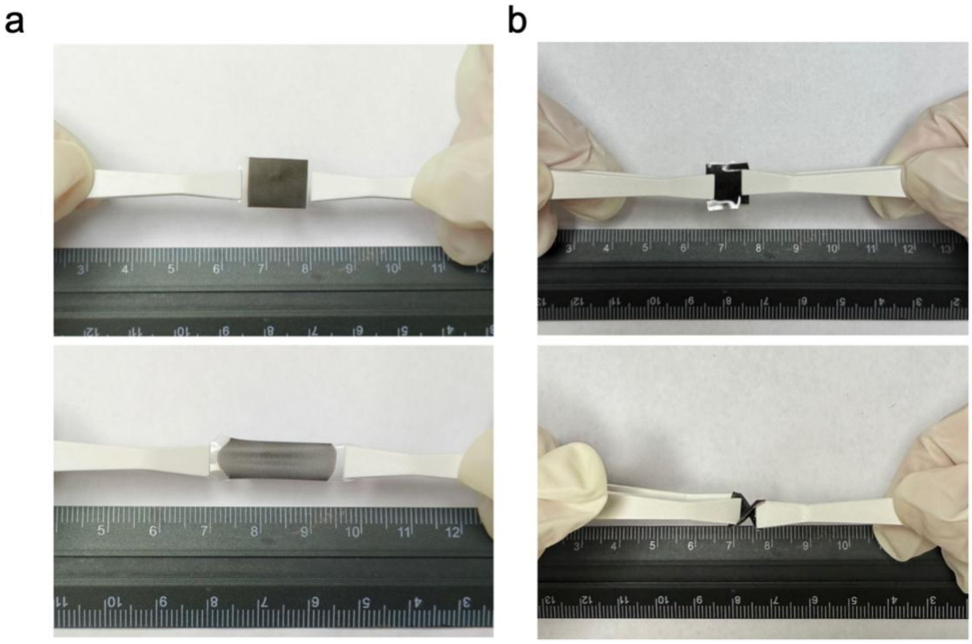

Figure S3. Photos of the stretched (a) and distorted (b) LIG-Cu/PDMS patch.

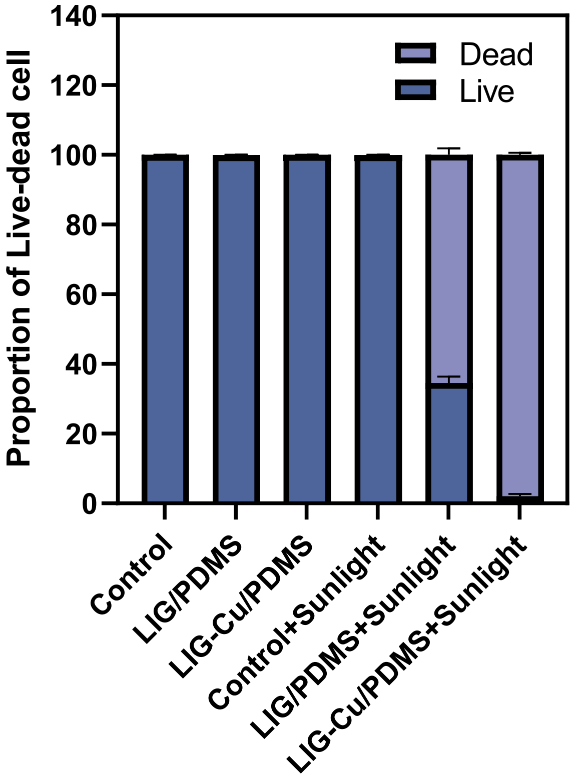

Figure S4. Survival rate of B16-F10 cells evaluated by the Live/Dead staining assay (*n* = 3). Data are presented as mean ± SD.

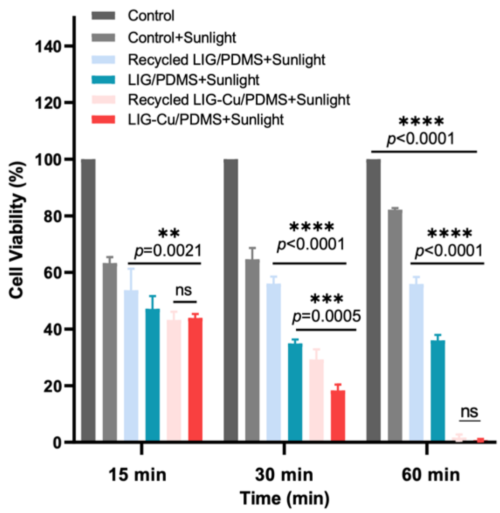

Figure S5. Cell viability of B16-F10 cells against recycled LIG/PDMS and LIG-Cu/PDMS at different times under the stimulation of Sunlight (*n* = 3). Data are presented as mean ± SD. ****p<0.0001; ***p<0.001; **p<0.01; *p<0.05; ns, no significance.

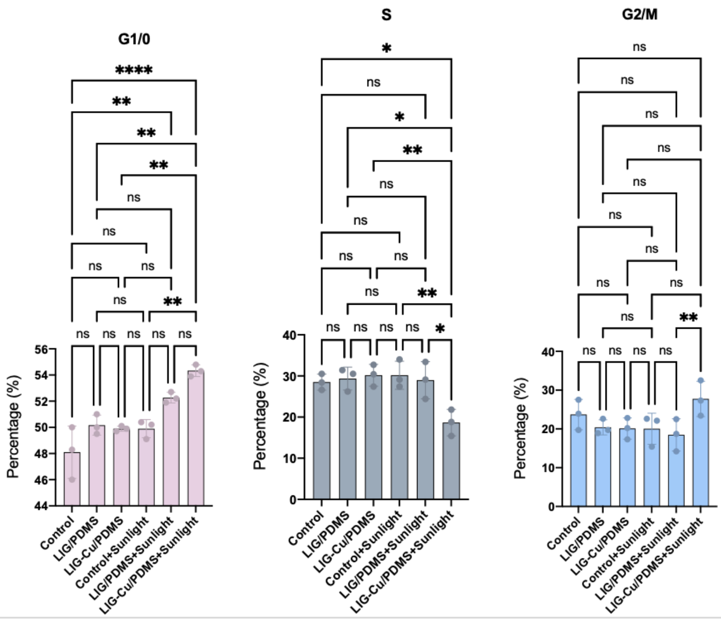

Figure S6. Quantitative values for all phases (G0/G1, S, G2/M) of the cell cycle (*n* = 3). Data are presented as mean ± SD**.** ****p<0.0001; ***p<0.001; **p<0.01; *p<0.05; ns, no significance.

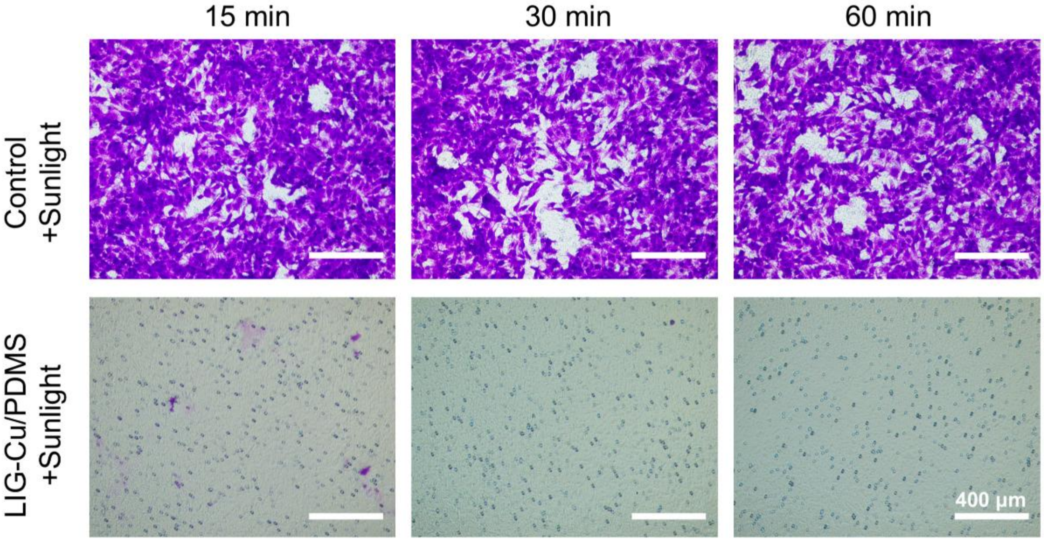

Figure S7. Transwell assay results of B16-F10 cells subjected to Sunlight stimulation at different time intervals using LIG-Cu/PDMS. Scale bars: 400 μm.

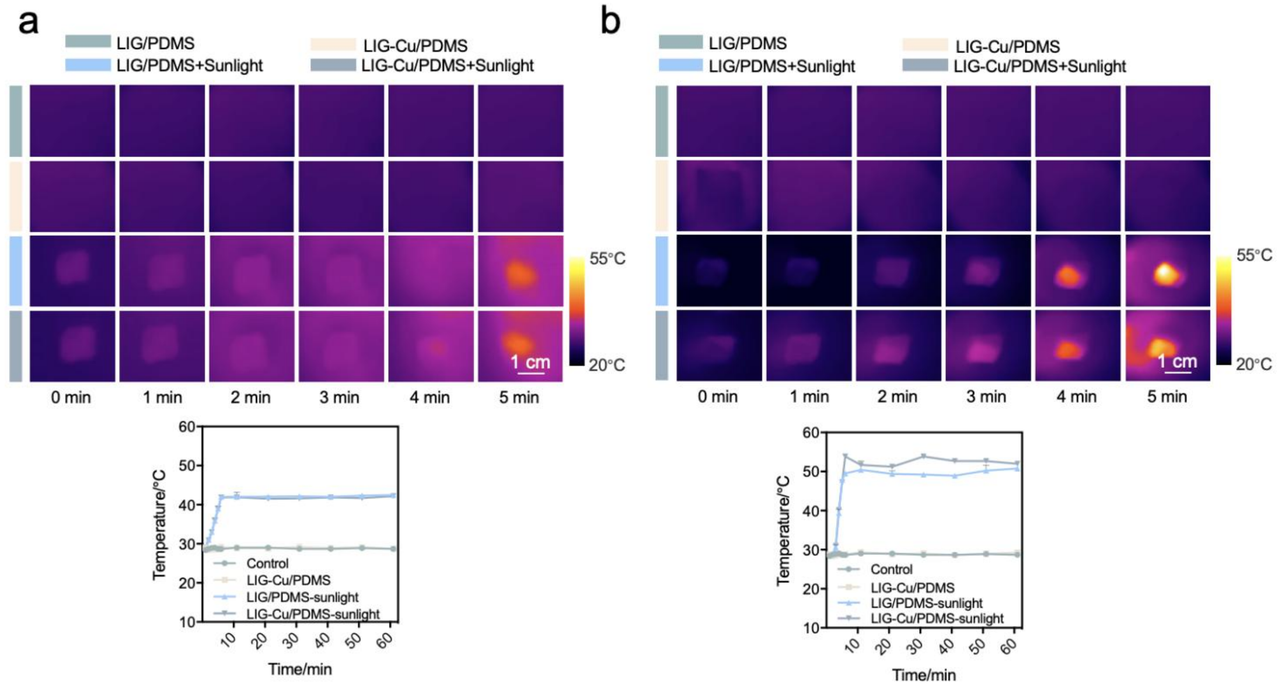

Figure S8. Temperature changes of the skin beneath the patch (a) and the patch (b) in 60 minutes. Scale bars: 1 cm.

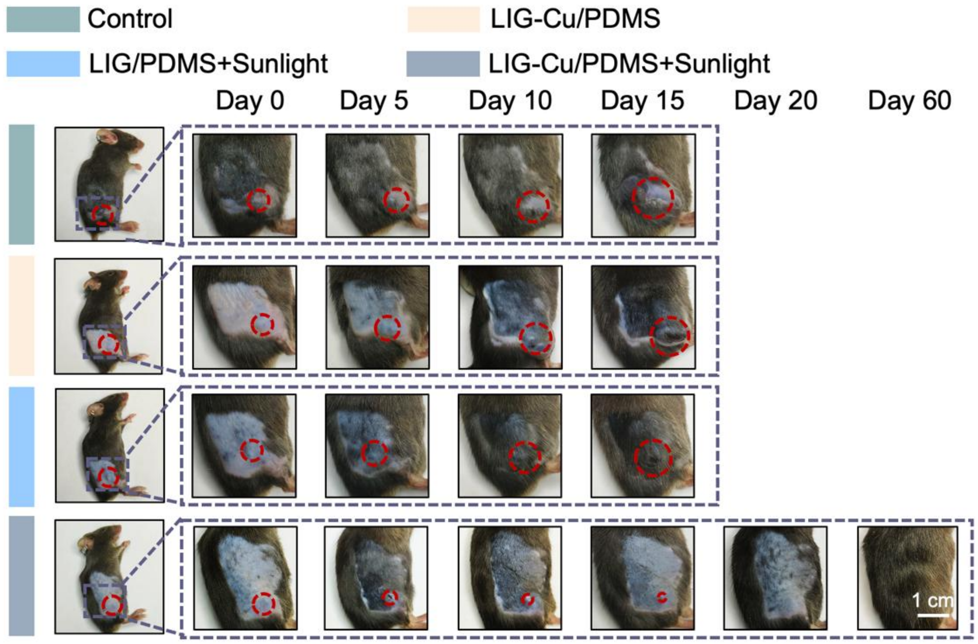

Figure S9. Growth changes in tumor morphology in Day 60. Scale bars: 1 cm.

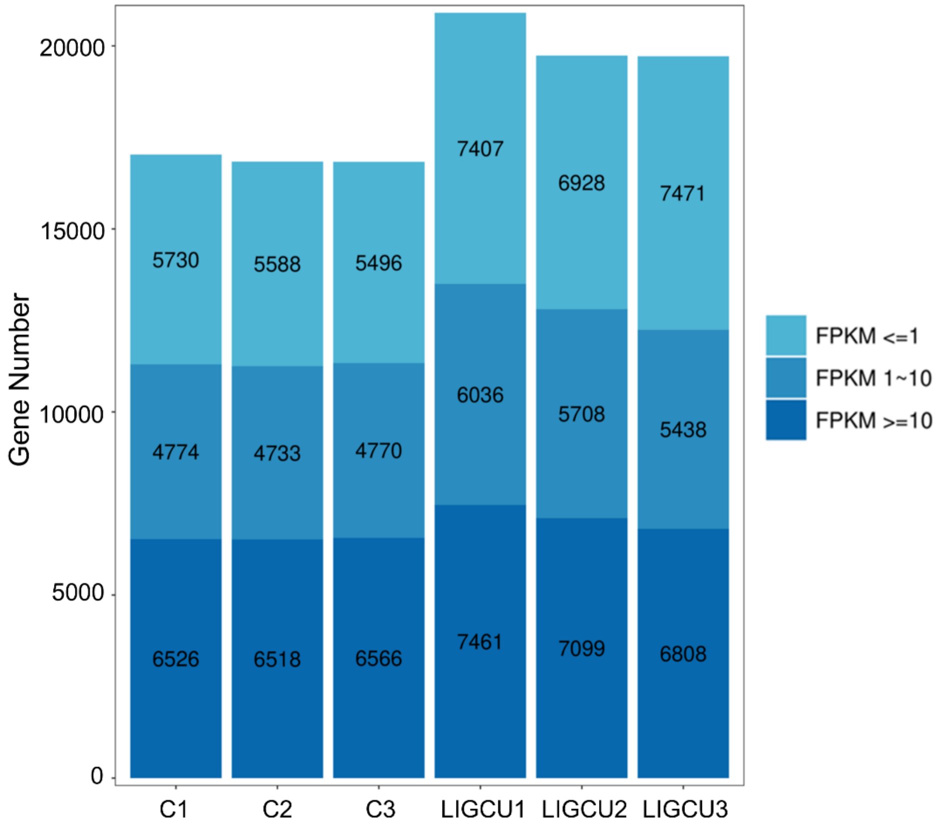

Figure S10. Gene expression distribution map. X axis represents the name of the sample (C: Control, LIGCU: LIG-Cu/PDMS + Sunlight), the Y axis represents the number of genes, and the color depth represents different expression levels: FPKM <= 1 is the gene with very low expression level, FPKM between 1-10 is the gene with low expression level, and FPKM >= 10 is the gene with medium and high expression level ($n = 3$).

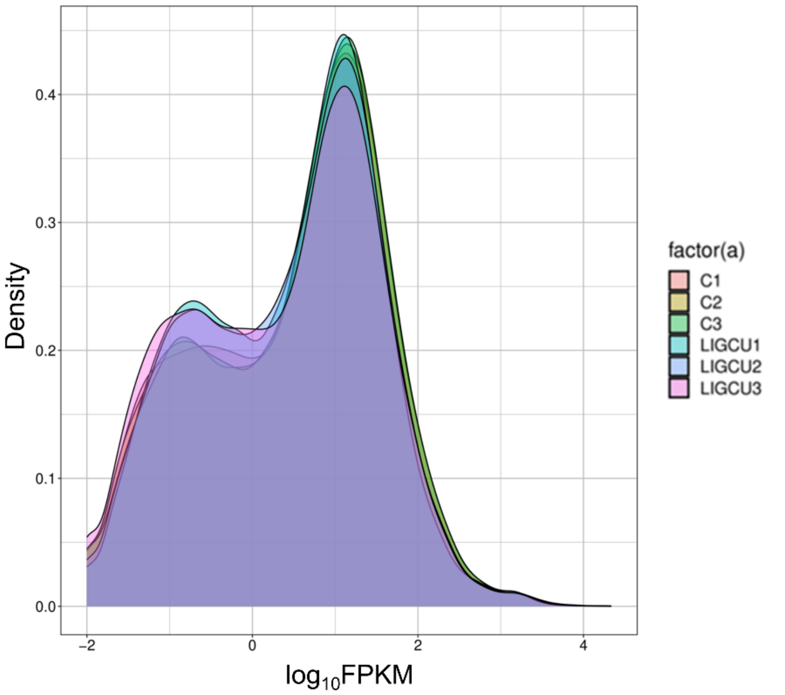

Figure S11. Density plot of expression levels (C: Control, LIGCU: LIG-Cu/PDMS + Sunlight) (*n* = 3). The X-axis represents log_{10}FPKM, while the Y-axis denotes gene density, which is the proportion of genes at a given expression level relative to the total number of expressed genes.

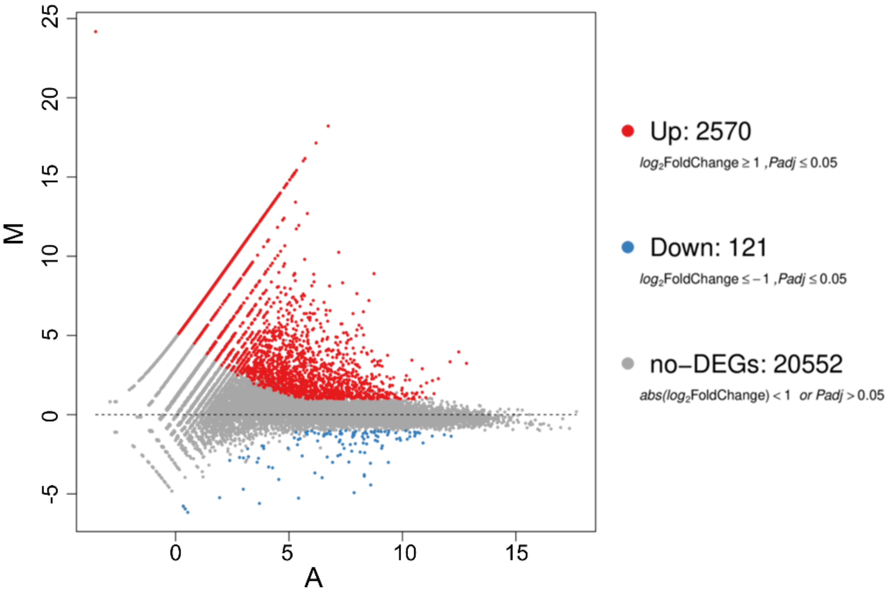

Figure S12. MA-plot distribution of DEGs (Differentially Expressed Genes). (C: Control, LIGCU: LIG-Cu/PDMS + Sunlight) (n = 3). A (Add) = [log2(C) + log2 (LIGCU)] / 2; M (Minus) = log2(C) - log2 (LIGCU) = log2 (C / LIGCU).

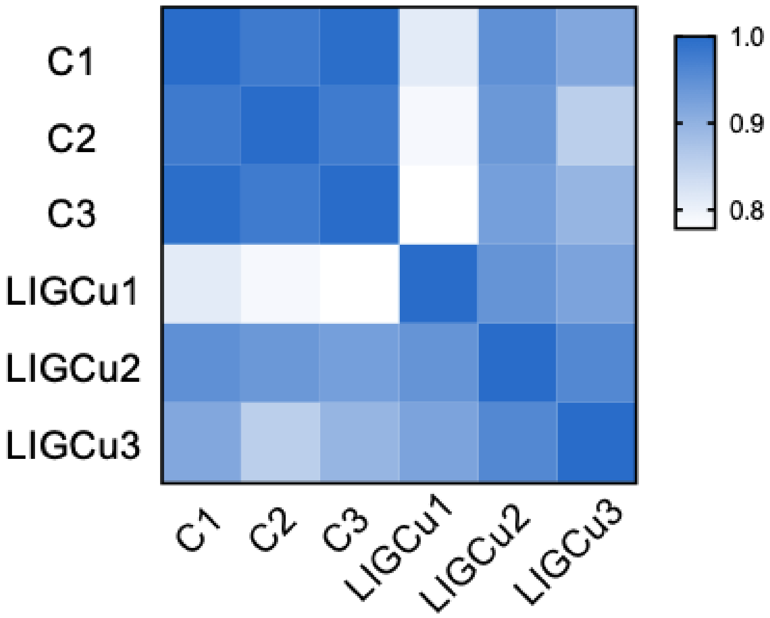

Figure S13. Quantitative analysis of samples. The heat map shows the correlation analysis between the different samples (C: Control, LIGCU: LIG-Cu/PDMS + Sunlight) (n = 3). Both horizontal and vertical axes represented each sample. Different colors represented different correlation coefficients.

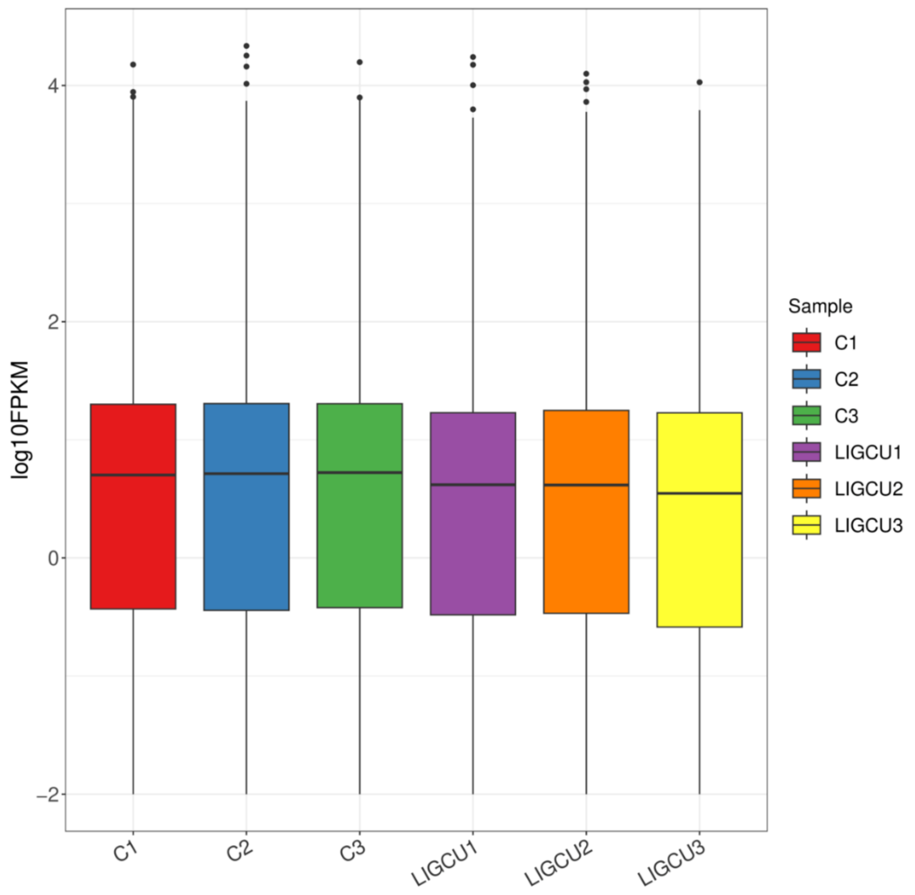

Figure S14. Gene expression levels under different experimental conditions (C: Control, LIGCU: LIG-Cu/PDMS + Sunlight) (*n* = 3).

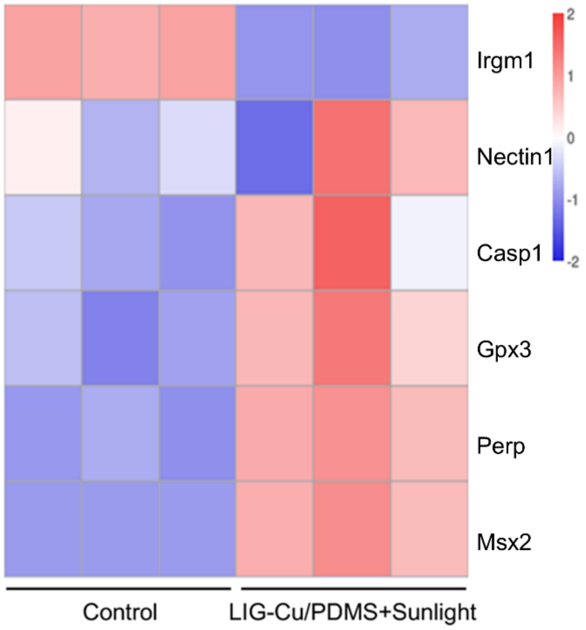

Figure S15. Clustering heatmap of DEGs in pathways related to melanoma initiation and progression (*n* = 3).

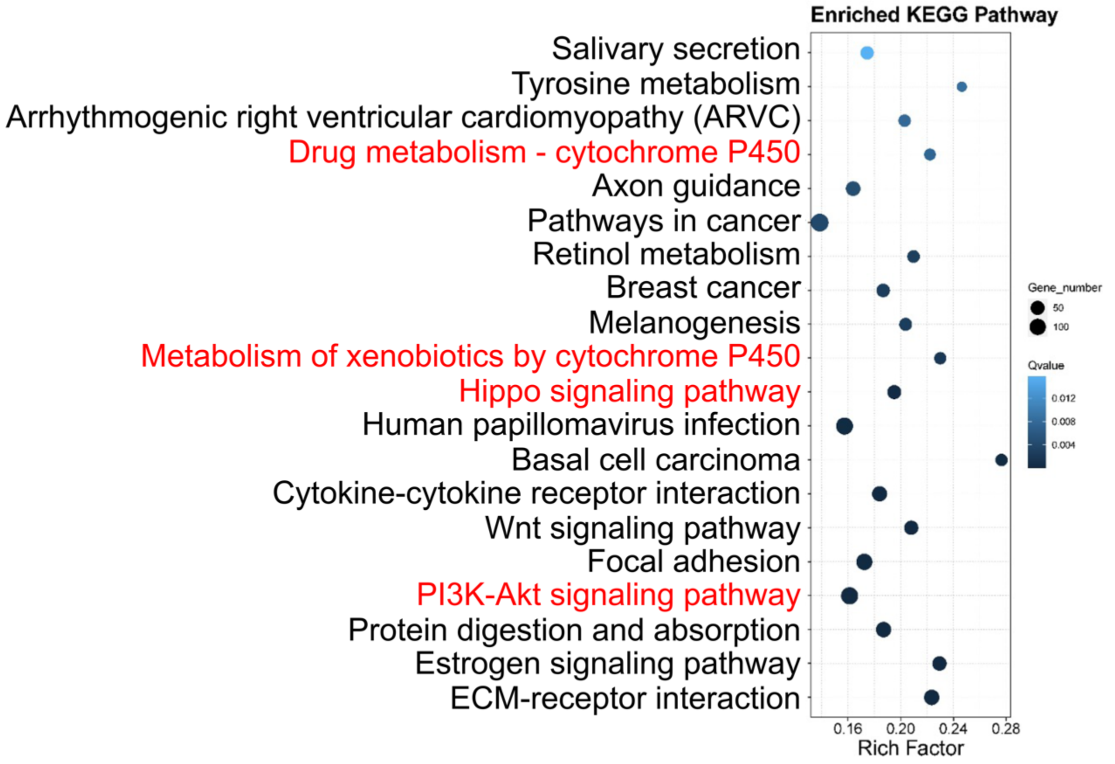

Figure S16. Pathway enrichment results of differentially expressed genes (*n* = 3).

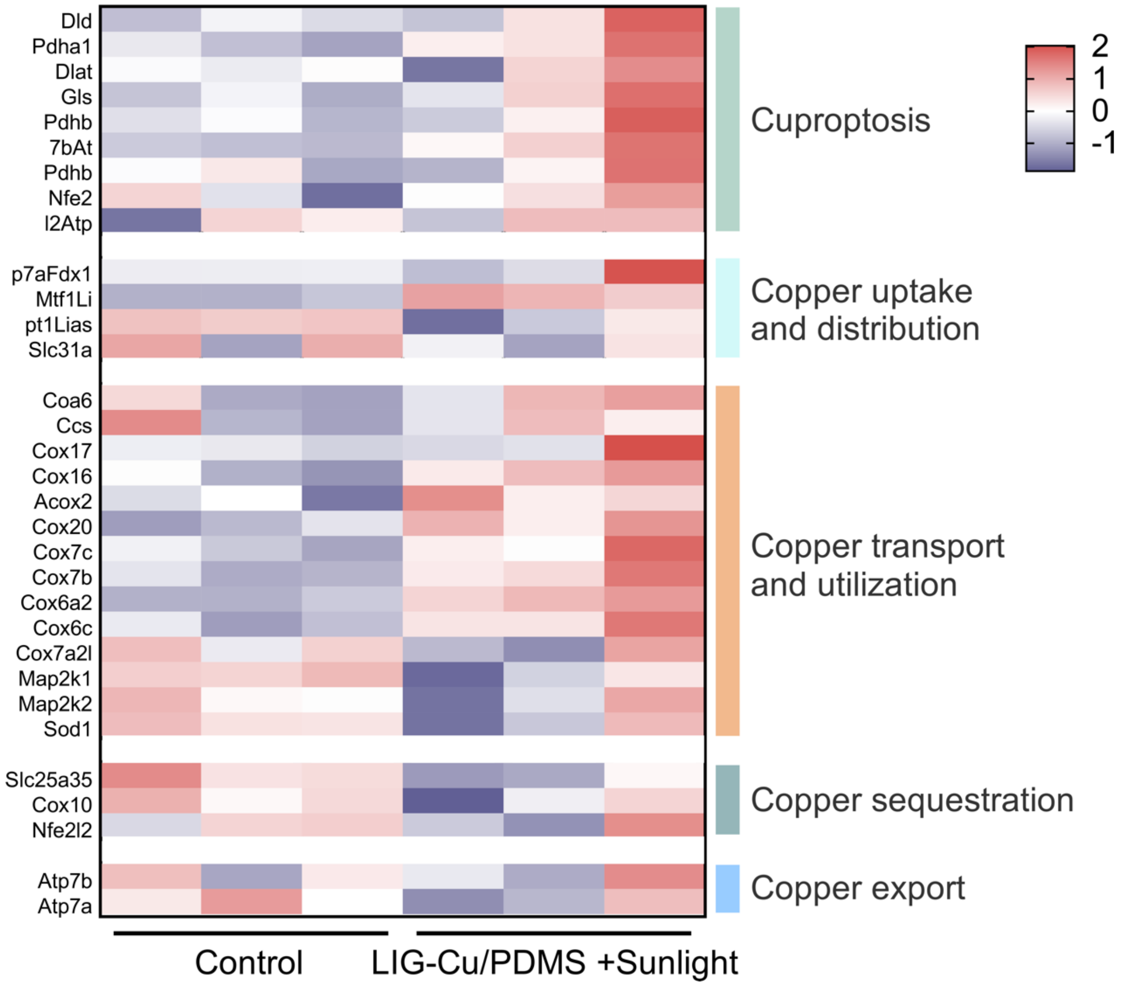

Figure S17. Clustering heatmap of DEGs in pathways related to cuproptosis (*n* = 3).

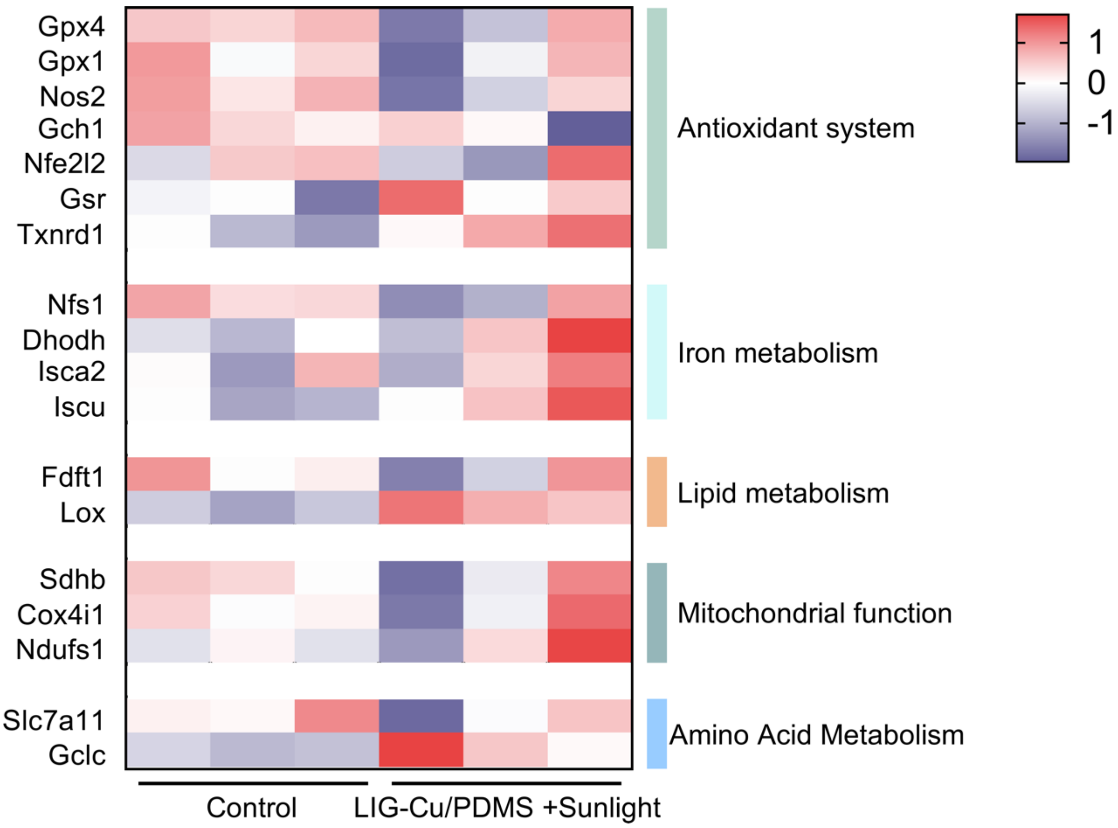

Figure S18. Clustering heatmap of DEGs in pathways related to ferroptosis (*n* = 3).

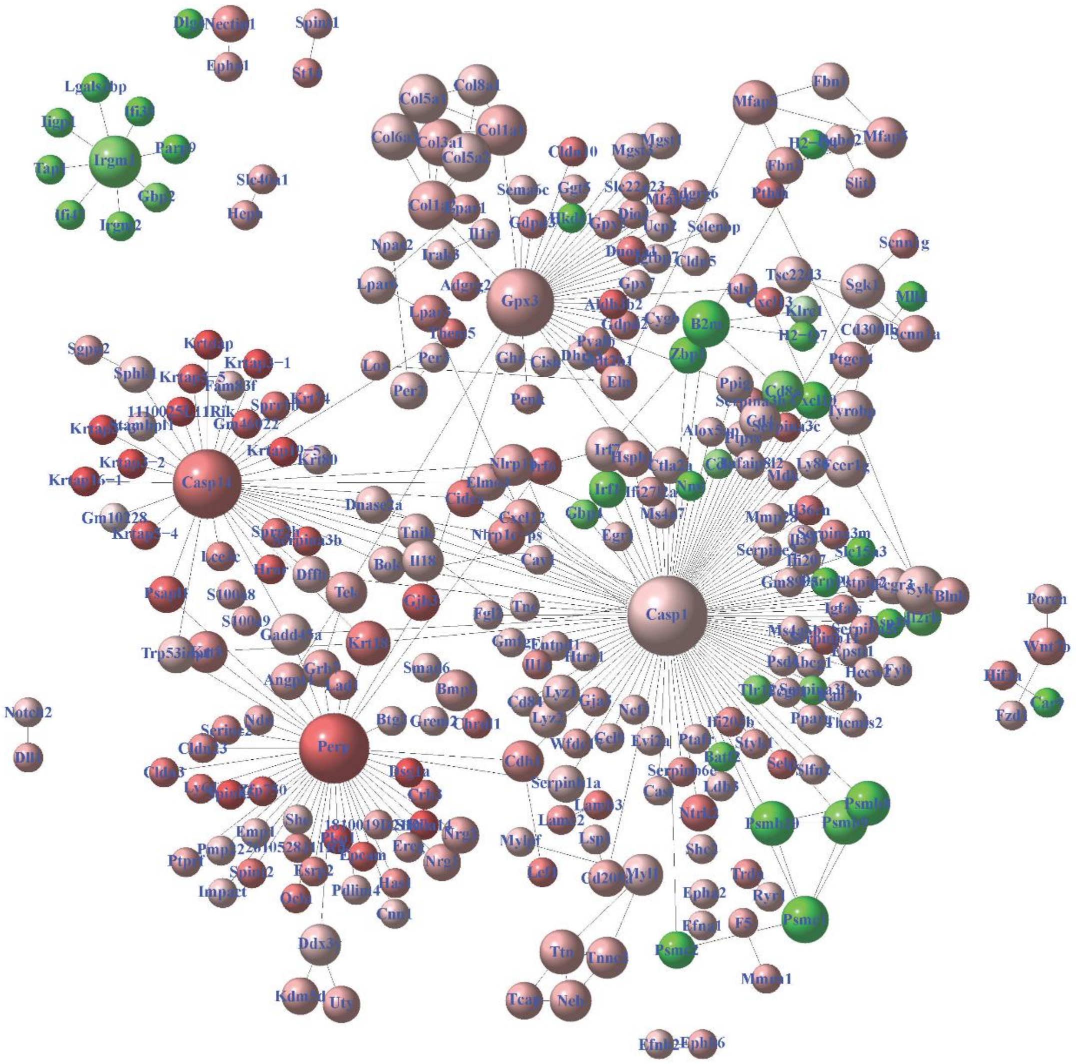

Figure S19. Map of protein interactions of differentially expressed genes.

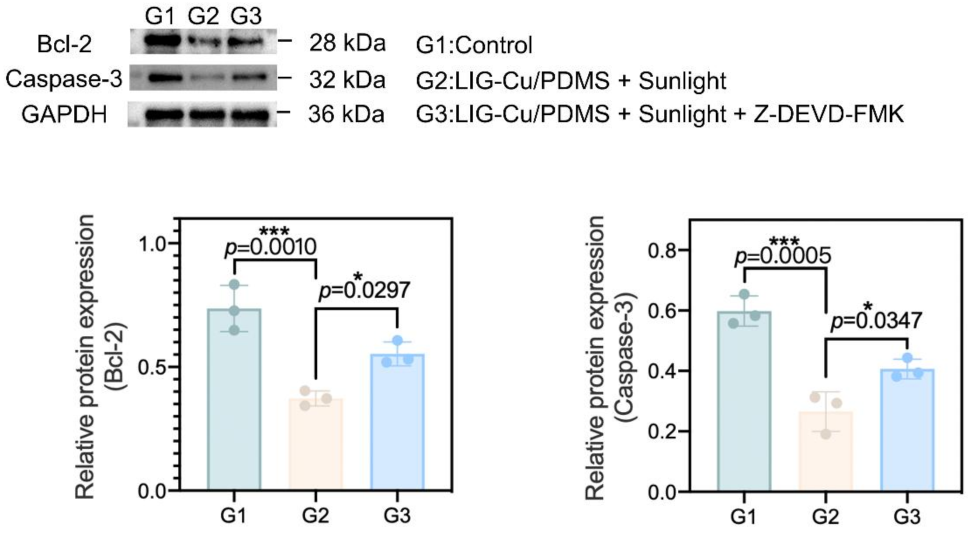

Figure S20. Western blot images and quantitative analysis of apoptosis-related proteins (pro-Caspase-3 and Bcl-2) in melanoma cells treated with LIG-Cu/PDMS + sunlight, with or without Z-DEVD-FMK pretreatment. Data are presented as mean ± SD. *****p*<0.0001; ****p*<0.001; ***p*<0.01; **p*<0.05; ns, no significance. *n* = 3 biologically independent cell samples per group.

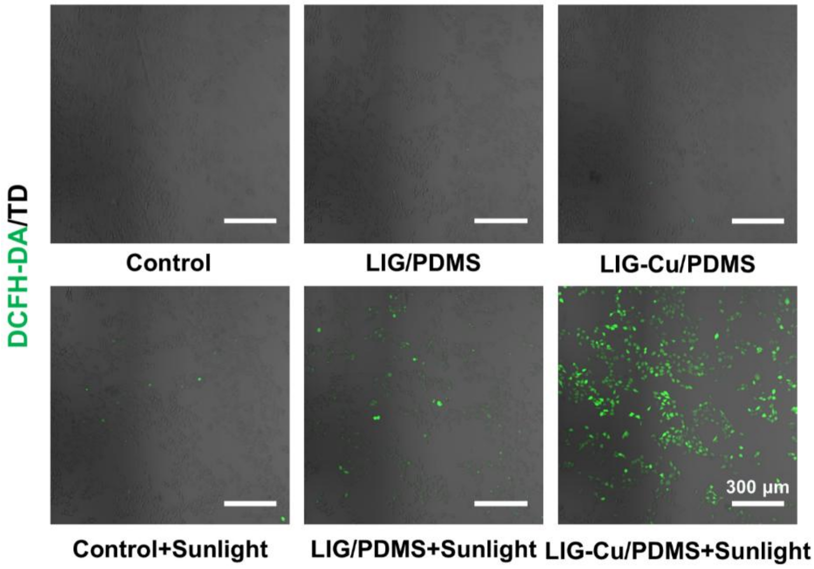

Figure S21. Images of reactive oxygen species in cells using the DCFH-DA fluorescent probe. Scale bars: 300 μm.

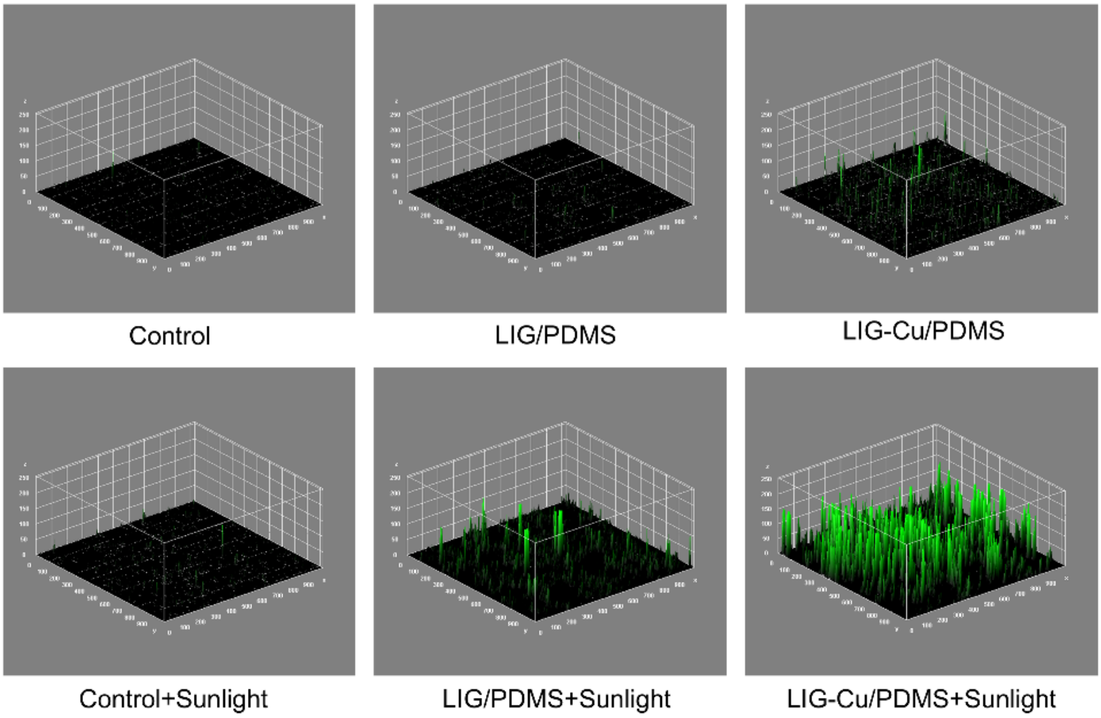

Figure S22. 3D surface plot images of ROS in cells using the DCFH-DA fluorescent probe.

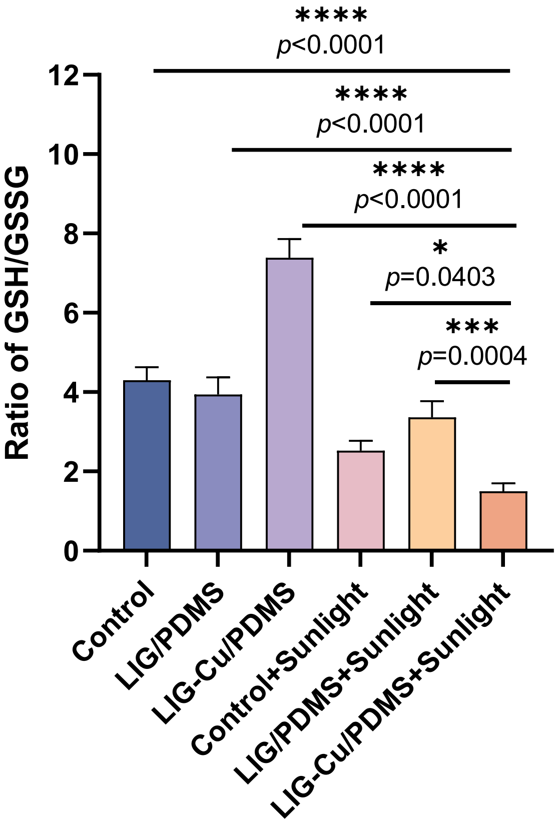

Figure S23. Ratio of GSH/GSSG (*n* = 3). Data are presented as mean ± SD. ****p<0.0001; ***p<0.001; **p<0.01; *p<0.05; ns, no significance.

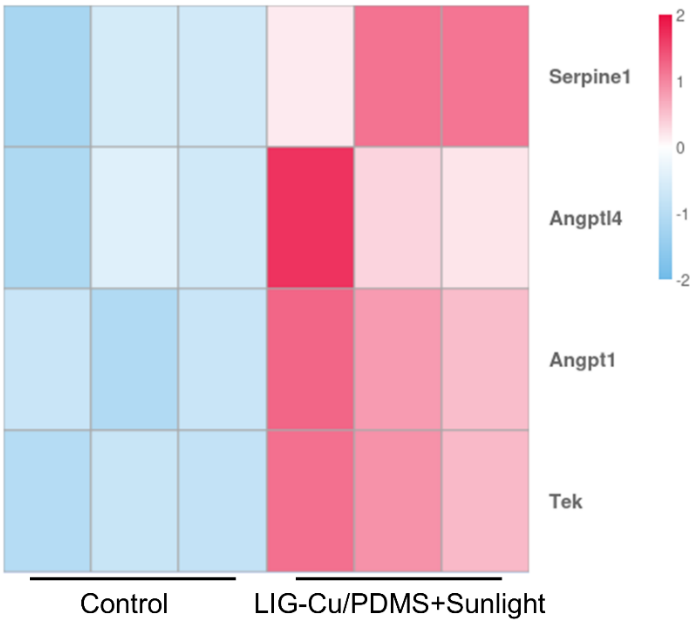

Figure S24. Clustering heatmap of DEGs in pathways related to HIF-1α (*n* = 3).

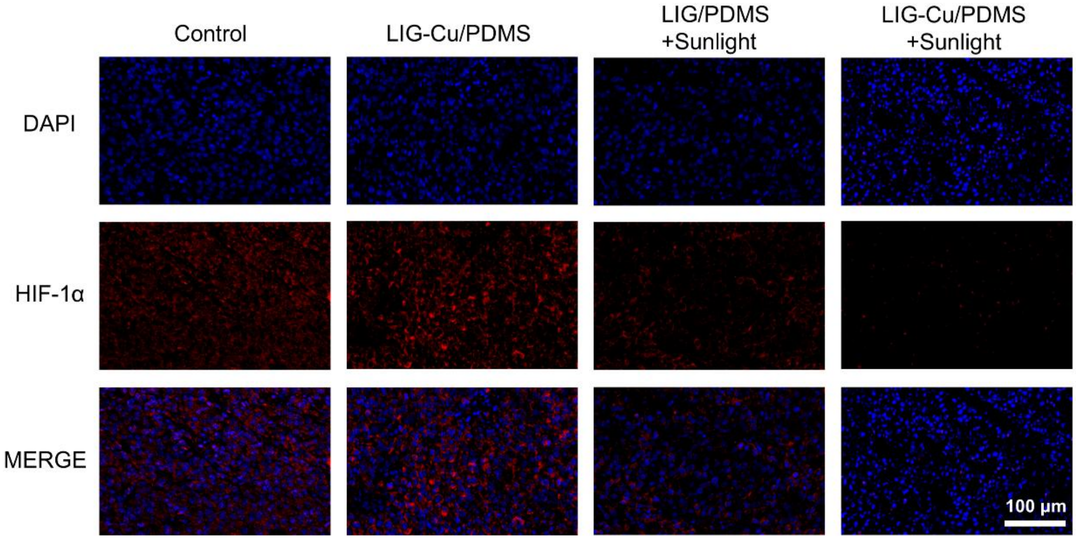

Figure S25. Immunofluorescence images of proteins related to HIF-1α. Scale bar: 100 μm.

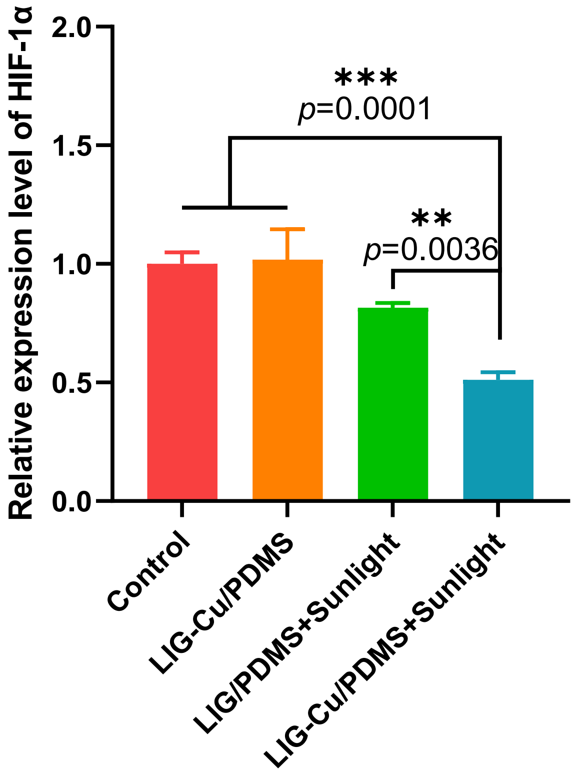

Figure S26. Relative expression level of HIF-1α (*n* = 3). Data are presented as mean ± SD. ****p<0.0001; ***p<0.001; **p<0.01; *p<0.05; ns, no significance.

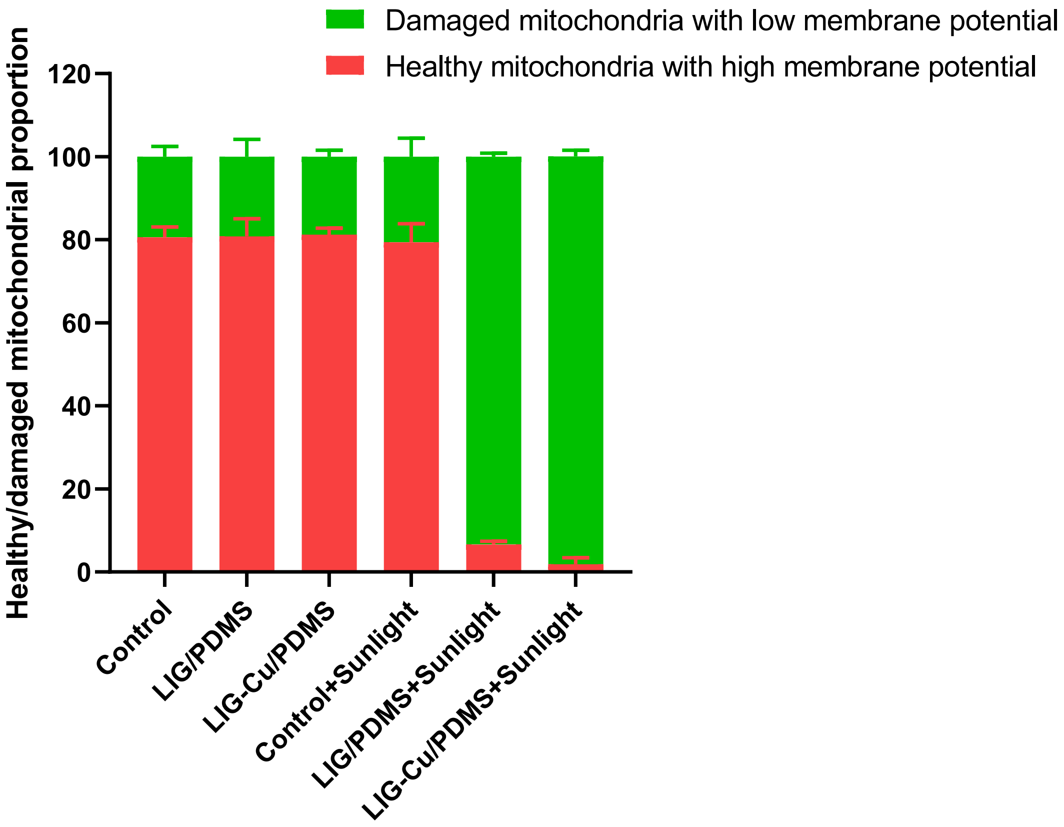

Figure S27. Proportion of changes in mitochondrial membrane potential (Δψm). Red fluorescence intensity indicates healthy mitochondria with high membrane potential, while green fluorescence intensity represents mitochondria that are damaged or depolarized, exhibiting low membrane potential (*n* = 3). Data are presented as mean ± SD.

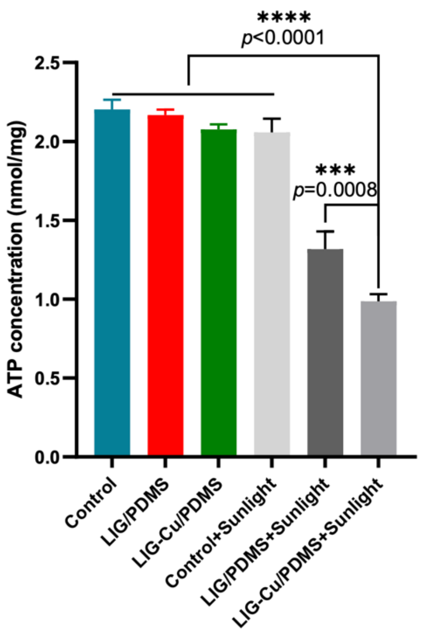

Figure S28. Intracellular ATP concentration (*n* = 3). Data are presented as mean ± SD. ****p<0.0001; ***p<0.001; **p<0.01; *p<0.05; ns, no significance.

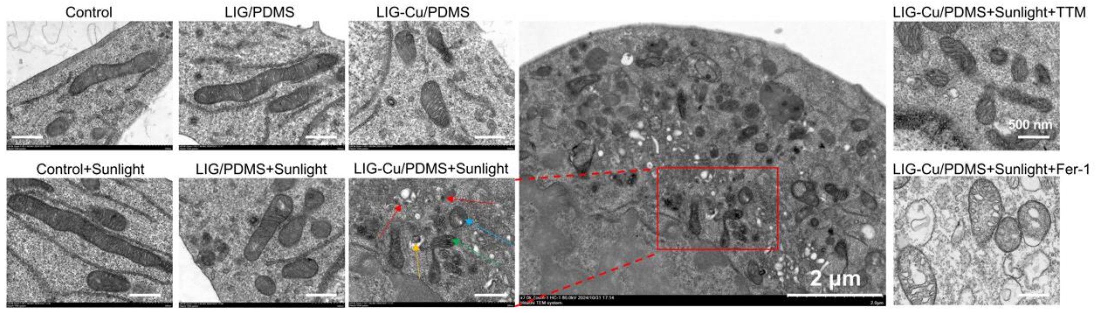

Figure S29. Transmission electron microscopy images of B16-F10 cells. Scale bar: 500 nm and 2 μm. Green arrow indicates the characteristic dense mitochondrial structure with remarkably reduced cristae and smaller volume; red arrows indicate autophagy of the endoplasmic reticulum, the yellow arrow shows autophagy of mitochondria, and the blue arrow indicates the pronounced swelling of the mitochondrial matrix.

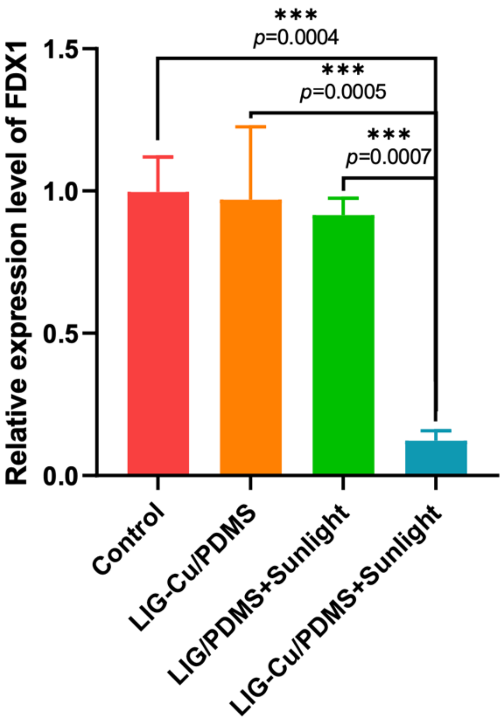

Figure S30. Relative expression of FDX1 in immunofluorescence (n = 3). Data are presented as mean ± SD. ****p<0.0001; ***p<0.001; **p<0.01; *p<0.05; ns, no significance. Parallel tests on the same sample under the same conditions.

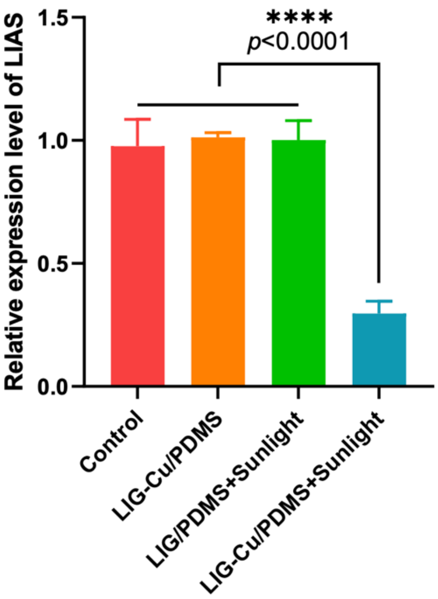

Figure S31. Relative expression of LIAS in immunofluorescence (*n* = 3). Data are presented as mean ± SD. ****p<0.0001; ***p<0.001; **p<0.01; *p<0.05; ns, no significance. Parallel tests on the same sample under the same conditions.

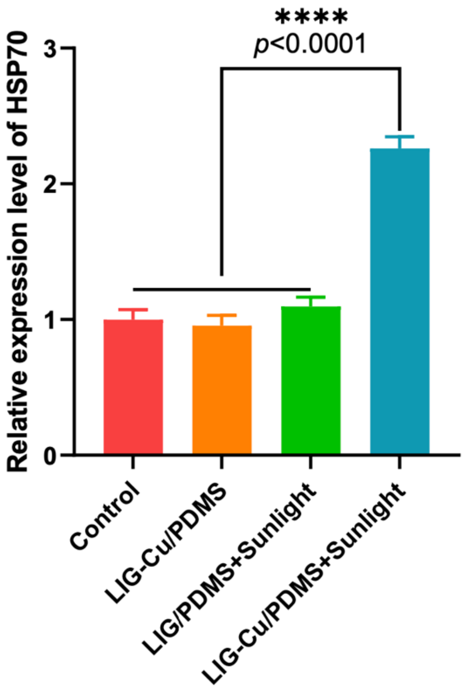

Figure S32. Relative expression of HSP70 in immunofluorescence (*n* = 3). Data are presented as mean ± SD. ****p<0.0001; ***p<0.001; **p<0.01; *p<0.05; ns, no significance. Parallel tests on the same sample under the same conditions.

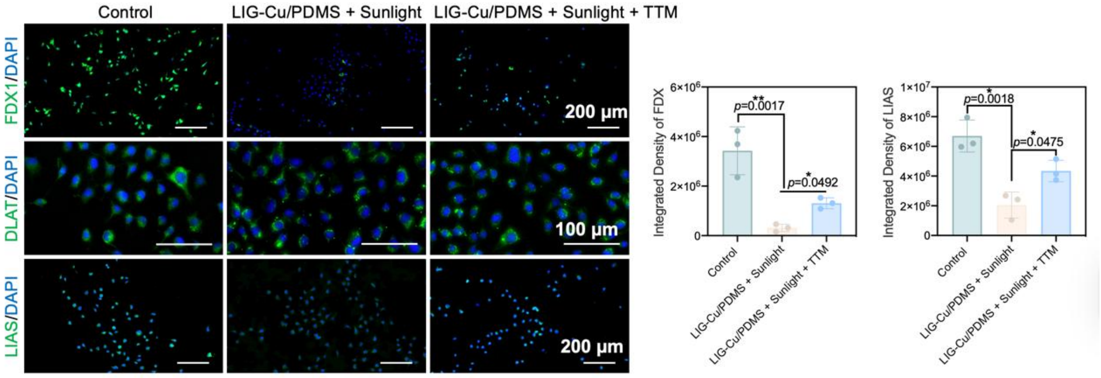

Figure S33. Immunofluorescence images and quantitative analysis of proteins related to cuproptosis in different treatments. Scale bars: 200 μm and 100 μm. Data are presented as mean ± SD. *****p*<0.0001; ****p*<0.001; ***p*<0.01; **p*<0.05; ns, no significance. *n* = 3 biologically independent cell samples per group.

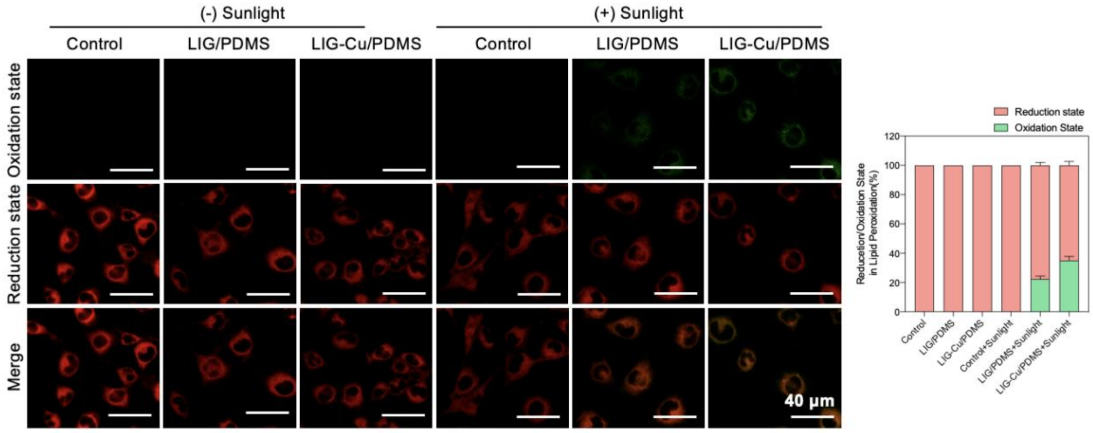

Figure S34. Fluorescence images of B16-F10 cells with various treatments of the BODIPY lipid ROS assay. Scale bars: 40 μm. Data are presented as mean ± SD, n = 3.

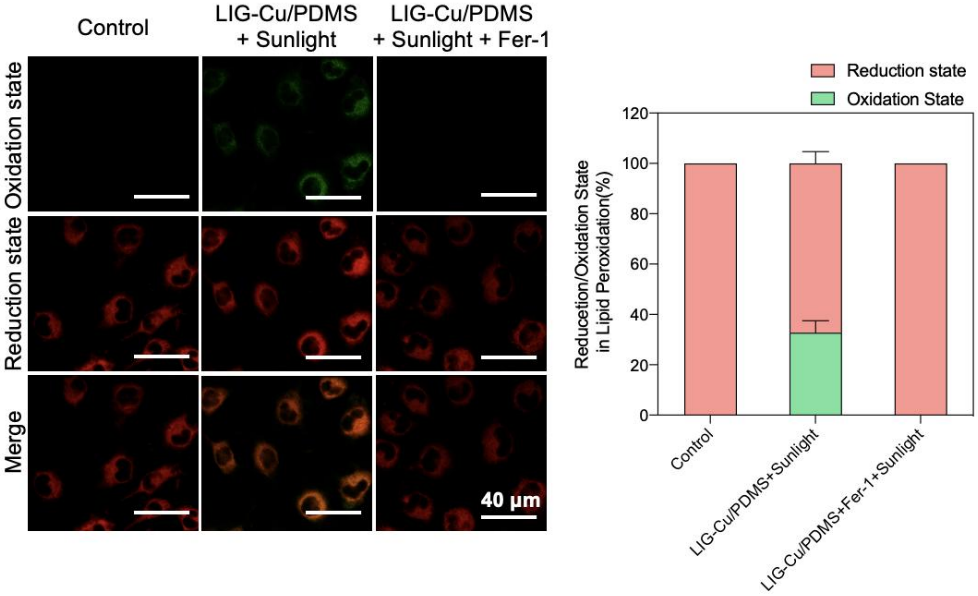

Figure S35. Fluorescence images of B16-F10 cells treated with LIG-Cu/PDMS and Fer-1 of the BODIPY lipid ROS assay. Scale bars: 40 μm. Data are presented as mean ± SD, *n* = 3.

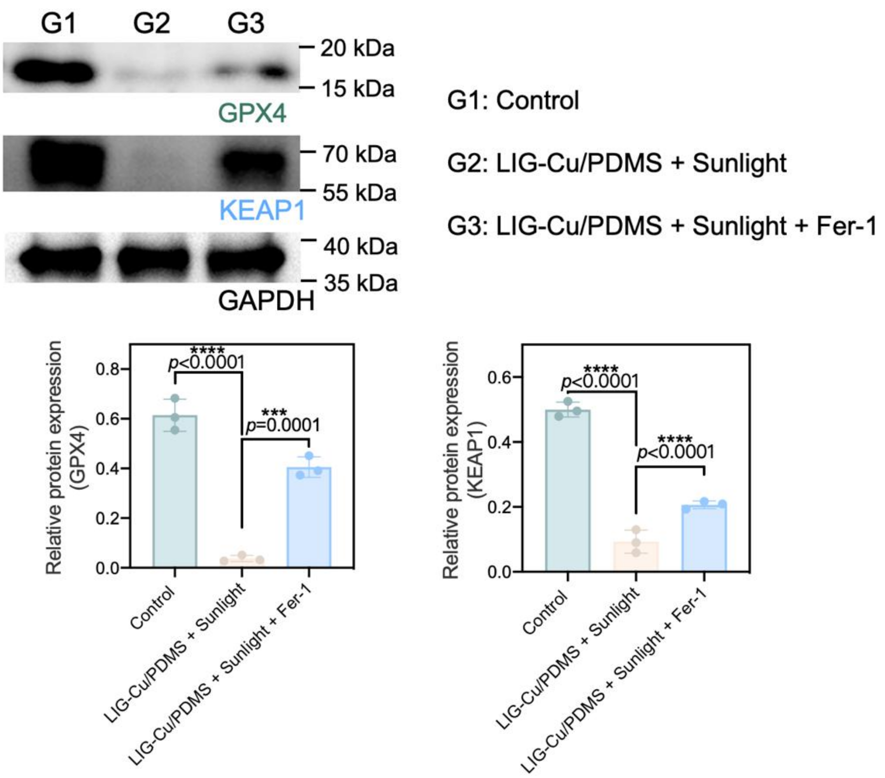

Figure S36. Western blot images and quantitative analysis of proteins related to ferroptosis with LIG-Cu/PDMS + Sunlight and Fer-1. Data are presented as mean ± SD. ****p<0.0001; ***p<0.001; **p<0.01; *p<0.05; ns, no significance. n = 3 biologically independent cell samples per group.

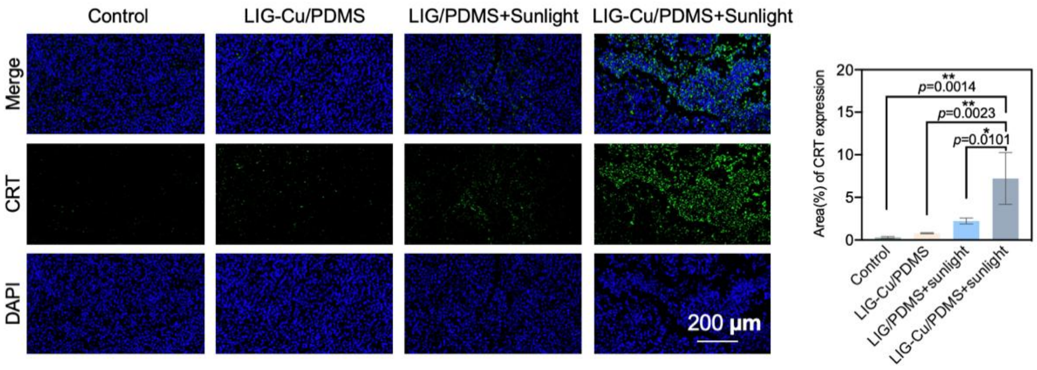

Figure S37. Immunofluorescence images and quantitative analysis of CRT in tumor tissues across different treatment groups (*n* = 3). Data are presented as mean ± SD. *****p*<0.0001; ****p*<0.001; ***p*<0.01; **p*<0.05; ns, no significance.

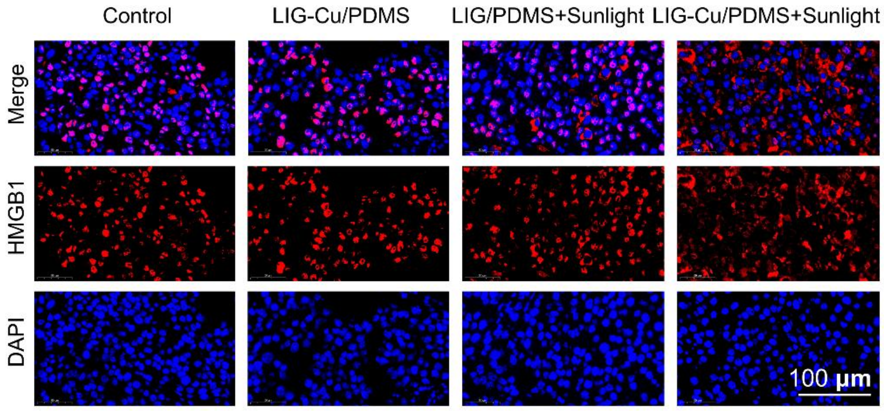

Figure S38. Immunofluorescence images of HMGB1 in tumor tissues across different treatment groups (*n* = 3). Data are presented as mean ± SD.

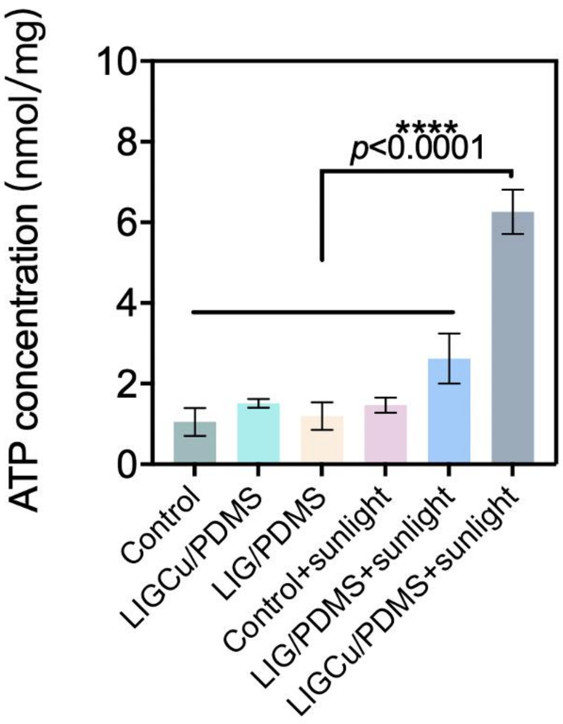

Figure S39. ATP concentration in the cell culture supernatant (*n* = 3). ****p<0.0001; ***p<0.001; **p<0.01; *p<0.05; ns, no significance.

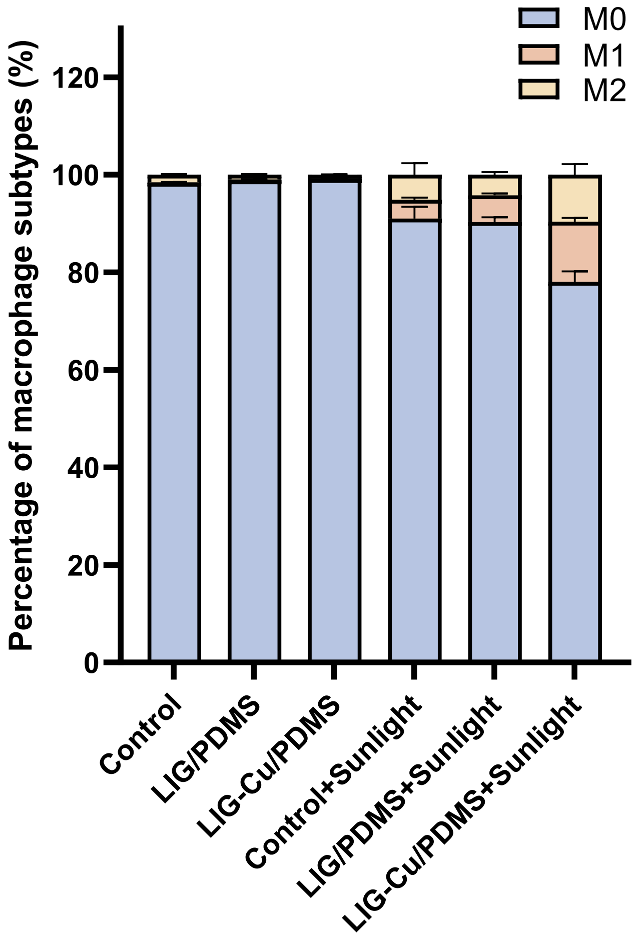

Figure S40. The percentage distribution of macrophage subtypes (*n* = 3). Data are presented as mean ± SD.

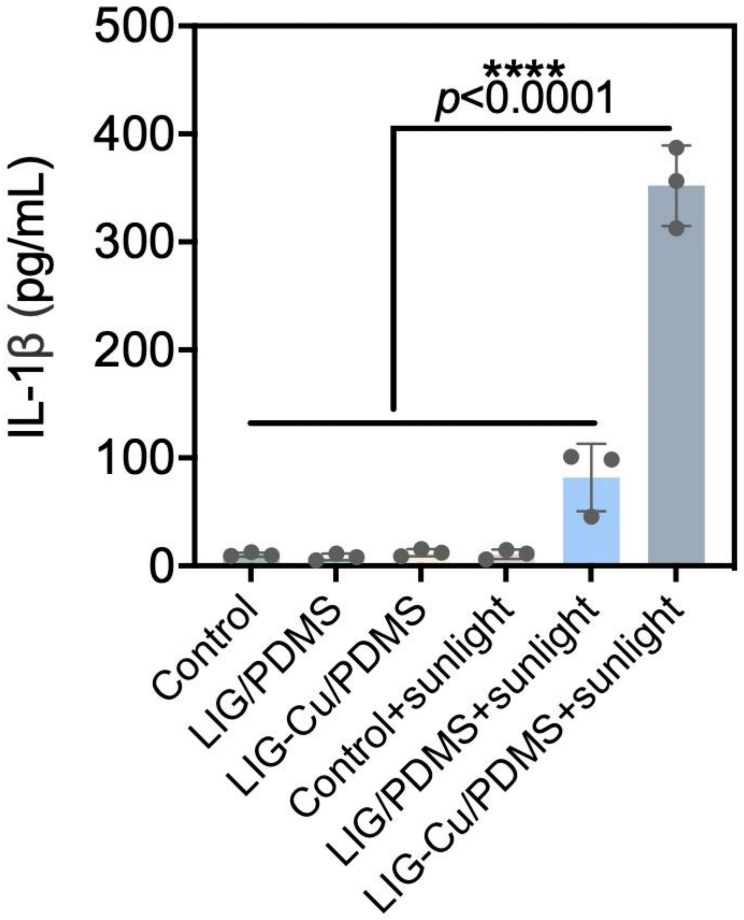

Figure S41. IL-1β secretion by THP-1 treated with different treatments, as measured by ELISA. Data are presented as mean ± SD. ****p<0.0001; ***p<0.001; **p<0.01; *p<0.05; ns, no significance. n = 3 biologically independent cell samples per group.

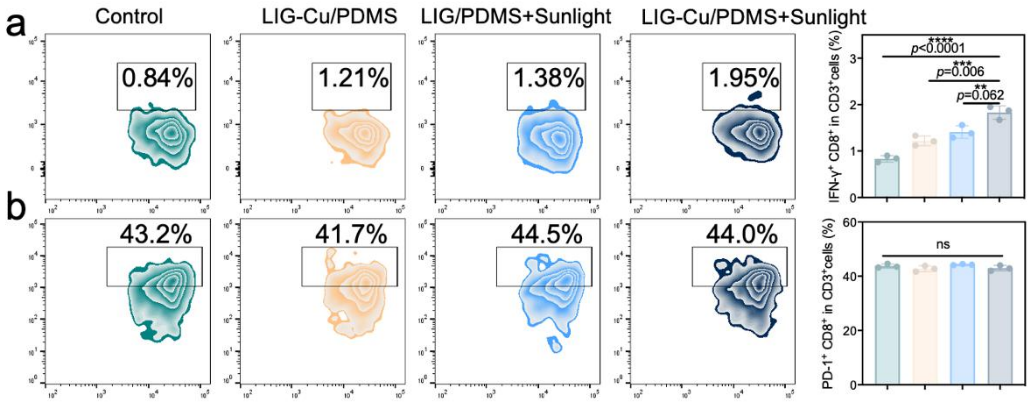

Figure S42. Representative flow cytometry images and quantification of IFN-γ^{+} CD8^{+} CD3^{+}cells (a) PD-1^{+} CD8^{+} CD3^{+}cells (b) under different treatments in the spleen region. Data are presented as mean ± SD, n = 3. ****p<0.0001; ***p<0.001; **p<0.01; *p<0.05; ns, no significance.

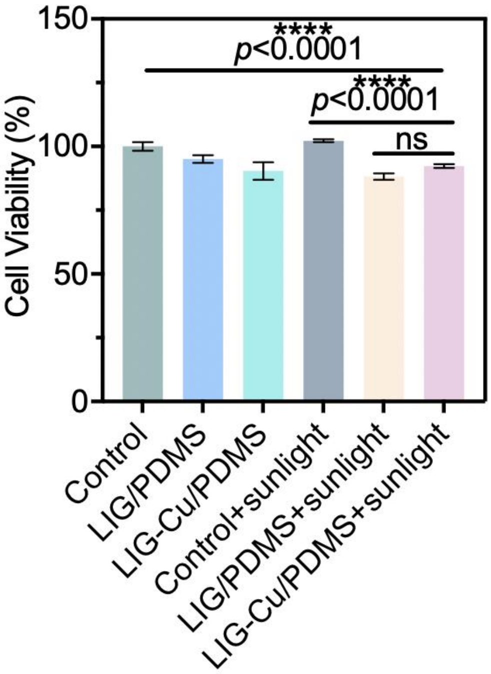

Figure S43. Cell viability of MRC-5 cells against LIG/PDMS and LIG-Cu/PDMS after 60-min stimulation with or without Sunlight. Data are presented as mean ± SD, $n = 3$. ****p<0.0001; ***p<0.001; **p<0.01; *p<0.05; ns, no significance.

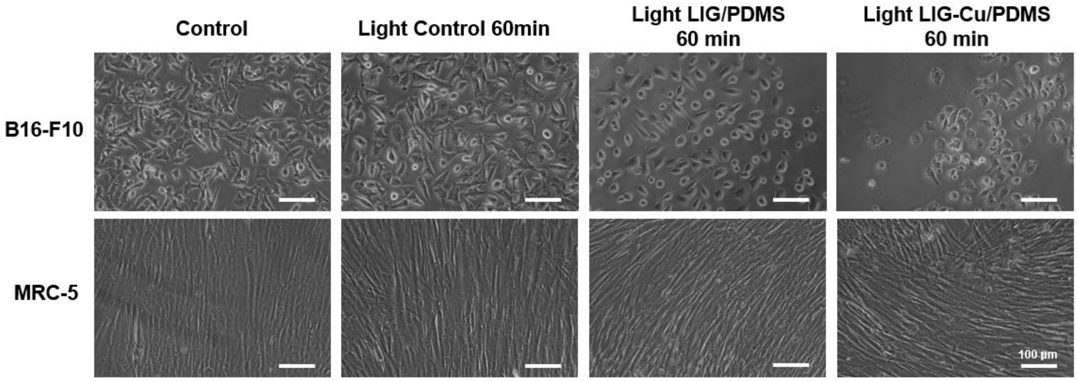

Figure S44. Comparison of antitumor ability and selectivity between LIG/PDMS and LIG-Cu/PDMS *in vitro*. Images of B16-F10 and MRC-5 after incubating with or without LIG/PDMS or LIG-Cu/PDMS under 1.5 kW/m^2 simulated Xenon Sunlight for 60 min or in dark. Scale bars: 100 μm.

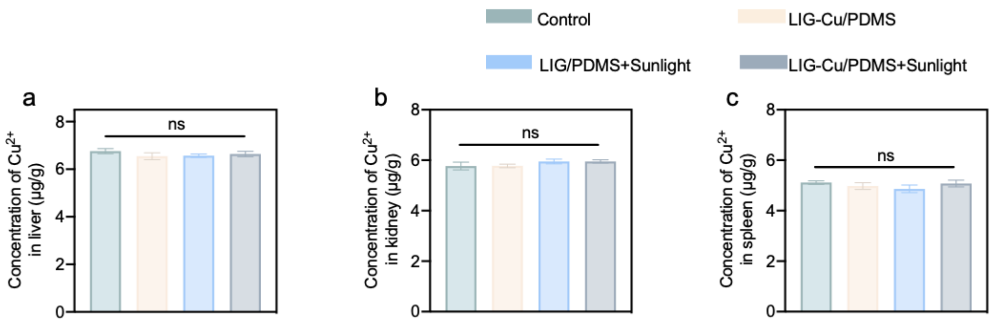

Figure S45. Cu^{2+} accumulation in the liver, kidney, and spleen. Data are presented as mean ± SD, n = 3. ****p<0.0001; ***p<0.001; **p<0.01; *p<0.05; ns, no significance.

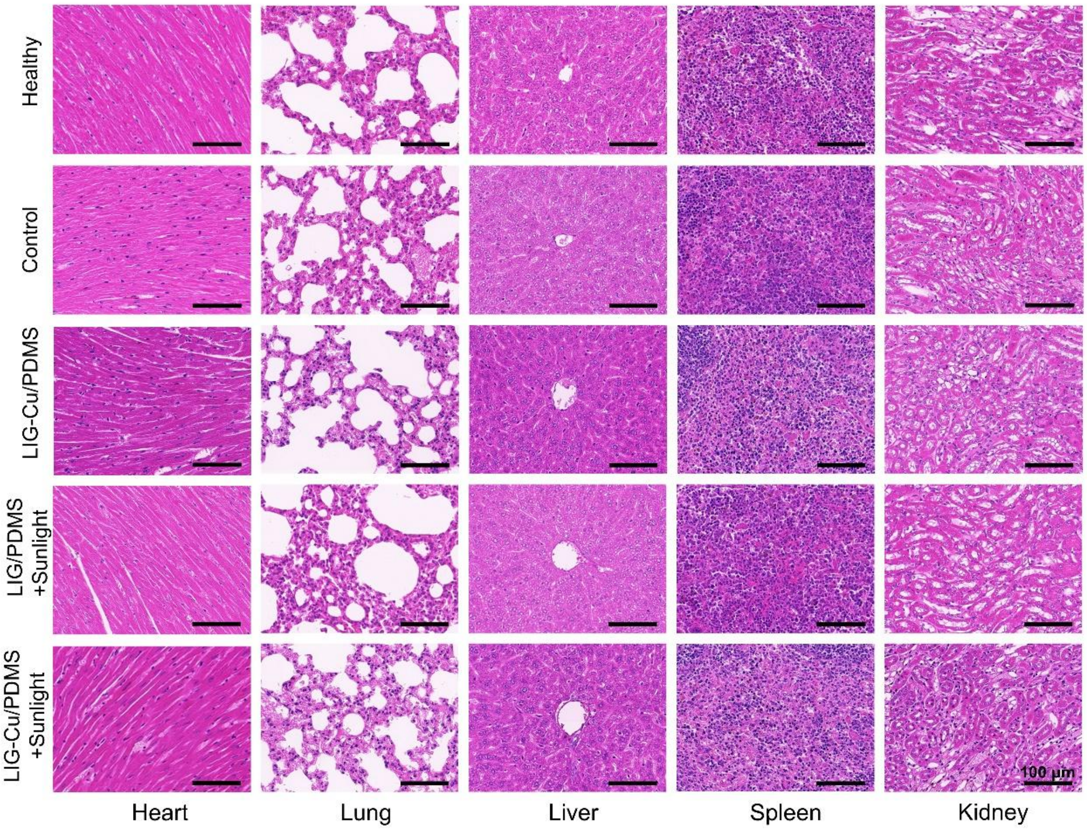

Figure S46. Hematoxylin-eosin staining (H&E staining) analysis of main organs from tumor-bearing mice after different treatments. Scale bars: 100 μm.

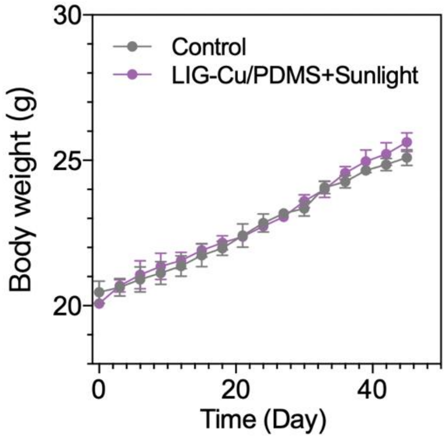

Figure S47. Weight variations in healthy C57BL/6J mice under different treatments. Data are presented as mean ± SD, n = 3.

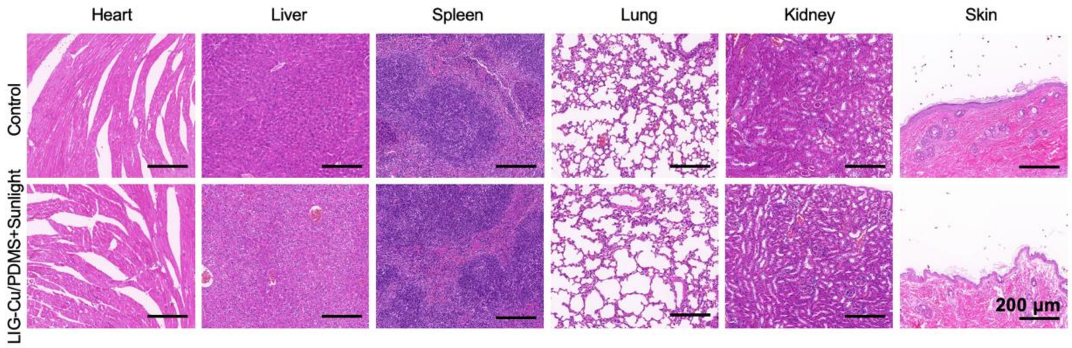

Figure S48. Hematoxylin-eosin staining (H&E staining) analysis of main organs from healthy mice after different treatments. Scale bars: 200 μm.

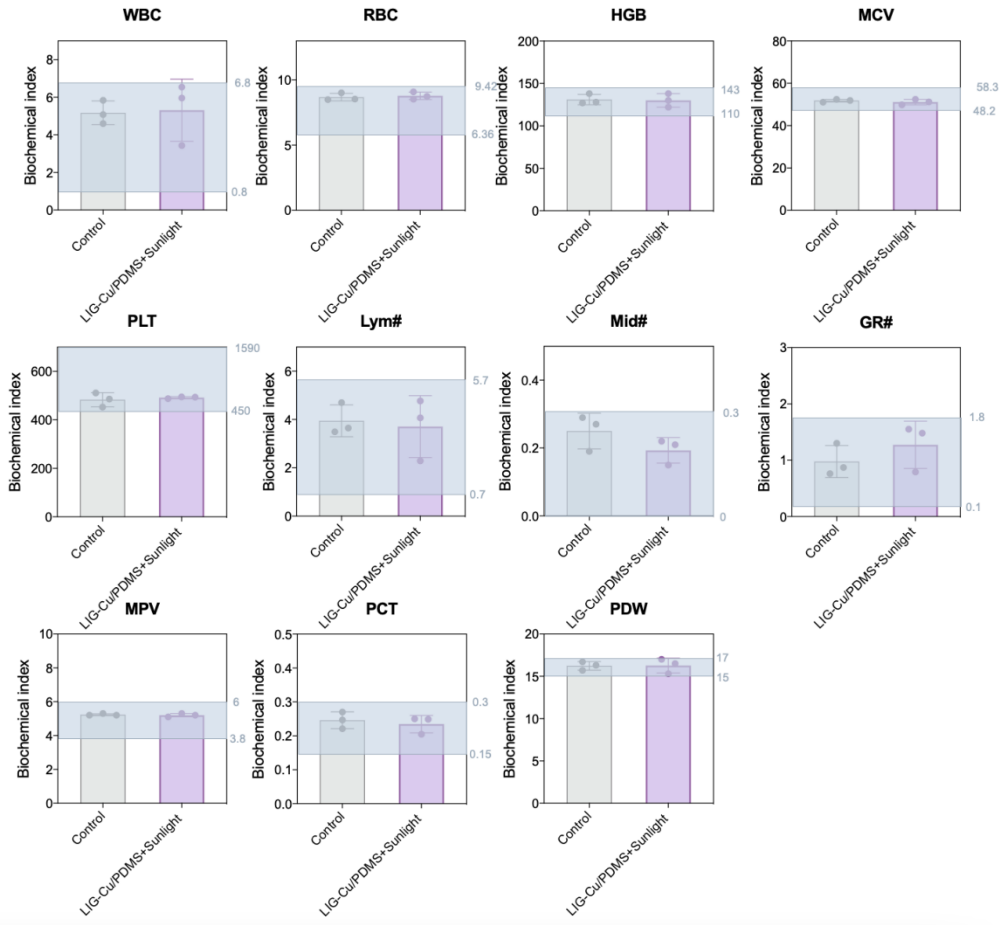

Figure S49. Hematology of healthy mice in the control and the LIG-Cu/PDMS phototherapy group in Day 45. Data are presented as mean ± SD, $n = 3$.

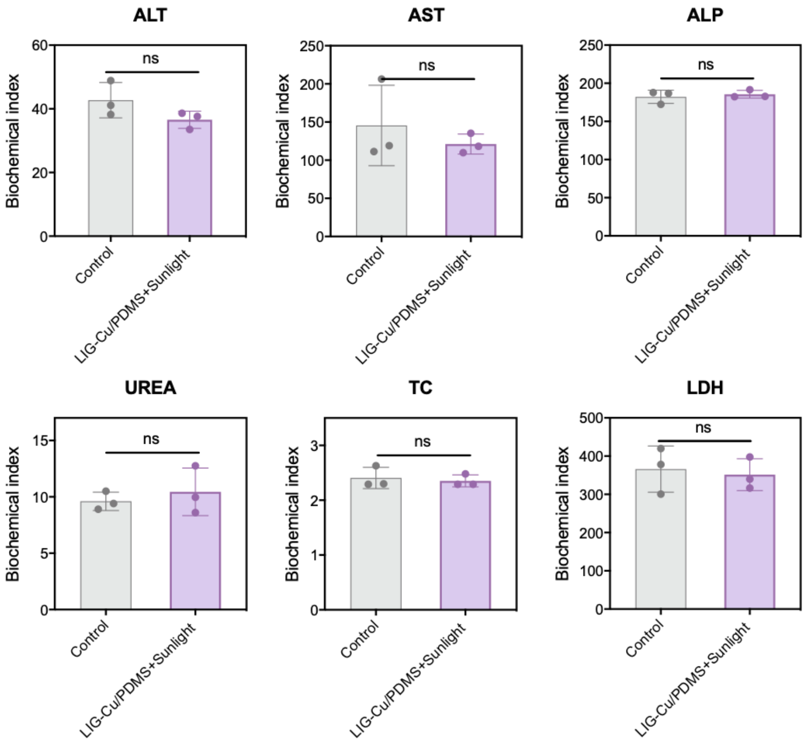

Figure S50. Serum biochemistry of healthy mice in the control and the LIG-Cu/PDMS phototherapy group in Day 45. Data are presented as mean ± SD, $n = 3$. ****p<0.0001; ***p<0.001; **p<0.01; *p<0.05; ns, no significance.

Table S1. Element content of LIG and LIG-Cu samples.

Sample	Element atomic ratio (%)		
	**C**	**O**	**Cu**
LIG	97.75	2.25	/
LIG-Cu	93.58	5.51	0.91

Table S2. Quantitative values for all phases (G0/G1, S, G2/M) of cell cycle.

phase	Control	LIG/PDMS	LIG-Cu/PDMS	Control + Sunlight	LIG/PDMS + Sunlight	LIG-Cu/PDMS + Sunlight
G0/1	48.10±2.01	50.17±0.76	49.9±0.20	49.9±0.70	52.27±0.40	54.33±0.45
S	28.50±1.95	29.37±2.78	30.17±2.66	30.17±3.43	28.97±4.50	18.67±3.20
G2/M	23.7±3.90	20.4±1.95	20.1±2.75	20.03±4.02	18.47±4.15	27.73±4.57

Table S3. Primers used in qRT–PCR for detecting *β-actin, Nectin1, Casp-1, Perp, Gpx3, Irgm1, MSX2.*

qRT-PCR primer	Fp (5′ to 3′)	Rp (5′ to 3′)
β-actin	AACCGCGAGAAGATGACCCAG	GTCACCGGAGTCCATCACGAT
Nectin1	GCTTCAGGGAGAGCCTTACG	GGTTAGCATCCGCTTTGCAC
Casp-1	ATGCCTGGTCTTGTGACTTGG	ATCAGCAGTGGGCATCTGTAG
Perp	GCAACCACATCCAGACATCG	GCAGCCATCGTCGTAGGAG
Gpx3	AAGAT CCATGACATCCGCTGG	TCCATCTTGACGTTGCTGACT
Irgm1	TACCAAACAGGCTCCAGCAG	TGCCACAGTCTCCTTGATTCC
MSX2	ACCACATCCCAGCTTCTAGC	CTTTTCGCCTTAGCCCTTCG

Table S4. Hematology and serum biochemistry mean values for each group of mice on day 15.

Items	Control	LIGCu/PDMS	LIG/PDMS + Sunlight	LIG-Cu/PDMS + Sunlight	Units
WBC	5.93±1.31	3.68±0.36	5.26±0.33	5.05±0.61	10^9/L
RBC	8.41±1.95	6.73±1.91	8.62±0.57	8.34±0.35	10^{12}/L
HGB	138±7.21	114±13	127.67±21.39	118.67±10.97	g/L
MCV	51.7±2.78	50.67±2.45	49.8±3.21	48.7±2.34	fL
PLT	741.33±85.17	598.33±72.54	693.33±64.67	677±80.73	10^9/L
